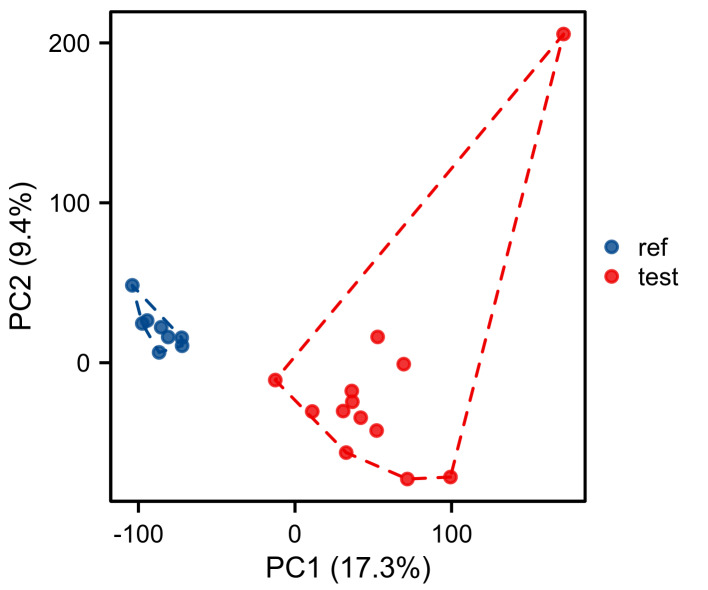# Identification and validation of oxidative stress-related genes in sepsis-induced myopathy

**DOI:** 10.1097/MD.0000000000037933

**Published:** 2024-05-03

**Authors:** Ning Zhang, Dan Huang, Xiang Li, JinXia Yan, Qi Yan, WeiXing Ge, Jun Zhou

**Affiliations:** a Intensive Care Unit, the Affiliated Jiangning Hospital of Nanjing Medical University, Nanjing, China; b Department of Ophthalmology, the First Affiliated Hospital with Nanjing Medical University, Nanjing, China.

**Keywords:** bioinformatics, biomarkers, GEO, oxidative stress-related genes, sepsis-induced myopathy

## Abstract

**Background:**

Sepsis-induced myopathy (SIM) a complication of sepsis that results in prolonged mechanical ventilation, long-term functional disability, and increased patient mortality. This study was performed to identify potential key oxidative stress-related genes (OS-genes) as biomarkers for the diagnosis of SIM using bioinformatics.

**Methods:**

The GSE13205 was obtained from the Gene Expression Omnibus (GEO) database, including 13 SIM samples and 8 healthy samples, and the differentially expressed genes (DEGs) were identified by limma package in R language. Simultaneously, we searched for the genes related to oxidative stress in the Gene Ontology (GO) database. The intersection of the genes selected from the GO database and the genes from the GSE13205 was considered as OS-genes of SIM, where the differential genes were regarded as OS-DEGs. OS-DEGs were analyzed using GO enrichment, Kyoto Encyclopedia of Genes and Genomes (KEGG) pathways, and protein-protein interaction (PPI) networks. Hub genes in OS-DEGs were selected based on degree, and diagnostic genes were further screened by gene expression and receiver operating characteristic (ROC) curve. Finally, a miRNA-gene network of diagnostic genes was constructed.

**Results:**

A total of 1089 DEGs were screened from the GSE13205, and 453 OS-genes were identified from the GO database. The overlapping DEGs and OS-genes constituted 25 OS-DEGs, including 15 significantly upregulated and 10 significantly downregulated genes. The top 10 hub genes, including CD36, GPX3, NQO1, GSR, TP53, IDH1, BCL2, HMOX1, JAK2, and FOXO1, were screened. Furthermore, 5 diagnostic genes were identified: CD36, GPX3, NQO1, GSR, and TP53. The ROC analysis showed that the respective area under the curves (AUCs) of CD36, GPX3, NQO1, GSR, and TP53 were 0.990, 0.981, 0.971, 0.971, and 0.971, which meant these genes had very high diagnostic values of SIM. Finally, based on these 5 diagnostic genes, we found that miR-124-3p and miR-16-5p may be potential targets for the treatment of SIM.

**Conclusions:**

The results of this study suggest that OS-genes might play an important role in SIM. CD36, GPX3, NQO1, GSR, and TP53 have potential as specific biomarkers for the diagnosis of SIM.

## 1. Introduction

Sepsis-induced myopathy (SIM) is a complication of sepsis that leads to extended periods of mechanical ventilation, persistent functional impairment, and increased patient mortality.^[1]^ The presence of SIM in patients has a significant impact on their quality of life because to the notable decline in peripheral muscular strength, which includes the weakening of respiratory muscles. Respiratory failure may occur in SIM due to the affected balance between the work of breathing and the burden on respiratory muscles.^[2,3]^ The diminished power of peripheral muscles has a negative impact on the day-to-day functioning of individuals, leading to heightened mortality rates among people diagnosed with heart failure.^[4]^

Prior clinical investigations on the management of SIM predominantly concentrated on the mitigation of sepsis-related mortality. The death rates associated with sepsis have been notably decreased in the intensive care unit (ICU) due to advancements and enhancements in life support systems. As a result, there have been a rise in the occurrence of SIM among survivors.^[5]^

The timely and precise identification of SIM is crucial in order to promptly implement efficacious evidence-based medical interventions and therapies. However, the current diagnosis of SIM primarily includes clinical evaluation and electrophysiological examination.^[6]^ Moreover, there is no established treatment for SIM, which mainly includes early functional exercise, nutritional structural adjustment, and functional electrical stimulation, etc.^[7]^ Therefore, finding effective ways to diagnose and treat SIM is an urgent problem for modern medicine.

In recent years, there has been scholarly investigation into the etiology of SIM, leading to the conclusion that many elements, such as microcirculatory irregularities, endothelial cell injury, E-selectin, and energy metabolism, significantly contribute to the development of SIM.^[6]^ The observed phenomenon could potentially be associated with peripheral microcirculatory problems, the impact of inflammatory factors, neuroedema, electrolyte imbalances, and the hypermetabolic state commonly observed in patients with sepsis.^[8]^ Mitochondrial dysfunction could potentially serve as an additional etiological factor contributing to SIM, and may be associated with compromised cellular energy supply and increased oxidative stress.^[9]^ Research has indicated that mitochondrial destruction is a possible source of cellular harm, and the release of mitochondrial damage-related patterns (such as mitochondrial DNA) activates innate immune responses and can cause further oxidative stress damage. Excessive ROS can directly damage cell membranes, disrupt mitochondrial activity, and harm proteins and DNA.^[10,11]^ Thus, the severity of SIM is strongly associated with skeletal muscle mitochondrial dysfunction, depletion of ATP, depletion of glutathione, and generation of nitric oxide. Both nitric oxide and reactive oxygen species (ROS) have been found to interfere with the functioning of the type I complex respiratory chain, the labile diffusion of intracellular antioxidants leads to a decrease in glutathione levels. Additionally, the inhibition of the type I complex respiratory chain function results in reduced mitochondrial ATP production, mitochondrial dysfunction further enhances the production of ROS in sepsis. These evidences highlight the significant role of oxidative stress in the development of skeletal muscle damage during sepsis.^[12–14]^

In the present study, a dataset containing 13 SIM samples and 8 healthy samples were downloaded from the Gene Expression Omnibus (GEO) database (https://www.ncbi.nlm.nih.gov/geo/). In addition, OS-genes were screened from the Gene Ontology (GO) database (http://geneontology.org/). Next, the identification of differentially expressed genes (DEGs) and the determination of gene overlap between DEGs and OS-genes were performed. Subsequently, differentially expressed oxidative stress-related genes (OS-DEGs) were analyzed using bioinformatics methods, including GO term enrichment, the Kyoto Encyclopedia of Genes and Genomes (KEGG), and protein-protein interaction (PPI) network analysis. We then used receiver operating characteristic (ROC) curves to evaluate the area under the curve (AUC) value and predictive ability of the hub genes to identify the diagnostic genes.

## 2. Materials and methods

### 2.1. Data acquisition

**Figure 1.**
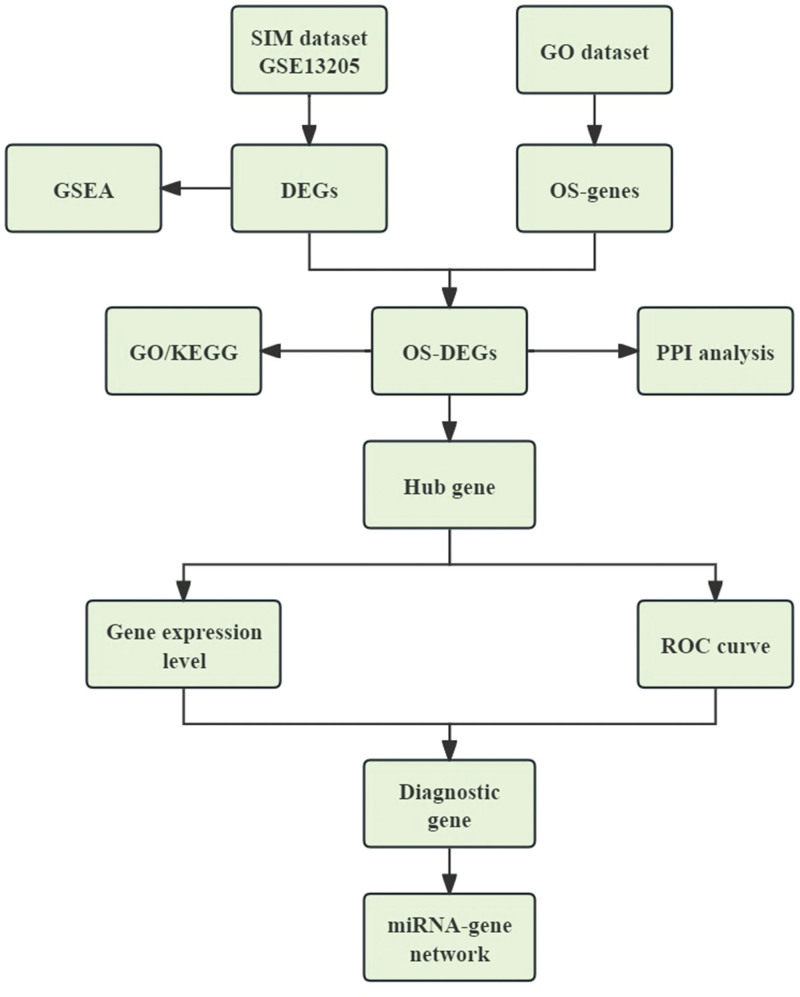
The flow chart of this study. DEGs, differentially expressed genes screened from GSE13205; OS-genes, oxidative stress-related genes downloaded from Gene Ontology database. GO = Gene Ontology, GSEA = gene set enrichment analysis, KEGG = Kyoto Encyclopedia of Genes and Genomes, OS-DEGs = differentially expressed oxidative stress-related genes, ROC = receiver operating characteristic.

A total of 453 OS-genes were obtained from the GO database (http://geneontology.org/), and use “Homo sapiens” as a screening condition to collect genes related to oxidative stress; all OS-genes are listed in Table S1, Supplemental Digital Content, http://links.lww.com/MD/M267. The gene expression profile of GSE13205 was downloaded from the GEO database (http://www.ncbi.nlm.nih.gov/geo), which is a database repository of high-throughput gene expression data, hybridization arrays, chips, and microarrays. GSE13205 was based on platform GPL570 [(HG-U133_Plus_2) Affymetrix Human Genome U133 Plus 2.0 Array] (Thermofisher Scientific, Waltham, MA). The dataset consisted of 13 SIM samples and 8 healthy samples. The muscle biopsies were taken from the lateral portion of the vastus lateralis muscle, 10 to 20 centimeters above the knee in patients with or without SIM. The scale function in R version 4.2.1 software was used to perform quality control and normalization of these 2 gene expression profiles, which are represented by boxplots. Principal component analysis (PCA) was used to verify the reproducibility of the data, and the R package ggord was used to construct the PCA plots. Figure 1 depicts the flow chart of this study.

GEO belongs to public databases. The patients involved in the database have obtained ethical approval. Users can download relevant data for free for research and publish relevant articles. Our study is based on open-source data, so there are no ethical issues and other conflicts of interest.

### 2.2. Analysis of differentially expressed genes

Differential expression analysis was conducted using the “limma” package in R 4.2.1 software, and “ggplot2” and “ComplexHeatmap” were used to depict volcano/difference ranking plots and heatmap plots, respectively. GSEA was conducted using GSEA software (version 4.1.0) to clarify the potential mechanism in SIM. The DEGs were screened using the criteria of a |log2(FC)| > 1 and *P* value < .05, and the enriched pathways of GSEA were screened using an FDR < 0.25 and a *P*.adjust < .05.

### 2.3. Analysis of differential expression of oxidative stress-related genes

The “ggplot2” and “VennDiagram” package in R 4.2.1 software was adopted to draw the intersection of DEGs and OS-genes, i.e., OS-DEGs. The KEGG pathway and GO enrichment of OS-DEGs were analyzed utilizing the “clusterProfiler” packages of R software. An analysis of the PPI network of OS-DEGs was performed using the STRING database (https://string-db.org/), which covered almost all functional interactions between the expressed proteins, and interaction with a combined score > 0.4 was considered statistically significant. The results of this analysis were visualized with Cytoscape (version 3.8.2) software. Through the Molecular Complex Detection (MCODE) plug-in of Cytoscape software, the most closely connected modules were selected from the PPI network for further analysis (setting parameters as degree cutoff = 2, node score = 0.2, k-core = 2, maximum depth = 100). Furthermore, we used cytoHubba, a plug-in of Cytoscape software, to filter the hub genes from the whole PPI network and calculated it by the Degree method. The expression levels and Spearman correlations of OS-DEGs are displayed.

### 2.4. Identification of diagnostic genes

To screen the diagnostic genes, we visually displayed the expression levels of hub genes between SIM patients and healthy controls in the form of scatter plots and boxplots. ROC curve analysis was performed, and the AUCs were calculated using the pROC package in R software to determine the predicted values of the hub genes. Diagnostic genes were selected from the hub genes using the criterion of AUC > 0.900.

### 2.5. Construction of an miRNA-gene regulatory network

The miRTarBase database (https://mirtarbase.cuhk.edu.cn/) was applied to predict the interaction between diagnostic genes and miRNAs, and the miRNA-gene regulatory network was visualized using Cytoscape software.

### 2.6. Statistical analysis

R 4.2.1 software were employed in this research. Data are presented as the mean ± SD, and comparisons between groups were performed using an unpaired Student *t* test. ROCs were used to evaluate AUCs and predictive abilities. A *P* value of less than .05 was considered statistically significant.

## 3. Results

### 3.1. Identification of DEGs and OS-DEGs in SIM

**Figure 2.**
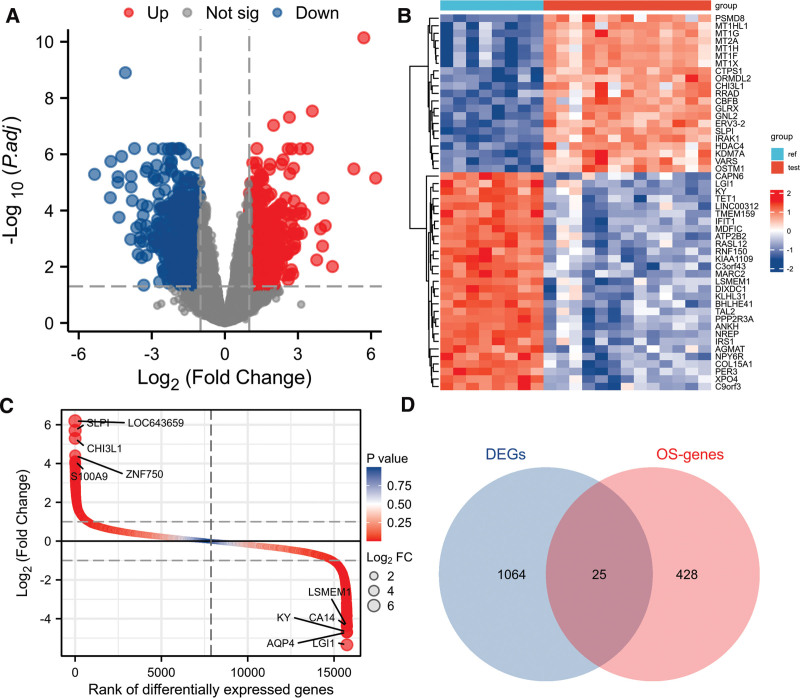
Identification of DEGs and OS-DEGs in SIM muscular tissue in the GSE13205 database. (A) Volcano plot, (B) heatmap, and (C) difference ranking plot of DEGs between the SIM and control samples. (D) Venn diagram of overlapping genes between DEGs and OS-genes. DEGs = differentially expressed genes, OS-genes = oxidative stress-related genes.

Normalization was performed on the expression matrices of the GSE13205, and the distribution trends of the boxplots were large straight lines (Figure S1, Supplemental Digital Content, http://links.lww.com/MD/M265). The PCA revealed that the data were repeatable (Figure S2, Supplemental Digital Content, http://links.lww.com/MD/M266). Using the thresholds of adjusted |log2(FC)| > 1 and *P* value < .05, a total of 1089 DEGs were obtained from the GSE13205, including 494 significantly upregulated and 595 significantly downregulated genes (Table S2, Supplemental Digital Content, http://links.lww.com/MD/M268). Volcano plots and heatmaps were used to visualize DEGs (Fig. 2A and B), and the top 5 up- and downregulated genes were marked in the difference ranking plot (Fig. 2C).

**Figure 3.**
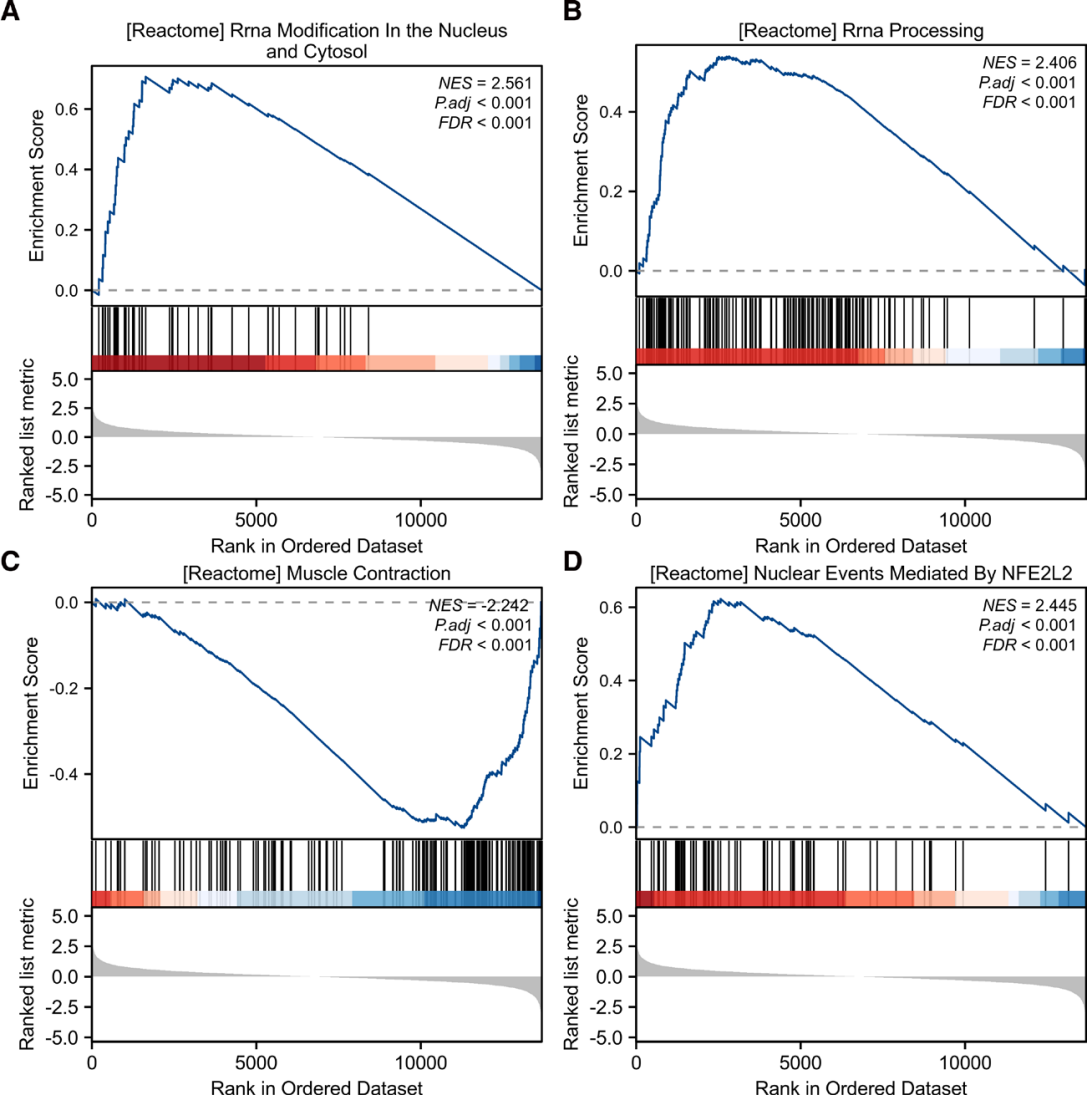
Enrichment analyses using gene set enrichment analysis (GSEA). Four significant gene set enrichment pathways (FDR < 0.25, adjusted *P* < .05). (A) Reactome rRNA modification ln the nucleus. (B) rRNA processing. (C) Reactome muscle contraction. (D) Reactome nuclear events mediated by NFE2L2.

**Figure 4.**
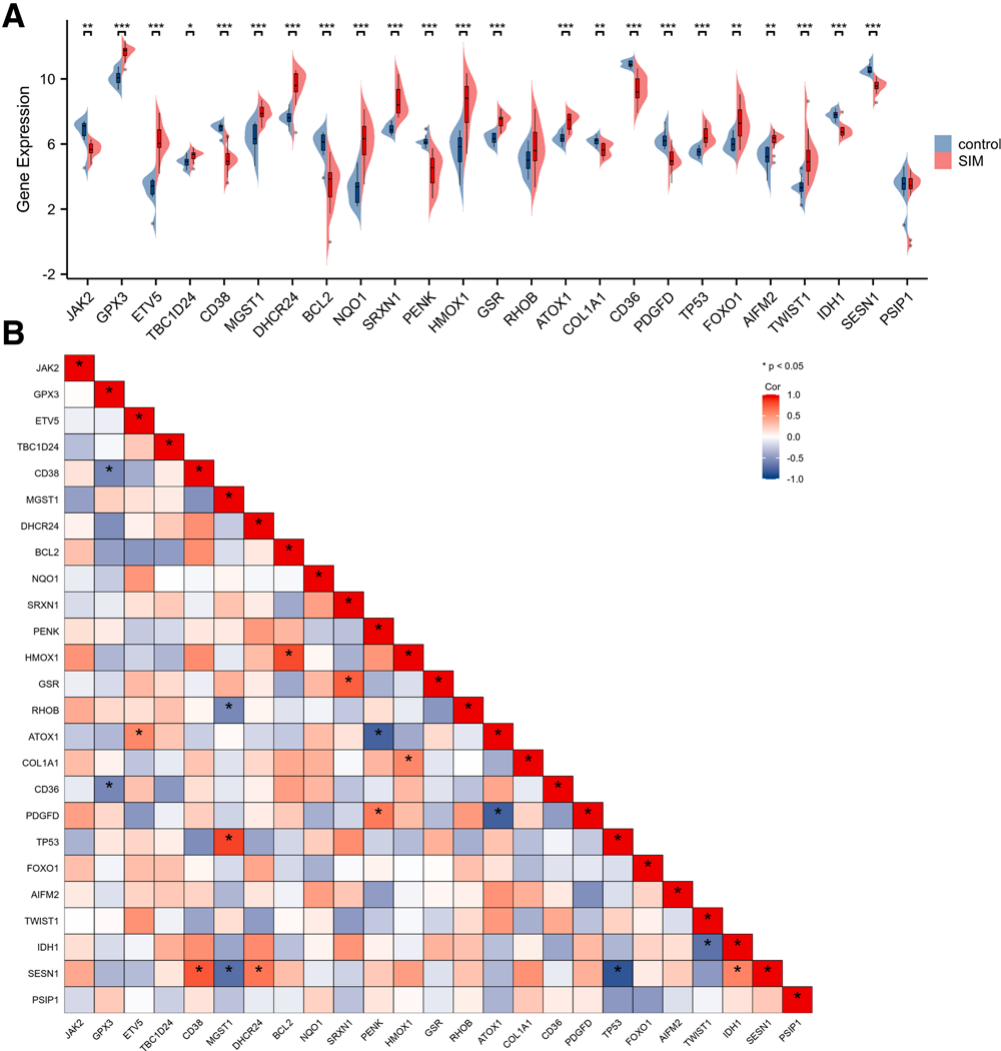
Expression and correlation analysis of 25 OS-DEGs. (A) Expression levels of OS-DEGs in the SIM and control samples were visualized by box plot.*P < .05, **P < .01, ***P < .001. (B) Spearman correlation among 25 OS-DEGs. *P < .05. OS-DEGs = differentially expressed oxidative stress-related genes, SIM = sepsis-induced myopathy.

The GSEA showed that DEGs were mainly involved in the Reactome rRNA modification ln the nucleus, rRNA processing, Reactome muscle contraction, and Reactome nuclear events mediated by NFE2L2 (Fig. 3A–D). Ultimately, combined with DEGs and OS-genes, we screened 25 overlapping genes, termed OS-DEGs, for further study (Fig. 2D), including 15 significantly upregulated and 10 significantly downregulated genes. Figure 4A and B show the expression levels and correlations of the 25 OS-DEGs. Table 1 shows the information for the 25 OS-DEGs roles in oxidative stress.

**Table 1:** Twenty-five differentially expressed oxidative stress-related genes.

Gene symbol	Description	Expression	Roles in oxidative stress
COL1A1	collagen type I alpha 1 chain	Downregulated	Collagen alpha-1(I) chain
PENK	proenkephalin	Downregulated	Proenkephalin-A
CD38	CD38 molecule	Downregulated	ADP-ribosyl cyclase/cyclic ADP-ribose hydrolase 1
BCL2	BCL2, apoptosis regulator	Downregulated	Apoptosis regulator Bcl-2
CD36	CD36 molecule	Downregulated	Platelet glycoprotein 4
PDGFD	platelet derived growth factor D	Downregulated	Platelet-derived growth factor D
IDH1	isocitrate dehydrogenase (NADP(+)) 1, cytosolic	Downregulated	Isocitrate dehydrogenase [NADP] cytoplasmic
SESN1	sestrin 1	Downregulated	Sestrin-1
JAK2	Janus kinase 2	Downregulated	Tyrosine-protein kinase JAK2
PSIP1	PC4 and SFRS1 interacting protein 1	Downregulated	PC4 and SFRS1-interacting protein
ATOX1	antioxidant 1 copper chaperone	Upregulated	Copper transport protein ATOX1
TP53	tumor protein p53	Upregulated	Cellular tumor antigen p53
AIFM2	apoptosis inducing factor, mitochondria associated 2	Upregulated	Ferroptosis suppressor protein 1
TBC1D24	TBC1 domain family member 24	Upregulated	TBC1 domain family member 24
GSR	glutathione-disulfide reductase	Upregulated	Glutathione reductase, mitochondrial
RHOB	ras homolog family member B	Upregulated	Rho-related GTP-binding protein RhoB
FOXO1	forkhead box O1	Upregulated	Forkhead box protein O1
GPX3	glutathione peroxidase 3	Upregulated	Glutathione peroxidase 3
MGST1	microsomal glutathione S-transferase 1	Upregulated	Microsomal glutathione S-transferase 1
SRXN1	sulfiredoxin 1	Upregulated	Sulfiredoxin-1
NQO1	NAD(P)H quinone dehydrogenase 1	Upregulated	NAD(P)H dehydrogenase [quinone] 1
TWIST1	twist family bHLH transcription factor 1	Upregulated	Twist-related protein 1
DHCR24	24-dehydrocholesterol reductase	Upregulated	Delta(24)-sterol reductase
ETV5	ETS variant 5	Upregulated	ETS translocation variant 5
HMOX1	heme oxygenase 1	Upregulated	Heme oxygenase 1

### 3.2. Functional enrichment analysis of OS-DEGs

**Figure 5.**
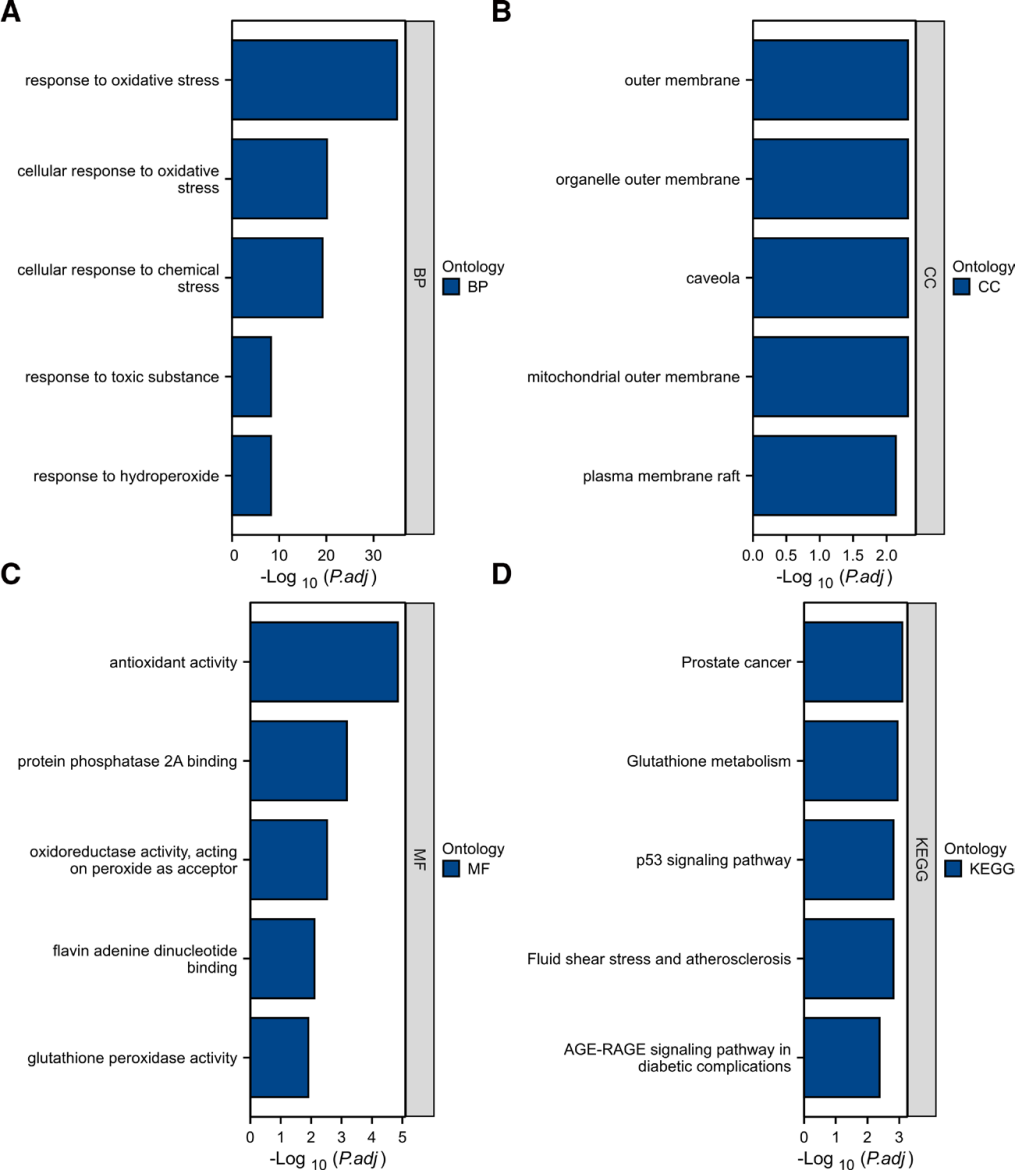
Bar plots of 25 OS-DEGs-enriched GO terms and KEGG pathways. Panels (A–D) represent BP, CC, MF, and KEGG, respectively. BP = biological process, CC = cellular component, GO = Gene Ontology, KEGG = Kyoto Encyclopedia of Genes and Genomes, MF = molecular function, OS-DEGs = differentially expressed oxidative stress-related genes.

**Figure 6.**
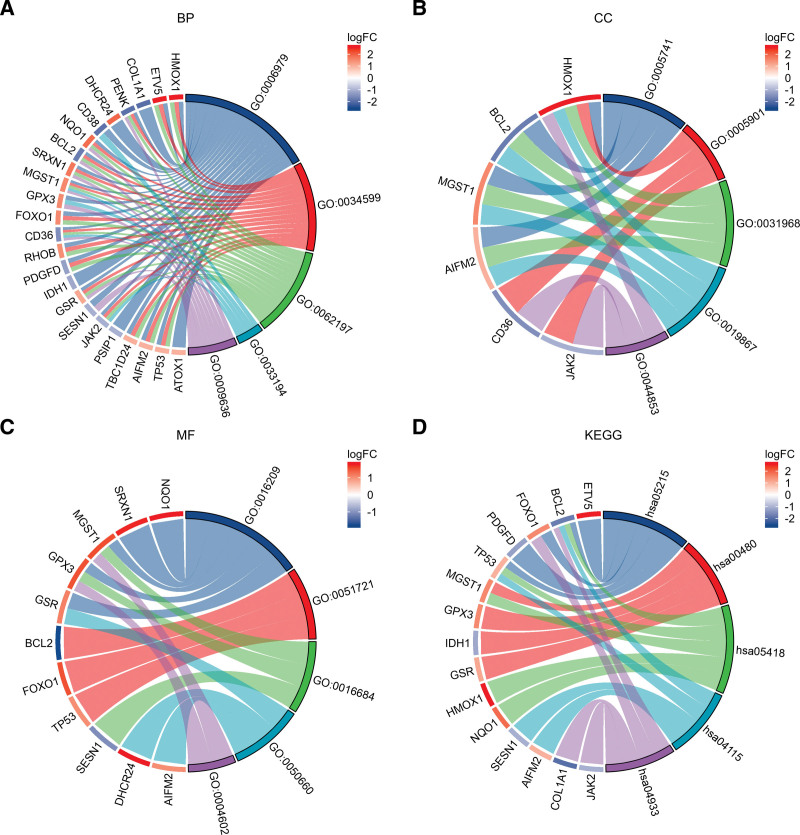
Chord plots of 25 OS-DEGs-enriched GO terms and KEGG pathways. Panels (A–D) represent the top 5 enriched items and their enriched genes of BP, CC, MF, and KEGG, respectively. BP = biological process, CC = cellular component, GO = Gene Ontology, KEGG = Kyoto Encyclopedia of Genes and Genomes, MF = molecular function, OS-DEGs = differentially expressed oxidative stress-related genes.

Gene Ontology and KEGG analyses were conducted to reveal the possible biological functions and enrichment pathways of OS-DEGs. The GO analysis was categorized into biological processes (BPs), cell components (CCs), and molecular functions (MFs). The BP of OS-DEGs were mainly enriched in response to oxidative stress, cellular response to oxidative stress, cellular response to chemical stress. For CC, OS-DEGs were mainly enriched in the mitochondrial outer membrane, caveola and organelle outer membrane. The OS-DEGs were enriched in MF, including antioxidant activity, protein phosphatase 2A binding and oxidoreductase activity, acting on peroxide as acceptor. KEGG analysis showed that OS-DEGs were mainly associated with prostate cancer, glutathione metabolism and fluid shear stress and atherosclerosis. Figures 5 show the top 5 enrichment items and (Fig. 6A–D) show the enriched genes of BP, CC, MF, and KEGG.

### 3.3. Analysis of PPI networks and identification of hub genes

**Figure 7.**
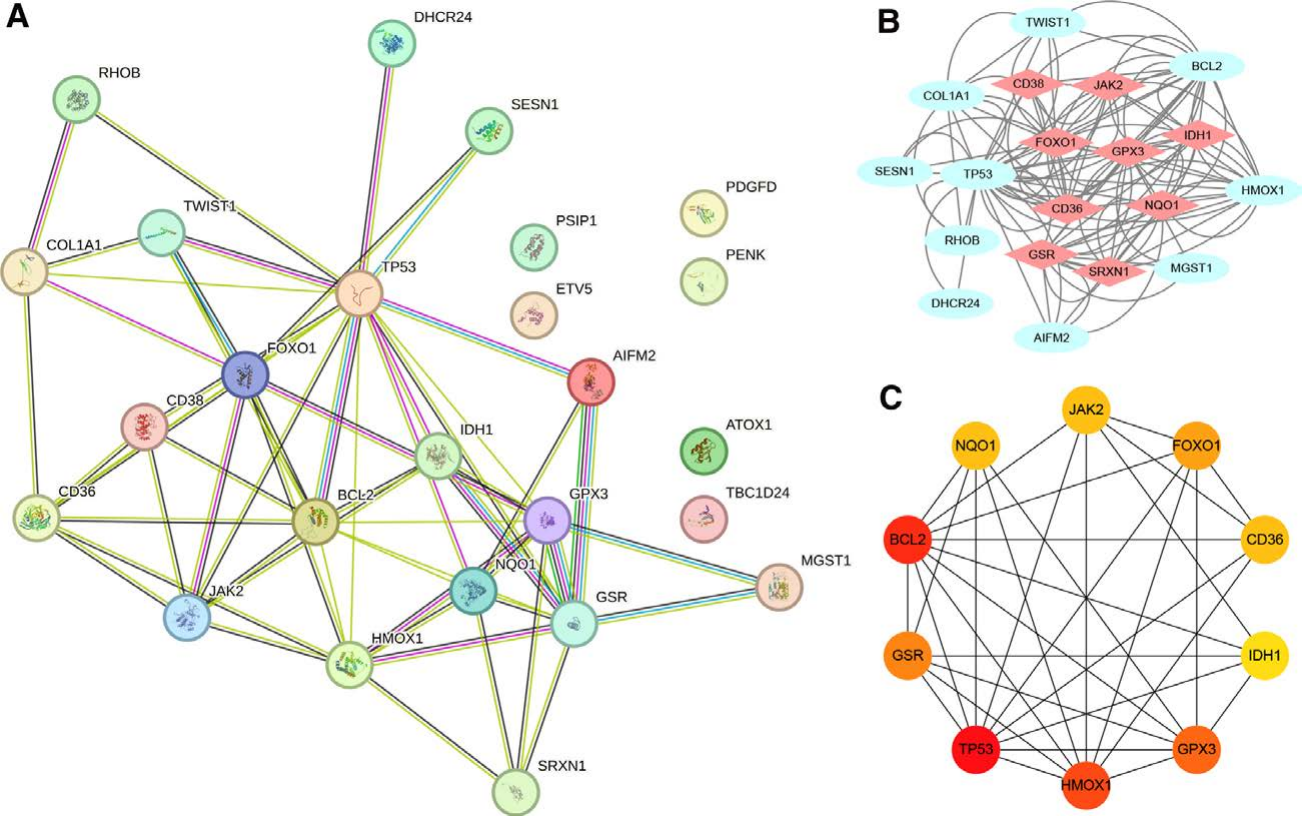
Protein-protein interaction network, most significant module and hub genes. (A) Protein-protein interaction network constituted with the OS-DEGs; (B) The most significant module was obtained from PPI network and marked in light red. (C) top 10 hub genes. Darker colors indicate a higher value. OS-DEGs = differentially expressed oxidative stress-related genes.

The STRING database was used to construct the PPI network to identify the interactive relationships between OS-DEGs. A total of 25 nodes and 116 edges were identified in the PPI network (Fig. 7A). The most significant module was obtained using the MCODE plug-in of Cytoscape (Fig. 7B). The Cytohubba plug-in in Cytoscape software was then used to select the top 10 hub genes based on their degree of connectivity (Fig. 7C).

### 3.4. Identification and validation of diagnostic feature biomarkers

**Figure 8.**
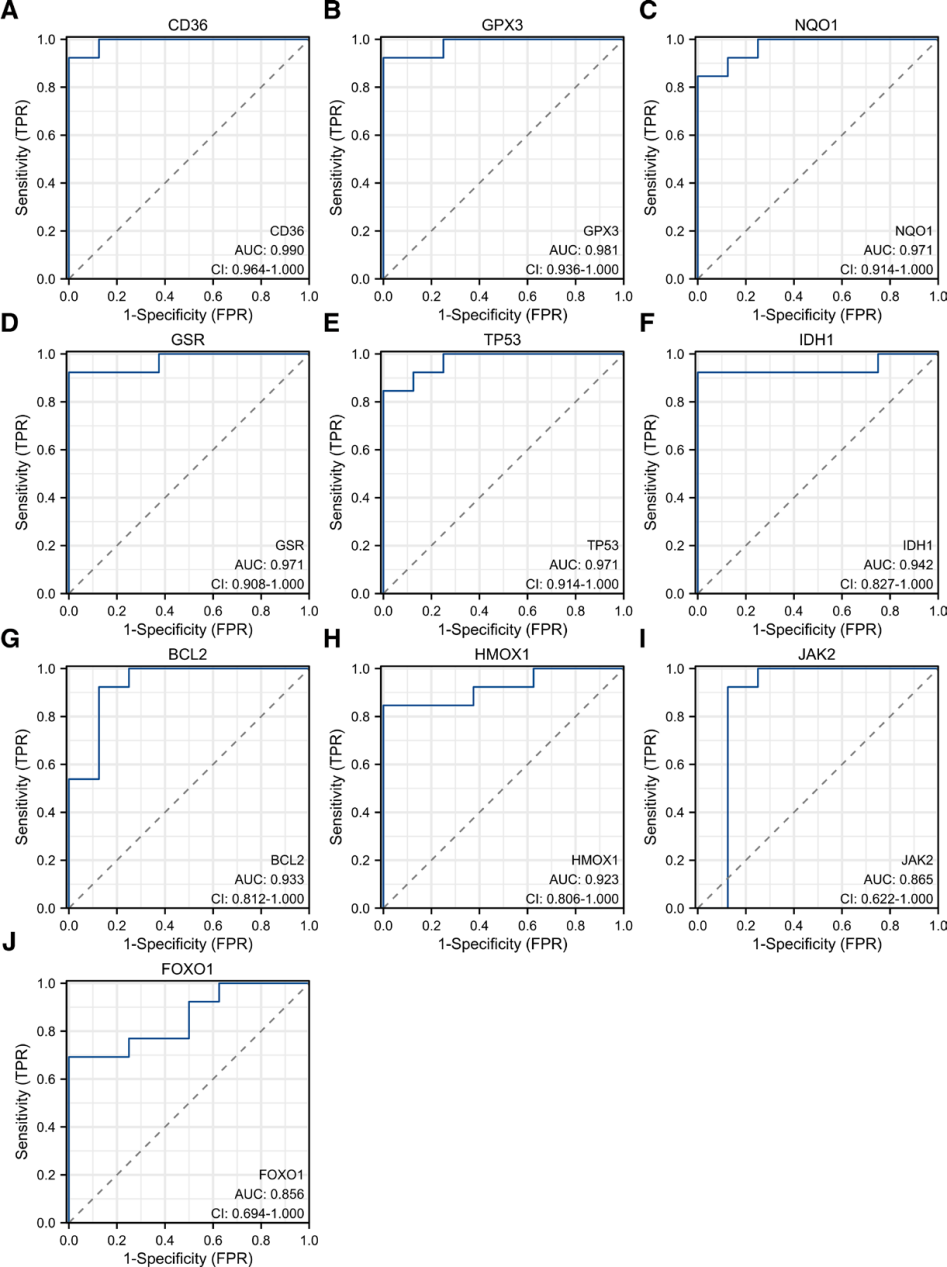
Diagnostic value of 10 hub genes in the GSE13205 database. ROC curves of CD36 (A), GPX3 (B), NQO1 (C), GSR (D), TP53 (E), IDH1 (F), BCL2 (G), HMOX1 (H), JAK2 (I), and FOXO1 (J). ROC = receiver operating characteristic.

**Figure 9.**
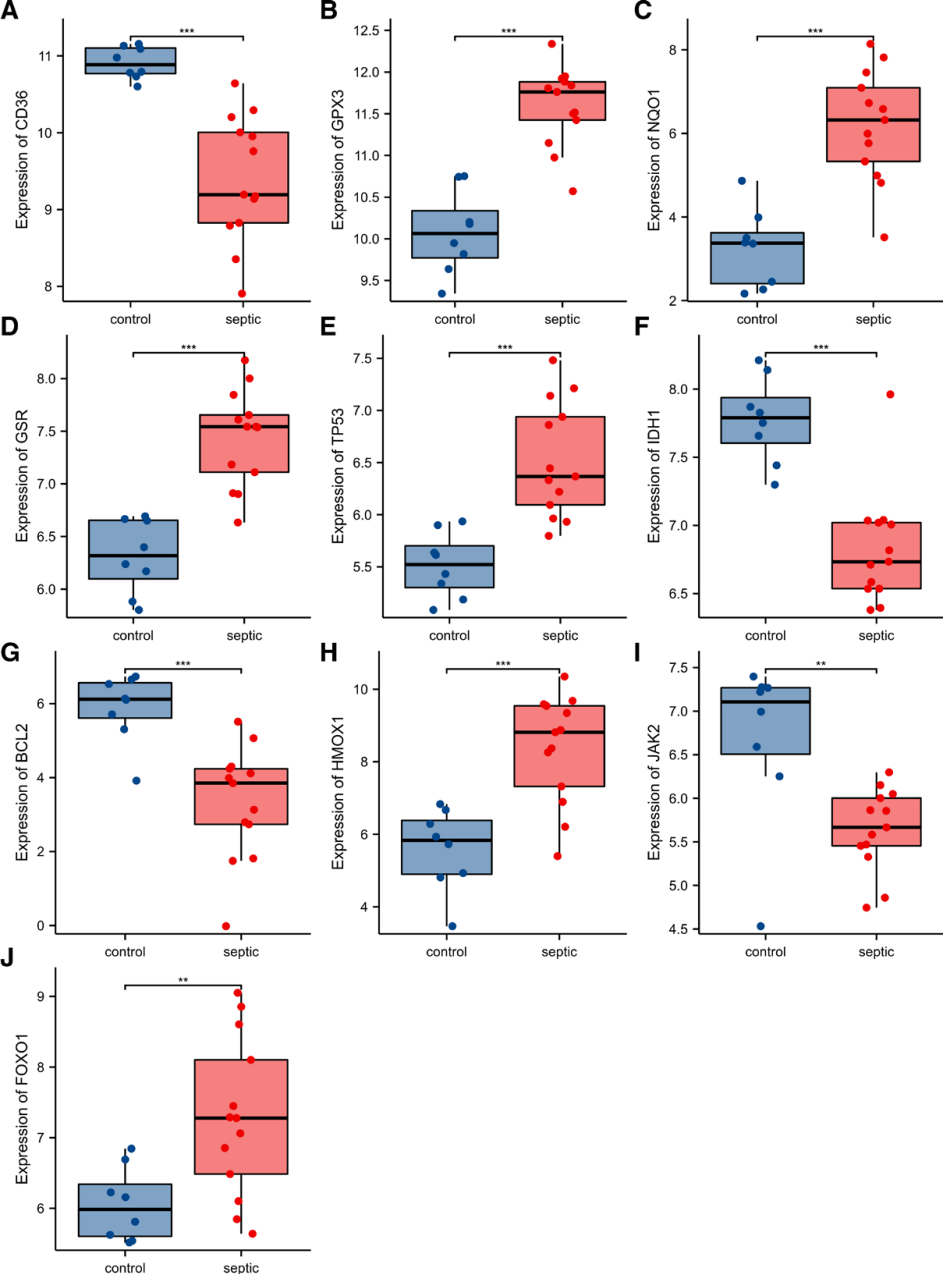
Comparison of the expression of 10 hub genes in the GSE13205 database. Expression levels of CD36 (A), GPX3 (B), NQO1 (C), GSR (D), TP53 (E), IDH1 (F), BCL2 (G), HMOX1 (H), JAK2 (I), and FOXO1 (J). **P < .01; ***P < .001.

Receiver operating characteristic curves were used to evaluate the diagnostic value of 10 hub genes in SIM. Figure 8A–J show the diagnostic values of the 10 hub genes. The results showed that the AUCs of all hub genes were greater than 0.850, among which CD36 (AUC, 0.990) had the highest diagnostic value, followed by GPX3 (AUC, 0.981). And the gene expression level and diagnostic value were further verified. Figure 9A–J show the gene expression levels of the 10 hub genes. The results showed that the gene expression levels between the SIM group and the control group were statistically significant. Finally, we screened the top 5 hub genes based on their AUCs as diagnostic genes: CD36, GPX3, NQO1, GSR, and TP53.

### 3.5. Construction of the miRNA-gene network

**Figure 10.**
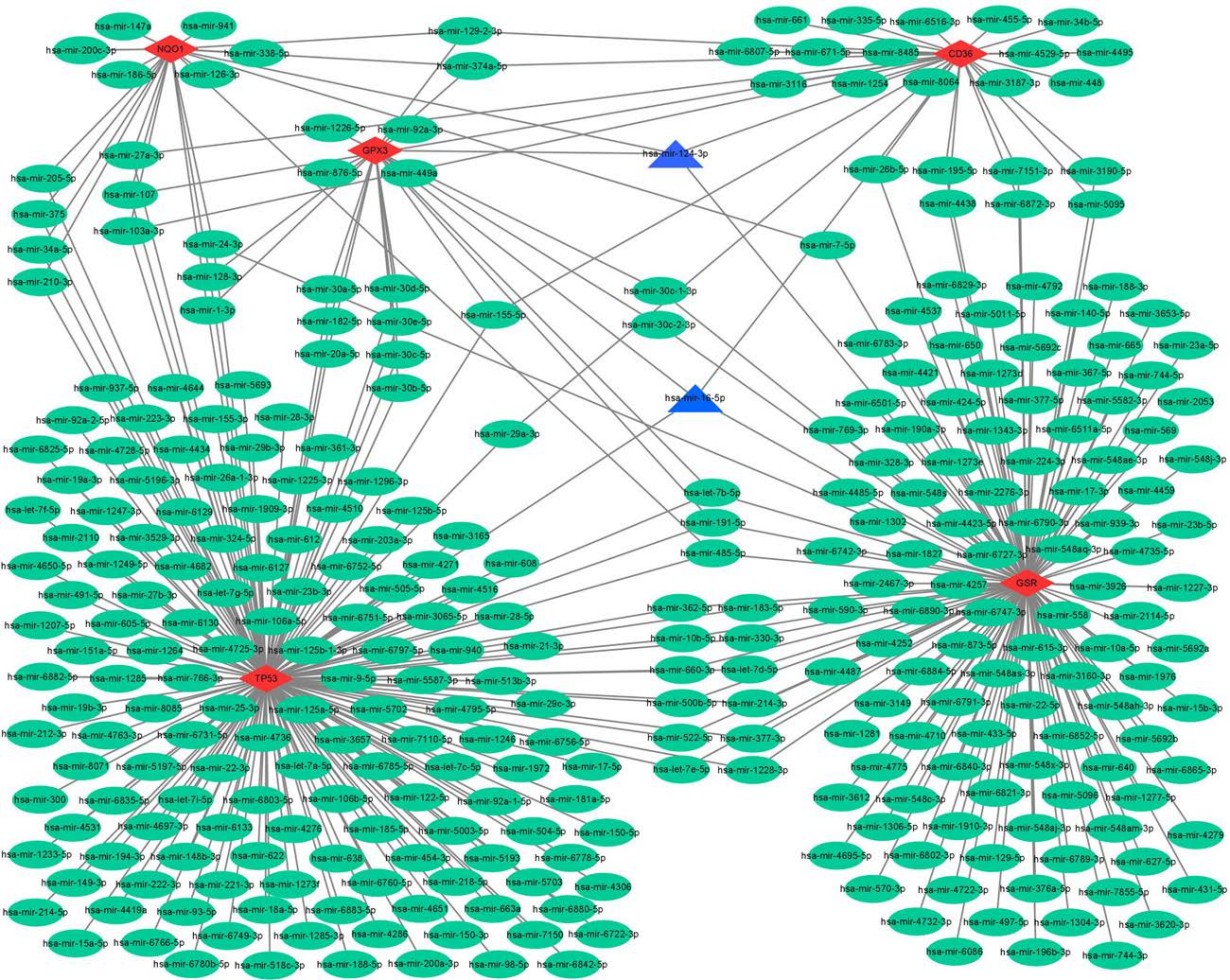
Coexpression network of diagnostic genes and target miRNAs. Red diamonds represent diagnostic genes, and green ellipses represent target miRNAs.

The miRNet database was used to predict target microRNAs (miRNAs) of the diagnostic genes (CD36, GPX3, NQO1, GSR, and TP53). Ultimately, we obtained 319 miRNAs of 5 diagnostic genes (Table S3, Supplemental Digital Content, http://links.lww.com/MD/M269). CD36 was regulated by 31 miRNAs, GPX3 was regulated by 22 miRNAs, NQO1 was regulated by 21 miRNAs, GSR was regulated by 135 miRNAs and TP53 was regulated by 174 miRNAs. And two of these miRNAs regulate 4 genes simultaneously, the hsa-mir-124-3p regulate CD36, GPX3, NQO1, GSR and hsa-mir-16-5p regulate CD36, GPX3, GSR, and TP53. The miRNA-gene network, which comprised 324 nodes and 383 edges, was constructed using Cytoscape (Fig. 10).

## 4. Discussion

Sepsis constitutes a substantial share of mortality in the ICU, therefore emerging as a noteworthy public health concern. Presently, due to the prompt identification of sepsis and advancements in the timely management of infection, fluid resuscitation, life support, and ancillary therapies, there has been a decline in patient mortality associated with sepsis.^[5]^ As the number of sepsis survivors increases, so does one of the most common sequelae SIM. This condition is characterized by pronounced debilitation of the extremities or respiratory muscles that manifest throughout the patient’s stay in the ICU, which cannot be explained by causes other than severe sepsis. Multiple investigations have substantiated that oxidative stress plays an important role in skeletal muscle dysfunction in sepsis.^[12–14]^ The search for efficient biomarkers representing oxidative stress has attracted widespread attention, but the diagnostic biomarkers related to oxidative stress in SIM remain unexplored. Therefore, this study focused on using bioinformatics to screen oxidative stress-related diagnostic biomarkers in muscle tissue of SIM patients.

Recently, several studies have focused on the analysis of DEGs in SIM patients. For example, Ning et al screened and identified key biomarkers in patients with SIM.^[15]^ However, bioinformatic analysis of OS-genes in SIM has not yet been performed. In the present study, 1089 DEGs and 453 OS-genes were used to identify 25 OS-DEGs, including 15 upregulated genes and 10 downregulated genes (Table 1). Five diagnostic biomarkers were identified: CD36, GPX3, NQO1, GSR, and TP53.

The CD36 gene encodes the protein that plays a pivotal part in the metabolic processes and energy generation. The process encompasses the transportation of fatty acids, lipoproteins, and vitamins. CD36 is expressed on the plasma membrane of various cells, encompassing myocytes, adipocytes, and immune cells. Study has demonstrated that variations in the CD36 gene can exert a significant influence on an individual’s susceptibility to cardiovascular illnesses,^[16]^ type 2 diabetes,^[17]^ and metabolic disorders.^[18]^ Additionally, research has revealed that the signaling of oxidized LDL/CD36 in macrophages establishes a connection between dysregulated fatty acid metabolism and oxidative stress from the mitochondria. This process ultimately leads to the development of chronic inflammation.^[19]^

The GPX3 gene encodes the enzyme glutathione peroxidase 3, which belongs to the glutathione peroxidase family and is involved in hydrogen peroxide detoxification. GPx3, a highly conserved protein and a prominent ROS scavenger in plasma, has been widely investigated and proved to have an important function as a tumor suppressor in the majority of malignancies.^[20]^ Glutathione is a thiol donor that can be used to reduce harmful substances such as hydrogen peroxide (H202) and lipid peroxide produced during human metabolism, regulate the cellular microenvironment, and maintain the structural integrity as well as normal functions of cells.^[21]^ Simultaneously, inflammatory reactions can be decreased, and GPXs can have anti-inflammatory effects through modulating ROS.^[21]^ Furthermore, investigations have demonstrated that GPx3 concentrations and bioactivity are significantly decreased in sepsis patients.^[22]^

The NQO1 gene encodes the enzyme nicotinamide adenine dinucleotide phosphate dehydrogenase quinone 1. Previous research has revealed that NQO1 is a downstream gene of nuclear factor erythroid 2-related factor 2 (NRF2), which is involved in inflammation suppression.^[23]^ Furthermore, over-expression of NQO1 and HMOX1 alone or in combination has been shown to suppress the production of pro-inflammatory molecules such as TNF-a and IL-1.^[24]^ In addition, NQO1 may be involved in the regeneration of antioxidant forms of α-tocopherol (vitamin E) following free radical damage.^[25]^ And in an important observation for NQO1’s possible function in oxidative stress, NQO1 was demonstrated to convert CoQ substrates with varying chain lengths to their antioxidant hydroquinone forms. This discovery demonstrated that NQO1 integrated hydroquinone forms of long chain CoQ derivatives CoQ9 and CoQ10 into lipid membranes, providing protection from lipid peroxidation. The author proposed that NQO1 evolved as an endogenous CoQ reductase to maintain antioxidant protection.^[26]^

The GSR gene codes for a pyridine nucleotide-disulfide oxidoreductase of class I. This enzyme is a flavoprotein homodimer. It is a key enzyme in cellular antioxidant defense, converting oxidized glutathione disulfide to the sulfhydryl form Glutathione, which is an essential enzymatic antioxidant implicated in mitochondrial ROS regulation.^[27]^ Moreover, studies have revealed that GSR was substantially connected with numerous immune cells and could lower airway inflammation, lung injury, and cell death in mouse influenza model.^[28]^

The TP53 gene encodes the p53 protein, which functions as a tumor suppressor protein and plays a crucial role in the regulation of several signaling pathways. The cellular function of p53 is contingent upon the type of the oxidative stress exerted, as well as the magnitude and duration of that stress.^[29]^ In instances of moderate oxidative stress, TP53 plays a role in facilitating adaptation by activating genes responsible for antioxidant activity. However, in situations of greater severity, TP53 regulates apoptosis by promoting the generation of ROS.^[30]^ The augmentation of nicotinamide adenine dinucleotide phosphate synthesis in cells is influenced by TP53, which occurs as a result of the upregulation of TP53-induced glycolysis and apoptosis regulator.^[31]^ And previous studies have shown that the activation of p53 is necessary for the suppression of myogenic differentiation via cytokine-mediated mechanisms, namely through TNF-α-induced apoptotic amplification. This process ultimately contributes to muscle atrophy, with the extent of oxidative stress exposure duration and intensity playing a significant role.^[32]^ Furthermore, another significant and often encountered factor contributing to muscle atrophy is the state of immobility. The upregulation of p53 during immobility enables it to serve as a significant mediator of muscle atrophy, independent of ATF-4. This results in the activation of p21 and consequent tissue atrophy in all kinds of muscle fibers via cell cycle-dependent mechanisms.^[33]^

These data suggest the importance of these 5 diagnostic gene in inflammatory response or oxidative stress. Our study showed that the OS-genes CD36, GPX3, NQO1, GSR, and TP53 could be used as a biomarker for the diagnosis of SIM.

The miR-124-3p is a kind of microRNA that is involved in various biological processes, including brain development, neural differentiation, and neuronal function. MiR-124-3p also plays a role in gene expression regulation by binding to the target messenger RNA (mRNA) and causing its destruction or inhibition of translation. Inhibiting miR-124-3p expression has been reported in studies to reduce inflammatory response and oxidative stress in AMI rats.^[34]^ In human cardiac myocytes, the miR-124-3p/TRAF6 axis can regulate the inflammatory response and apoptosis generated by ischemia-reperfusion damage.^[35]^ In addition, researches have indicated that miR-124-3p suppresses the activation of the NLRP3 inflammasome via its interaction with TRAF6, therefore effectively reducing microglial secondary inflammation after intracerebral hemorrhage in the basal ganglia.^[36]^ The findings demonstrated that the overexpression of miR-124-3p had a significant impact on reducing the levels of inflammatory factors and apoptosis rate, while promoting cell activity.^[36]^ Given its roles, miR-124-3p emerges as a crucial mediator in the pathology of inflammation-related conditions.

MiR-16-5p is also classified within the microRNA family. Extensive research has been conducted on miR-16-5p, revealing its involvement in several biological processes such as cell proliferation, apoptosis, and tumorigenesis. And it has been suggested that this factor may have a role in the development of several illnesses, such as cancer and cardiovascular disease.^[37,38]^ Moreover, research have shown that the atypical expression of miR-16-5p is associated with oxidative stress and inflammatory response in many pathological conditions. Previous studies have provided evidence that miR-16-5p plays a role in the promotion of endoplasmic reticulum (ER) stress and oxidative stress in cardiac cells by regulating ATF6. This suggests that inhibiting miR-16-5p could be a viable therapeutic strategy for safeguarding the heart against injury caused by ER and oxidative stress.^[38]^ And it was also shown in vitro investigations that miR-16-5p facilitates apoptosis, autophagy, and inflammation in human ventricular myocytes under conditions of ER stress.^[39]^ In addition, there is evidence suggesting that miR-16-5p is associated with protective mechanisms against lung injury after infection. In cellular models exposed to lipopolysaccharide-induced injury, the overexpression of miR-16-5p had a mitigating effect on acute lung damage through inhibition of the systemic inflammatory response via the suppression of TNF-α and interleukin-6.^[40]^ Another investigation provided evidence that the long non-coding RNA X inactive specific transcript has the potential to mitigate sepsis-induced acute lung damage by targeting miR-16-5p.^[41]^ Furthermore, research has shown that the overexpression of miR-16-5p effectively inhibits the inflammatory response via the repression of PIK3R1-mediated activation of the PI3K/Akt/NF-κB pathway to exerting anti-inflammatory effect.^[42]^ All of these data suggest the importance of miR-16-5p in oxidative stress and inflammatory response.

These findings suggest that miR-124-3p and miR-16-5p might be promising targets for the treatment of SIM. However, the effect and possible mechanism of these predicted miRNAs in SIM is unknown, and further exploration is needed to uncover the link between the miRNAs and SIM pathogenesis in order to give theoretical evidence for clinical therapy.

There are some limitations in our research that should be noted. First, our research only selected OS-genes from the GO database. In fact, there should be more OS-genes that have not been identified. Second, our sample sizes were relatively small, although we performed similar internal and external validations to compensate for this limitation. Finally, although the 5 diagnostic genes we screened have been reported to be associated with oxidative stress or inflammatory response, there are insufficient studies to confirm the role of oxidative stress or inflammatory response regulated by these genes in SIM. Therefore, we propose additional in vitro and in vivo studies. Future research could involve manipulating levels of these miRNAs in muscle cell cultures to observe effects on oxidative stress and inflammation. Animal models with altered miRNA expression followed by sepsis induction could help elucidate their roles in SIM development, offering crucial insights for clinical applications.

In conclusion, we identified and validated 5 OS-genes (CD36, GPX3, NQO1, GSR, and TP53) as novel diagnostic biomarkers in the early stages of SIM. These genes may participate in the occurrence and development of SIM through the inflammatory response or immune regulation. Our findings may provide potential targets for the treatment of SIM.

## Acknowledgments

We acknowledge and GEO database for providing their platforms and contributors for uploading their meaningful datasets.

## Author contributions

**Conceptualization:** Jun Zhou.

**Data curation:** Xiang Li, JinXia Yan.

**Formal analysis:** Xiang Li, WeiXing Ge

**Funding acquisition:** Dan Huang, Jun Zhou, Ning Zhang

**Investigation:** Ning Zhang, Qi Yan

**Methodology:** Qi Yan

**Resources:** WeiXing Ge

**Software:** Xiang Li

**Supervision:** JinXia Yan, Jun Zhou

**Validation:** JinXia Yan, Qi Yan

**Visualization:** Qi Yan

**Writing – original draft:** Ning Zhang, Dan Huang

**Writing – review & editing:** Dan Huang, WeiXing Ge, Jun Zhou

## Supplementary Material



**Figure:**
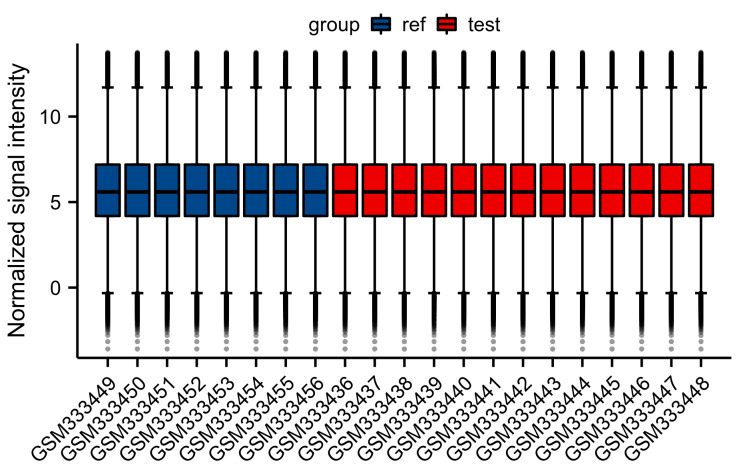


**Figure:**