# Supplementary material for: Identification and validation of oxidative stress-related genes in sepsis-induced myopathy
Source: Medicine (Baltimore). 2024 May 3;103(18):e37933. doi: 10.1097/MD.0000000000037933 (PMC11062695; doi:10.1097/MD.0000000000037933)
Supplement: Supplementary file 1 [file medi-103-e37933-s001.docx]

| Supplementary table 1. 543 oxidative stress-related genes. | | | | | | |
| --- | --- | --- | --- | --- | --- | --- |
| **Gene** | MAPK7 | SLC25A23 | SELENOS | NQO1 | PARP1 | TP53 |
| OSER1 | PRODH | ERCC8 | TOP2B | TXN | HSF1 | MT-ND4 |
| OSGIN1 | MAP3K5 | PINK1 | MAPK9 | RHOB | AQP1 | MT-ATP6 |
| OSGIN2 | NET1 | ROMO1 | PENK | ZNF580 | SDHD | IL1A |
| ALKBH2 | SUMO4 | MPO | ALOX5 | ATP13A2 | SDC1 | BAK1 |
| GPX5 | ATF4 | PRR5L | MMP2 | URS0000476BE1_9606 | PRDX6 | PTK2B |
| OXR1 | FANCC | CPEB2 | AIFM1 | URS0000324096_9606 | DHCR24 | PTGS2 |
| TLDC2 | NUDT2 | GPR37L1 | PPARGC1A | URS00005743AE_9606 | HBB | APOD |
| GPX7 | PEX13 | PEX5 | PARK7 | STC2 | RNF112 | WRN |
| GPX8 | ALDH3B1 | PRDX5 | SIRPA | PDLIM1 | FYN | ERCC2 |
| PYROXD1 | BRF2 | PCGF2 | ERN1 | PPP2CB | S100A7 | SCGB1A1 |
| GPX6 | TMIGD1 | PEX2 | BMAL1 | STOX1 | TXNIP | SP1 |
| PYCR1 | MGAT3 | LONP1 | MT-ND6 | SIN3A | IDH1 | GCLM |
| MEAK7 | PEX10 | PEX12 | MT-ND5 | WNT1 | AREG | ALS2 |
| ALKBH3 | MAP1LC3A | PLEKHA1 | MT-ND3 | NOL3 | AK4 | CAPN2 |
| NCOA7 | MPV17L | GPR37 | TRAP1 | URS000055128B_9606 | MTF1 | MDM2 |
| ERMP1 | STX2 | PTPRK | NDUFA6 | UBQLN1 | PTPRN | ATM |
| TBC1D24 | MPV17L2 | PDK2 | BAG5 | FOXO1 | NME5 | PKD2 |
| GPX2 | PJVK | GLRX2 | NDUFA12 | URS00003768C5_9606 | ATF2 | EGFR |
| IPCEF1 | RWDD1 | TP53INP1 | NDUFS2 | ABCD1 | MAPT | AKT1 |
| VKORC1L1 | ALKBH5 | CAMKK2 | NDUFB4 | MAPK8 | TACR1 | SIRT2 |
| PYCR2 | ALKBH1 | FABP1 | NDUFS8 | FOS | XRCC1 | UCN |
| SRXN1 | NOX1 | PRKRA | PNKP | PCNA | OGG1 | PLK3 |
| ZNF277 | MAPK13 | KEAP1 | ERO1A | MT3 | CRYAB | CASP3 |
| IL18BP | AIFM2 | GJB2 | ABCC1 | PRKAA2 | FOXO4 | SNCA |
| MSRA | MAP2K4 | PEX14 | PRKN | KRT1 | MACROH2A1 | GATA4 |
| CRYGD | PPIF | EPAS1 | STAU2 | RRM2B | CRK | RELA |
| GSR | UCP1 | PPP1R15B | GCH1 | DUOX2 | GSTP1 | HYAL1 |
| cat_human | PXN | NCF1 | DHFR | DUOX1 | RPS3 | ALAD |
| FBLN5 | PDCD10 | JUN | PRDX2 | MICB | KAT2B | ABCB11 |
| SLC25A14 | SLC7A11 | DNAJC15 | FZD1 | MBL2 | EZH2 | URS000039ED8D_9606 |
| RAD52 | ZNF622 | DAXX | FBXO7 | CHRNA4 | NOS3 | ANXA1 |
| ANKRD2 | STK25 | SESN2 | MELK | STOML2 | ERCC6 | BMP7 |
| ANKZF1 | ARL6IP5 | FUT8 | HDAC6 | PRDX4 | IL10 | TNFAIP3 |
| GGT7 | VNN1 | MAPKAP1 | FBXW7 | ERCC3 | TOR1A | PRKAA1 |
| RBM11 | CBX8 | PNPLA8 | RCAN1 | BANF1 | ADAM9 | GCLC |
| MGST1 | PXDNL | TMEM135 | EPX | RBPMS | BID | LRRK2 |
| NME8 | DGKK | STK26 | COA8 | NEIL1 | RGS14 | URS00004C9052_9606 |
| SLC25A24 | PSMB5 | SESN3 | CYGB | NAGLU | P4HB | URS000024463E_9606 |
| CCS | RLIG1 | SESN1 | PXDN | NR4A2 | FOSL1 | PAX2 |
| ERCC6L2 | SELENOP | THG1L | GPX4 | MMP9 | UCP2 | ADIPOQ |
| DHRS2 | ANGPTL7 | NFE2L2 | NAPRT | CHUK | HSPB1 | INS |
| VRK2 | TXNRD2 | SGK2 | HAO1 | URS000030BD69_9606 | URS000006FDD4_9606 | TWIST1 |
| SOD3 | PSIP1 | GSS | HP | SPHK1 | VCP | RHOA |
| MPV17 | CYB5B | TXN2 | LIAS | RIPK1 | SLC1A1 | APP |
| ADPRS | MSRB2 | HBA1 | SLC23A2 | MAPK3 | PDGFRA | CTNNB1 |
| AGAP3 | TPO | HMOX2 | SOD1 | GSKIP | RACK1 | SHMT2 |
| URS00000DF6D0_9606 | NONO | ETFDH | PTGS1 | SHPK | HSPA1B | FKBP1B |
| URS0000CCE0E6_9606 | PPIA | TAT | SFPQ | DIABLO | TREX1 | TREM2 |
| WNT16 | FANCD2 | LPO | PRDX3 | HIF1A | BTK | ABL1 |
| SELENON | MMP3 | HTRA2 | SETX | TRIM25 | ATP7A | CD38 |
| PRDX1 | STAU1 | KLF2 | PNPT1 | SELENOK | ERCC1 | MMP14 |
| MSRB3 | TRPA1 | SOD2 | DAPK1 | CYP1B1 | SIRT1 | KCNA5 |
| ATOX1 | STK24 | MT-RNR2 | AIF1 | STX4 | MAPK1 | COL1A1 |
| C19orf12 | IL18RAP | G6PD | GATA5 | SMPD3 | HSPA1A | SRC |
| SCARA3 | EIF2S1 | FXN | CAT | BNIP3 | ZC3H12A | BCL2 |
| GPX3 | GPX1 | ATP2A2 | ETV5 | APOA4 | PRKCD | PML |
| TMEM161A | ARNT | TPM1 | PPARGC1B | SQSTM1 | EDNRA | PPARD |
| SLC4A11 | URS0000028BB8_9606 | URS00005B3525_9606 | TRPM2 | FER | CDK1 | TNF |
| CHCHD2 | URS00004E5112_9606 | NUDT15 | ENDOG | NUPR1 | HDAC2 | HYAL2 |
| PLA2R1 | URS00006054DA_9606 | PDK1 | ADCYAP1R1 | FOXO3 | PRKD1 | STAT1 |
| MCL1 | URS0000812128_9606 | NUDT1 | PDGFD | MYB | AXL | URS0000338542_9606 |
| MCTP1 | UCP3 | OXSR1 | ECT2 | RIPK3 | STAT6 | EDN1 |
| FTO | MT-CO1 | HMOX1 | KDM6B | CD36 | BECN1 | IL6 |
| MSH2 | FGF8 | PRNP | JAK2 | APOE | PSEN1 |  |
