# Supplementary material for: Identification and validation of oxidative stress-related genes in sepsis-induced myopathy
Source: Medicine (Baltimore). 2024 May 3;103(18):e37933. doi: 10.1097/MD.0000000000037933 (PMC11062695; doi:10.1097/MD.0000000000037933)
Supplement: Supplementary file 2 [file medi-103-e37933-s004.docx]

| Supplementary Table 2. Significantly up- and downregulated genes | | | | | | | |
| --- | --- | --- | --- | --- | --- | --- | --- |
| id | logFC | AveExpr | t | P.Value | adj.P.Val | B | Gene.Symbol |
| 206349_at | -5.347831677 | 3.236549165 | -9.024470608 | 9.46936E-09 | 5.16911E-06 | 10.26295212 | LGI1 |
| 226228_at | -4.701293378 | 7.907429793 | -7.52481692 | 1.8983E-07 | 3.50666E-05 | 7.372838682 | AQP4 |
| 244572_at | -4.67950718 | 5.6891535 | -9.93250466 | 1.77388E-09 | 1.79831E-06 | 11.85724133 | KY |
| 219464_at | -4.390082825 | 3.953801966 | -8.431611927 | 2.98992E-08 | 1.01037E-05 | 9.159386155 | CA14 |
| 239203_at | -4.384216792 | 6.678405621 | -8.84065383 | 1.34595E-08 | 6.28382E-06 | 9.926179365 | LSMEM1 |
| 231391_at | -4.344354895 | 3.855597431 | -6.394194353 | 2.21696E-06 | 0.000175986 | 4.980234214 | CTXN3 |
| 1559429_a_at | -4.238360997 | 6.550706542 | -10.29233718 | 9.3894E-10 | 1.18984E-06 | 12.45805872 | C3orf43 |
| 201310_s_at | -4.08505098 | 7.568215271 | -16.56713553 | 1.06486E-13 | 1.25944E-09 | 20.62410475 | NREP |
| 1563462_at | -3.862271881 | 5.245359188 | -5.233735397 | 3.24559E-05 | 0.00106658 | 2.358420447 | LOC285419 |
| 237326_at | -3.848014799 | 3.619752454 | -8.15309713 | 5.21361E-08 | 1.46817E-05 | 8.623417353 | LINC00310 |
| 202965_s_at | -3.846339536 | 3.052641745 | -8.875396229 | 1.25899E-08 | 6.11938E-06 | 9.990200288 | CAPN6 |
| 227401_at | -3.79938314 | 8.221349258 | -7.333295229 | 2.84458E-07 | 4.47361E-05 | 6.980206842 | IL17D |
| 203153_at | -3.708026355 | 6.63951122 | -11.13914263 | 2.22683E-10 | 6.25109E-07 | 13.80610169 | IFIT1 |
| 1564807_at | -3.620976831 | 4.68579845 | -5.84234689 | 7.80112E-06 | 0.000407752 | 3.751333348 | RP11-504A18.1 |
| 241961_at | -3.559784216 | 5.308791591 | -4.507832834 | 0.000184537 | 0.003599639 | 0.664375379 | TECRL |
| 1560750_at | -3.479918781 | 6.364598775 | -5.101409863 | 4.44447E-05 | 0.001319653 | 2.051503284 | LOC151121 |
| 207819_s_at | -3.475353958 | 4.681752879 | -5.317478424 | 2.66201E-05 | 0.00092966 | 2.55201012 | ABCB4 |
| 206844_at | -3.458819607 | 8.585419222 | -5.990612635 | 5.54424E-06 | 0.000316781 | 4.085031097 | FBP2 |
| 205553_s_at | -3.409400912 | 10.42702053 | -4.345450751 | 0.000273055 | 0.004730732 | 0.283789324 | CSRP3 |
| 218574_s_at | -3.389020208 | 8.932377294 | -7.122264718 | 4.46713E-07 | 6.26492E-05 | 6.541478335 | LMCD1 |
| 230915_at | -3.355253465 | 8.034486227 | -6.518811332 | 1.67718E-06 | 0.000147066 | 5.252523417 | DHRS7C |
| 205659_at | -3.346179596 | 5.585686942 | -5.129625588 | 4.15584E-05 | 0.001257016 | 2.117044782 | HDAC9 |
| 206891_at | -3.327567215 | 9.59432948 | -2.989433004 | 0.006884751 | 0.045328329 | -2.807576345 | ACTN3 |
| 1553593_a_at | -3.236632906 | 4.454251628 | -9.222585448 | 6.51367E-09 | 4.0547E-06 | 10.62055959 | TAL2 |
| 220014_at | -3.217907997 | 5.187185835 | -5.515359967 | 1.67057E-05 | 0.000675887 | 3.007196373 | PRR16 |
| 237222_at | -3.217641842 | 8.1758873 | -5.928056278 | 6.4016E-06 | 0.000351641 | 3.944537169 | FSD2 |
| 1556351_at | -3.216529103 | 2.29141236 | -4.45059514 | 0.000211856 | 0.003913248 | 0.530203065 | HCN1 |
| 204685_s_at | -3.188562111 | 6.934854512 | -9.47575231 | 4.06716E-09 | 3.07045E-06 | 11.06953393 | ATP2B2 |
| 226145_s_at | -3.185717739 | 5.079032109 | -7.040928395 | 5.32429E-07 | 7.07903E-05 | 6.370690421 | FRAS1 |
| 213955_at | -3.009705223 | 8.473966573 | -5.632305371 | 1.2707E-05 | 0.000555941 | 3.274556318 | MYOZ3 |
| 1553444_a_at | -2.979517577 | 4.460482211 | -4.877998938 | 7.57802E-05 | 0.001952674 | 1.530954669 | C1orf127 |
| 220244_at | -2.929261381 | 7.12731985 | -10.79942778 | 3.92876E-10 | 6.43456E-07 | 13.27615883 | LINC00312 |
| 220116_at | -2.922044962 | 4.735074226 | -5.31398657 | 2.68407E-05 | 0.000932774 | 2.543948678 | KCNN2 |
| 1558645_at | -2.913818591 | 6.551600514 | -8.754815997 | 1.5885E-08 | 7.04484E-06 | 9.767262918 | MIR133A1HG |
| 204686_at | -2.888299385 | 7.460642494 | -8.931441865 | 1.13076E-08 | 5.98831E-06 | 10.09311434 | IRS1 |
| 230493_at | -2.875813975 | 6.165668232 | -5.120672382 | 4.24531E-05 | 0.001277626 | 2.096253112 | SHISA2 |
| 232384_s_at | -2.87495908 | 1.989601672 | -5.224741449 | 3.31554E-05 | 0.001080008 | 2.337598036 | LOC101927272 |
| 214862_x_at | -2.872730723 | 3.307716379 | -4.899490284 | 7.19785E-05 | 0.001879281 | 1.581138067 | RP11-119F7.5 |
| 236860_at | -2.798443662 | 5.479413168 | -9.632805737 | 3.04886E-09 | 2.46748E-06 | 11.34359637 | NPY6R |
| 222572_at | -2.781842546 | 8.952754198 | -8.579984462 | 2.2327E-08 | 8.51835E-06 | 9.440314671 | PDP1 |
| 237024_at | -2.762868155 | 4.180230759 | -6.016842142 | 5.22055E-06 | 0.000303168 | 4.14380623 | LSMEM2 |
| 1563641_a_at | -2.759514832 | 6.37407048 | -6.26296023 | 2.98038E-06 | 0.00021087 | 4.691333832 | SNX20 |
| 1564679_at | -2.723516564 | 4.486978482 | -4.862377272 | 7.86702E-05 | 0.002009631 | 1.494464906 | ASB15 |
| 226492_at | -2.720870334 | 6.876116313 | -5.817875858 | 8.25539E-06 | 0.000425134 | 3.696023928 | SEMA6D |
| 226140_s_at | -2.714427964 | 8.159651493 | -7.77147215 | 1.13579E-07 | 2.57915E-05 | 7.870662181 | OTUD1 |
| 233949_s_at | -2.693289474 | 6.621652744 | -7.176870149 | 3.97248E-07 | 5.80048E-05 | 6.655611468 | MYH7B |
| 230331_at | -2.68149974 | 3.350679658 | -3.161738602 | 0.004620825 | 0.034278926 | -2.432032979 | METTL21C |
| 205736_at | -2.672041926 | 10.18513607 | -6.069345822 | 4.62938E-06 | 0.000279829 | 4.2612164 | PGAM2 |
| 201744_s_at | -2.67001907 | 6.9966439 | -6.840299528 | 8.23997E-07 | 9.22149E-05 | 5.945446465 | LUM |
| 214321_at | -2.667378104 | 3.922180068 | -5.378494111 | 2.30491E-05 | 0.000837938 | 2.692716332 | NOV |
| 230671_at | -2.644994373 | 7.231381942 | -5.100552447 | 4.45355E-05 | 0.001320586 | 2.049510833 | CTD-2083E4.7 |
| 213749_at | -2.627665958 | 4.694440077 | -4.693373806 | 0.000118038 | 0.002630796 | 1.099139124 | MASP1 |
| 212915_at | -2.621230372 | 7.877568106 | -6.943024368 | 6.58458E-07 | 8.07171E-05 | 6.163878923 | PDZRN3 |
| 1570289_at | -2.618372758 | 4.221902663 | -5.55163681 | 1.53442E-05 | 0.000631603 | 3.090270263 | LOC646736 |
| 237244_at | -2.55723674 | 6.814760825 | -6.910144496 | 7.07355E-07 | 8.39411E-05 | 6.09412314 | C10orf71-AS1 |
| 232892_at | -2.552011197 | 8.574517289 | -3.885176643 | 0.000828806 | 0.010250158 | -0.790262174 | C20orf166 |
| 222649_at | -2.539221098 | 8.10277785 | -10.79209123 | 3.97777E-10 | 6.43456E-07 | 13.26455584 | XPO4 |
| 229778_at | -2.523000639 | 5.487779661 | -5.946912496 | 6.12979E-06 | 0.00034144 | 3.986932644 | SPX |
| 230195_at | -2.522436294 | 8.815900333 | -3.693597941 | 0.001312751 | 0.014183622 | -1.23261849 | LINC01405 |
| 206895_at | -2.508447243 | 9.28610343 | -8.780437334 | 1.5117E-08 | 6.78963E-06 | 9.814807654 | PPP1R3A |
| 203823_at | -2.495188103 | 6.491683229 | -6.93869598 | 6.64691E-07 | 8.09036E-05 | 6.154704708 | RGS3 |
| 207901_at | -2.485105274 | 1.670795699 | -3.745415056 | 0.001159474 | 0.013002668 | -1.113369802 | IL12B |
| 219990_at | -2.482822932 | 6.191641018 | -3.577859111 | 0.001730958 | 0.017261904 | -1.497718832 | E2F8 |
| 204051_s_at | -2.481157832 | 4.421339942 | -4.391788897 | 0.000244158 | 0.004338115 | 0.39237023 | SFRP4 |
| 227061_at | -2.474481668 | 4.835751248 | -4.979042325 | 5.95073E-05 | 0.001638043 | 1.766710032 | LINC01279 |
| 231925_at | -2.465708799 | 5.219715473 | -7.990387655 | 7.24906E-08 | 1.85044E-05 | 8.305072246 | RP11-38P22.2 |
| 203766_s_at | -2.455158488 | 6.834707433 | -6.596076941 | 1.41213E-06 | 0.000131165 | 5.420326905 | LMOD1 |
| 225105_at | -2.429263023 | 6.557382331 | -4.25941945 | 0.000336095 | 0.005485427 | 0.082313561 | C12orf75 |
| 242947_at | -2.429073359 | 4.946662823 | -5.705482876 | 1.0715E-05 | 0.00050091 | 3.441178491 | RP4-680D5.8 |
| 214724_at | -2.426242905 | 7.342809699 | -9.374462003 | 4.90578E-09 | 3.34744E-06 | 10.89097318 | DIXDC1 |
| 229580_at | -2.414675332 | 4.237430007 | -4.63177556 | 0.000136896 | 0.002894047 | 0.954856122 | CLSTN2 |
| 215218_s_at | -2.408858898 | 5.256866999 | -5.326060974 | 2.60857E-05 | 0.000913694 | 2.571820078 | WDR62 |
| 205694_at | -2.401250551 | 4.799530342 | -5.315985677 | 2.67142E-05 | 0.000931289 | 2.548564016 | TYRP1 |
| 1553634_a_at | -2.396400974 | 2.371963008 | -5.091622329 | 4.54926E-05 | 0.001343337 | 2.028756437 | WEE2-AS1 |
| 238867_at | -2.35588246 | 8.73791243 | -6.730118223 | 1.04968E-06 | 0.000108 | 5.709541264 | TMEM182 |
| 217525_at | -2.339870625 | 4.140663006 | -3.267426097 | 0.003609023 | 0.02873129 | -2.198037388 | OLFML1 |
| 219892_at | -2.337440659 | 6.996122767 | -6.712127562 | 1.09217E-06 | 0.000109473 | 5.670864643 | TM6SF1 |
| 217623_at | -2.333616512 | 6.851594347 | -4.602011103 | 0.000147067 | 0.003048033 | 0.88511354 | MYLK3 |
| 227657_at | -2.331138353 | 6.291175843 | -10.77935491 | 4.06439E-10 | 6.43456E-07 | 13.24439685 | RNF150 |
| 228906_at | -2.315020763 | 5.319676453 | -10.07320473 | 1.38072E-09 | 1.57901E-06 | 12.09421282 | TET1 |
| 243737_at | -2.309808124 | 8.357751039 | -5.584027255 | 1.42241E-05 | 0.000598229 | 3.164340472 | ATP1B4 |
| 205589_at | -2.301836064 | 10.69592638 | -3.086845334 | 0.005498995 | 0.038784729 | -2.596225254 | MYL3 |
| 1552402_at | -2.299125931 | 5.632508979 | -5.288006986 | 2.85414E-05 | 0.000970278 | 2.483941347 | CALML6 |
| 225747_at | -2.298686131 | 9.435859785 | -7.054326941 | 5.17223E-07 | 6.95156E-05 | 6.398888623 | COQ10A |
| 204797_s_at | -2.285736511 | 6.6319932 | -4.986516625 | 5.84542E-05 | 0.001615062 | 1.784129276 | EML1 |
| 204135_at | -2.274566675 | 8.43235874 | -7.19630439 | 3.81035E-07 | 5.60991E-05 | 6.69612945 | FILIP1L |
| 209596_at | -2.268390468 | 4.425739815 | -4.498115829 | 0.000188912 | 0.003639185 | 0.641597423 | MXRA5 |
| 1560646_at | -2.259949311 | 4.088462252 | -4.04736946 | 0.000560721 | 0.007786883 | -0.413129072 | METTL21EP |
| 226930_at | -2.259874422 | 4.375125045 | -4.333630389 | 0.00028096 | 0.004829945 | 0.256097309 | FNDC1 |
| 222304_x_at | -2.258784594 | 6.175720797 | -6.236710492 | 3.16291E-06 | 0.000217493 | 4.633287842 | OR7E47P |
| 1560228_at | -2.251291575 | 4.764053476 | -5.210363495 | 3.43055E-05 | 0.001099575 | 2.304298749 | SNAI3 |
| 205609_at | -2.245130982 | 6.611409628 | -5.745503227 | 9.76335E-06 | 0.000473371 | 3.532073799 | ANGPT1 |
| 204784_s_at | -2.239873536 | 8.188153841 | -7.040699688 | 5.32693E-07 | 7.07903E-05 | 6.370208871 | MLF1 |
| 206717_at | -2.236034062 | 6.874440867 | -4.425914724 | 0.000224855 | 0.004078202 | 0.47235247 | MYH8 |
| 226782_at | -2.234581707 | 7.710568179 | -4.693099136 | 0.000118116 | 0.002630882 | 1.098495938 | SLC25A30 |
| 205968_at | -2.232903524 | 7.537617402 | -4.131845738 | 0.000457324 | 0.006809395 | -0.216008586 | KCNS3 |
| 214608_s_at | -2.22500803 | 5.00661302 | -4.27315451 | 0.000325131 | 0.005353274 | 0.114466799 | EYA1 |
| 210169_at | -2.220186764 | 5.096883279 | -7.025340544 | 5.50701E-07 | 7.26392E-05 | 6.33785285 | SEC14L5 |
| 1559977_a_at | -2.219300096 | 8.48738797 | -4.91119053 | 6.99905E-05 | 0.001845024 | 1.608450159 | SLC25A34 |
| 205898_at | -2.212858171 | 6.041042844 | -6.227505615 | 3.2296E-06 | 0.000219224 | 4.612912907 | CX3CR1 |
| 239398_at | -2.209940982 | 10.80662363 | -9.339314025 | 5.23708E-09 | 3.44115E-06 | 10.82867914 | KLHL31 |
| 203477_at | -2.199600213 | 8.56136179 | -9.792823487 | 2.28016E-09 | 2.07448E-06 | 11.61935997 | COL15A1 |
| 221045_s_at | -2.198753358 | 6.962844544 | -9.983188904 | 1.62034E-09 | 1.69097E-06 | 11.94290986 | PER3 |
| 213272_s_at | -2.190880176 | 7.113568453 | -10.92398962 | 3.18577E-10 | 6.43456E-07 | 13.47213045 | TMEM159 |
| 230367_at | -2.189599338 | 9.769670082 | -4.949859205 | 6.38063E-05 | 0.001732192 | 1.698669895 | SMTNL1 |
| 222930_s_at | -2.174774236 | 6.443261742 | -9.230057888 | 6.42302E-09 | 4.0547E-06 | 10.63393961 | AGMAT |
| 238165_at | -2.17436466 | 3.861069979 | -3.302507471 | 0.003323517 | 0.027228134 | -2.119816344 | PDZRN3-AS1 |
| 211675_s_at | -2.144751203 | 8.009208848 | -11.08560048 | 2.43326E-10 | 6.25109E-07 | 13.72352248 | MDFIC |
| 212713_at | -2.136515163 | 4.456583767 | -3.453542678 | 0.002326351 | 0.021312655 | -1.780243806 | MFAP4 |
| 204364_s_at | -2.110075136 | 8.581523237 | -6.700541212 | 1.12046E-06 | 0.000111402 | 5.645932862 | REEP1 |
| 214844_s_at | -2.105006283 | 8.701825691 | -7.635659912 | 1.50551E-07 | 3.08777E-05 | 7.597647493 | DOK5 |
| 223764_x_at | -2.10265879 | 7.768836555 | -7.010608571 | 5.68561E-07 | 7.38963E-05 | 6.306786869 | NIPSNAP3B |
| 207996_s_at | -2.101089581 | 5.483852866 | -6.395646513 | 2.20973E-06 | 0.000175986 | 4.98341877 | LDLRAD4 |
| 227848_at | -2.099449083 | 10.20247949 | -4.81131924 | 8.89159E-05 | 0.002172806 | 1.37513354 | PEBP4 |
| 203706_s_at | -2.098430769 | 7.544843824 | -3.831656654 | 0.000942639 | 0.011189935 | -0.914225876 | FZD7 |
| 225242_s_at | -2.092111636 | 5.706107676 | -6.182723308 | 3.57515E-06 | 0.00023535 | 4.513638899 | CCDC80 |
| 1554640_at | -2.090530356 | 2.813969906 | -3.337120281 | 0.00306344 | 0.025714925 | -2.042388253 | PALM2 |
| 230467_at | -2.087739671 | 8.474818662 | -5.136456576 | 4.08887E-05 | 0.00124948 | 2.132904632 | TMEM52 |
| 212651_at | -2.075256166 | 8.679331719 | -4.959489944 | 6.23539E-05 | 0.001699264 | 1.721128603 | RHOBTB1 |
| 222954_at | -2.069241809 | 9.387285859 | -5.396025302 | 2.2116E-05 | 0.00081066 | 2.73308816 | FBXO40 |
| 238778_at | -2.055100859 | 6.113712394 | -3.689366262 | 0.001326117 | 0.014301913 | -1.242342541 | MPP7 |
| 215076_s_at | -2.049272336 | 8.079857099 | -6.436286296 | 2.01712E-06 | 0.000165251 | 5.072430899 | COL3A1 |
| 223136_at | -2.039599445 | 5.988922669 | -7.339225912 | 2.80897E-07 | 4.46941E-05 | 6.992444497 | AIG1 |
| 203997_at | -2.030464074 | 8.19656643 | -7.276673316 | 3.2089E-07 | 4.86574E-05 | 6.863116105 | PTPN3 |
| 1556096_s_at | -2.023430393 | 5.983250592 | -4.12236425 | 0.000467911 | 0.006929218 | -0.238152285 | UNC13C |
| 1553311_at | -2.021453163 | 2.308262732 | -3.944095695 | 0.000719207 | 0.009310074 | -0.653497061 | C20orf197 |
| 220432_s_at | -2.018550364 | 2.643561993 | -4.472822255 | 0.000200795 | 0.003767647 | 0.582305662 | CYP39A1 |
| 205073_at | -2.003246079 | 6.705066049 | -5.683928179 | 1.12663E-05 | 0.000516722 | 3.392155083 | CYP2J2 |
| 228101_at | -2.002356285 | 4.053630376 | -4.065080535 | 0.00053727 | 0.007561848 | -0.371835003 | APBA1 |
| 214111_at | -2.000303798 | 2.842514578 | -3.924306443 | 0.000754311 | 0.009634441 | -0.699465129 | OPCML |
| 208096_s_at | -1.988061806 | 4.447865764 | -3.49489451 | 0.002108861 | 0.019884828 | -1.686545614 | COL21A1 |
| 227812_at | -1.981043878 | 3.991074022 | -3.221322415 | 0.004020728 | 0.031000323 | -2.300427177 | TNFRSF19 |
| 226674_at | -1.980893279 | 7.477191341 | -4.712943701 | 0.000112613 | 0.002545044 | 1.144960526 | SHISA4 |
| 208691_at | -1.980087258 | 8.618506019 | -4.762237193 | 0.000100034 | 0.002366269 | 1.260331856 | TFRC |
| 1556499_s_at | -1.977694572 | 7.624858389 | -4.845066881 | 8.2003E-05 | 0.002063159 | 1.45401888 | COL1A1 |
| 213222_at | -1.963349115 | 7.617209875 | -6.633241813 | 1.30034E-06 | 0.000124029 | 5.500757854 | PLCB1 |
| 225763_at | -1.9588089 | 7.822198506 | -7.415149277 | 2.39158E-07 | 4.02171E-05 | 7.148661006 | RCSD1 |
| 219643_at | -1.941823059 | 6.445368726 | -6.936186432 | 6.68333E-07 | 8.09344E-05 | 6.149384411 | LRP1B |
| 213791_at | -1.940753322 | 4.976695665 | -5.159994529 | 3.86636E-05 | 0.001198134 | 2.187530486 | PENK |
| 229116_at | -1.940055164 | 6.767488969 | -4.733441923 | 0.000107199 | 0.002479348 | 1.192944922 | CNKSR2 |
| 1553074_at | -1.939333277 | 6.485592687 | -4.468983762 | 0.000202663 | 0.003794668 | 0.57330774 | ASB11 |
| 1563511_at | -1.933386311 | 4.404650315 | -5.564501486 | 1.48889E-05 | 0.000618605 | 3.119700947 | DKFZp451B082 |
| 219645_at | -1.932190139 | 11.24565314 | -6.01910221 | 5.19358E-06 | 0.000302575 | 4.148866875 | CASQ1 |
| 207089_at | -1.932058996 | 10.17365198 | -4.379082514 | 0.000251762 | 0.004450938 | 0.362593084 | NRAP |
| 219167_at | -1.928904605 | 6.286329846 | -8.90619288 | 1.18677E-08 | 6.09244E-06 | 10.0468062 | RASL12 |
| 213880_at | -1.924523633 | 7.252309883 | -3.503165054 | 0.002067819 | 0.019602016 | -1.667771096 | LGR5 |
| 207927_at | -1.924402312 | 4.260679273 | -4.121454472 | 0.000468939 | 0.006935766 | -0.240276805 | HTR7 |
| 209633_at | -1.920655588 | 10.25139678 | -10.69184395 | 4.71502E-10 | 6.43456E-07 | 13.10533264 | PPP2R3A |
| 226071_at | -1.918895899 | 6.677179499 | -4.898626408 | 7.21276E-05 | 0.001880405 | 1.579121263 | ADAMTSL4 |
| 231036_at | -1.917797821 | 2.857574946 | -3.842611635 | 0.000918143 | 0.010994618 | -0.888874036 | LOC100505774 |
| 203946_s_at | -1.917514939 | 6.419754446 | -3.303271084 | 0.003317554 | 0.027186851 | -2.118110821 | ARG2 |
| 205692_s_at | -1.916076977 | 5.776620493 | -6.406330415 | 2.15734E-06 | 0.000173656 | 5.006840024 | CD38 |
| 206030_at | -1.914031671 | 6.47428332 | -7.503423531 | 1.98555E-07 | 3.55814E-05 | 7.329243938 | ASPA |
| 221599_at | -1.909506414 | 8.931157238 | -8.739228834 | 1.63719E-08 | 7.04484E-06 | 9.738292223 | AAMDC |
| 229114_at | -1.90673546 | 6.573404041 | -6.382878513 | 2.27406E-06 | 0.000177727 | 4.955409542 | GAB1 |
| 219829_at | -1.898334919 | 7.674007902 | -8.059953514 | 6.29352E-08 | 1.679E-05 | 8.441652311 | ITGB1BP2 |
| 206718_at | -1.882774024 | 6.780295252 | -5.133958888 | 4.11323E-05 | 0.001253145 | 2.127105975 | LMO1 |
| 227532_at | -1.88022712 | 10.36900171 | -6.683921901 | 1.16236E-06 | 0.00011393 | 5.610139297 | LRRC39 |
| 221530_s_at | -1.880155494 | 8.725456855 | -11.07743134 | 2.46647E-10 | 6.25109E-07 | 13.71089221 | BHLHE41 |
| 1552732_at | -1.879873198 | 9.881338889 | -6.37467899 | 2.31638E-06 | 0.000179063 | 4.937411198 | ABRA |
| 207302_at | -1.871486743 | 9.699993766 | -5.005072982 | 5.59203E-05 | 0.001562335 | 1.827363078 | SGCG |
| 210038_at | -1.866644861 | 9.850439645 | -6.210195377 | 3.35892E-06 | 0.000224025 | 4.574568517 | PRKCQ |
| 229015_at | -1.860468177 | 6.103062228 | -5.911163742 | 6.65556E-06 | 0.000361597 | 3.906522848 | LOC286367 |
| 1559867_at | -1.859291433 | 9.106098074 | -7.872723924 | 9.22046E-08 | 2.21633E-05 | 8.072452796 | AK056982 |
| 243666_at | -1.857732336 | 1.613201544 | -2.889579017 | 0.008650916 | 0.052785713 | -3.021397486 | CELF4 |
| 226548_at | -1.853503817 | 7.435484107 | -8.005648522 | 7.02734E-08 | 1.82003E-05 | 8.335094697 | SBK1 |
| 212489_at | -1.850320541 | 4.414376292 | -3.905251239 | 0.000789719 | 0.009943514 | -0.743697438 | COL5A1 |
| 212985_at | -1.849000224 | 6.999498435 | -4.842749329 | 8.24598E-05 | 0.002069194 | 1.448602976 | APBB2 |
| 243610_at | -1.847008776 | 2.964278483 | -3.212747073 | 0.004102184 | 0.031430294 | -2.319419051 | C9orf135 |
| 235921_at | -1.836347455 | 6.073238976 | -3.124791068 | 0.005035607 | 0.036374878 | -2.513211521 | LOC102723721 |
| 231196_x_at | -1.834970237 | 6.326985291 | -3.255202269 | 0.003714004 | 0.029245518 | -2.22523068 | LINC00202-1 |
| 205801_s_at | -1.833812461 | 6.551990547 | -5.145241674 | 4.00434E-05 | 0.001232282 | 2.153296975 | RASGRP3 |
| 1561244_at | -1.832390948 | 1.711231695 | -3.012775795 | 0.006524889 | 0.043649339 | -2.757169359 | RP11-112L7.1 |
| 213765_at | -1.82916769 | 7.467143776 | -3.355914682 | 0.002930649 | 0.024936521 | -2.000244256 | MFAP5 |
| 223092_at | -1.815652454 | 9.114469413 | -10.71605436 | 4.52487E-10 | 6.43456E-07 | 13.14390223 | ANKH |
| 205413_at | -1.814990999 | 5.696781574 | -7.643083818 | 1.4824E-07 | 3.05805E-05 | 7.612640677 | MPPED2 |
| 218885_s_at | -1.81472597 | 3.435430499 | -3.131024627 | 0.004963186 | 0.036049904 | -2.499539342 | GALNT12 |
| 202458_at | -1.807589966 | 6.623626024 | -6.106268092 | 4.25503E-06 | 0.000264872 | 4.343588718 | PRSS23 |
| 216379_x_at | -1.806140438 | 6.288261659 | -3.378250999 | 0.002780128 | 0.024118003 | -1.950067883 | CD24 |
| 239975_at | -1.803624779 | 2.658999333 | -3.253943539 | 0.003724982 | 0.029305943 | -2.228029008 | HLA-DPB2 |
| 223524_s_at | -1.798696108 | 7.729112975 | -6.119998282 | 4.12385E-06 | 0.000258752 | 4.374178939 | TMEM108 |
| 220988_s_at | -1.797724562 | 4.344517753 | -3.462853871 | 0.002275538 | 0.020966153 | -1.759171279 | C1QTNF3 |
| 235619_at | -1.796515021 | 7.655521267 | -3.586234752 | 0.001696728 | 0.017021004 | -1.47859726 | ASB4 |
| 204865_at | -1.785592604 | 11.16240372 | -4.730030547 | 0.000108081 | 0.002486063 | 1.184960007 | CA3 |
| 221211_s_at | -1.784428363 | 6.366988884 | -3.987844328 | 0.000647256 | 0.008655081 | -0.551765633 | MAP3K7CL |
| 204072_s_at | -1.778817129 | 7.856059256 | -7.369066899 | 2.63662E-07 | 4.29101E-05 | 7.05394303 | FRY |
| 202403_s_at | -1.777996129 | 8.45284542 | -6.639793371 | 1.2816E-06 | 0.000123011 | 5.514917342 | COL1A2 |
| 237227_at | -1.773863022 | 4.725154222 | -5.644636188 | 1.23466E-05 | 0.000544201 | 3.302670414 | NEK10 |
| 228988_at | -1.772617141 | 3.778575274 | -3.886386862 | 0.000826396 | 0.010238198 | -0.787455976 | ZNF711 |
| 205152_at | -1.771440525 | 3.848523123 | -5.053715656 | 4.9793E-05 | 0.001435219 | 1.940604099 | SLC6A1 |
| 223893_at | -1.76882585 | 5.38198287 | -4.58085067 | 0.000154757 | 0.003163529 | 0.835524008 | ENAM |
| 203685_at | -1.763983388 | 7.192831506 | -6.597974404 | 1.40619E-06 | 0.000131165 | 5.424437799 | BCL2 |
| 225081_s_at | -1.747798909 | 5.200760629 | -7.919320842 | 8.38077E-08 | 2.0508E-05 | 8.164815898 | CDCA7L |
| 216103_at | -1.747043694 | 5.609953843 | -3.735117179 | 0.001188457 | 0.013219071 | -1.137094158 | ACOT11 |
| 1557544_at | -1.745165887 | 3.112364583 | -2.920498111 | 0.008062207 | 0.05033642 | -2.955510955 | CCDC147 |
| 203221_at | -1.743599746 | 8.130919905 | -6.122414728 | 4.10119E-06 | 0.00025847 | 4.379560333 | TLE1 |
| 202949_s_at | -1.743573578 | 7.78159988 | -5.854276798 | 7.58898E-06 | 0.000398922 | 3.77827381 | FHL2 |
| 205972_at | -1.739876855 | 4.666499873 | -3.396356799 | 0.002663681 | 0.02340583 | -1.909324798 | SLC38A3 |
| 227834_at | -1.730783883 | 9.632557171 | -4.720014119 | 0.000110715 | 0.002521707 | 1.161512977 | TXLNB |
| 242378_at | -1.723812561 | 5.235365015 | -5.750108872 | 9.6595E-06 | 0.000470732 | 3.542523652 | MYADML2 |
| 226096_at | -1.722490086 | 9.809830849 | -4.912245373 | 6.9814E-05 | 0.00184364 | 1.610912192 | FNDC5 |
| 1569858_at | -1.714832513 | 2.431277684 | -3.152519563 | 0.00472111 | 0.034800639 | -2.452319906 | LOC101927358 |
| 211764_s_at | -1.71122801 | 9.894382645 | -4.45152694 | 0.00021138 | 0.003909607 | 0.53238726 | UBE2D1 |
| 1564310_a_at | -1.709729738 | 2.835182193 | -3.486135288 | 0.002153199 | 0.020174234 | -1.706417099 | PARP15 |
| 203797_at | -1.700886253 | 5.21864743 | -3.993129249 | 0.000639064 | 0.008589116 | -0.539466655 | VSNL1 |
| 226864_at | -1.697251642 | 10.93510065 | -6.383046449 | 2.2732E-06 | 0.000177727 | 4.955778079 | PKIA |
| 205225_at | -1.696060455 | 5.820786101 | -7.585522492 | 1.67162E-07 | 3.27692E-05 | 7.496181107 | ESR1 |
| 211538_s_at | -1.685820751 | 9.825459497 | -3.996467383 | 0.000633943 | 0.008533213 | -0.531697177 | HSPA2 |
| 241749_at | -1.685178128 | 9.474104295 | -4.696609708 | 0.000117123 | 0.002614145 | 1.106716388 | MURC |
| 232936_at | -1.680969809 | 6.745235693 | -3.266370618 | 0.003617973 | 0.028782166 | -2.200386706 | KCNA7 |
| 214383_x_at | -1.680311439 | 9.217763092 | -6.774296777 | 9.52404E-07 | 0.000100612 | 5.804330036 | KLHDC3 |
| 209782_s_at | -1.677280213 | 6.470437357 | -6.328173836 | 2.57214E-06 | 0.000189346 | 4.835168077 | DBP |
| 225203_at | -1.670365302 | 7.123162529 | -6.345019618 | 2.47631E-06 | 0.000184202 | 4.87223583 | PPP1R16A |
| 229085_at | -1.667899837 | 5.843228936 | -3.331116186 | 0.003107086 | 0.025940147 | -2.055836786 | LRRC3B |
| 227265_at | -1.667056057 | 5.943206283 | -6.349217085 | 2.45301E-06 | 0.000183627 | 4.881466409 | FGL2 |
| 205826_at | -1.665083071 | 10.40398434 | -4.629178805 | 0.000137754 | 0.002905606 | 0.948772089 | MYOM2 |
| 244771_at | -1.662955486 | 8.456295833 | -6.692486728 | 1.14057E-06 | 0.000112416 | 5.628590318 | KBTBD12 |
| 209687_at | -1.662410071 | 8.426516715 | -7.548454374 | 1.80649E-07 | 3.42769E-05 | 7.420929155 | CXCL12 |
| 1560257_at | -1.660241519 | 4.350929649 | -3.486518607 | 0.00215124 | 0.020169876 | -1.705547757 | LOC101928988 |
| 228186_s_at | -1.659206638 | 7.642603238 | -6.09062134 | 4.40974E-06 | 0.000271172 | 4.30870109 | RSPO3 |
| 231032_at | -1.657776696 | 5.894320921 | -4.513168591 | 0.000182178 | 0.003565385 | 0.676883017 | LOC286071 |
| 219087_at | -1.656010885 | 6.511965678 | -6.170233611 | 3.67811E-06 | 0.000239462 | 4.485907849 | ASPN |
| 208437_at | -1.655244688 | 6.215889145 | -5.563204975 | 1.49342E-05 | 0.000619359 | 3.116735607 | CLCN1 |
| 221713_s_at | -1.650617033 | 6.773388841 | -8.49213515 | 2.65322E-08 | 9.55598E-06 | 9.274365586 | MAP6D1 |
| 221868_at | -1.649155849 | 7.592285767 | -5.222791385 | 3.33091E-05 | 0.001081438 | 2.333082562 | PAIP2B |
| 210675_s_at | -1.647851804 | 3.337656872 | -3.271096654 | 0.003578067 | 0.028619697 | -2.189865469 | PTPRR |
| 224061_at | -1.647559607 | 5.564638645 | -6.379232241 | 2.29278E-06 | 0.000178797 | 4.947406863 | INMT |
| 230143_at | -1.646366838 | 3.775849194 | -3.079977276 | 0.005587157 | 0.039163078 | -2.611210646 | RNF165 |
| 208352_x_at | -1.64461926 | 10.13012782 | -6.259973568 | 3.00059E-06 | 0.000211664 | 4.684733746 | ANK1 |
| 202428_x_at | -1.640729325 | 9.969484338 | -7.10663867 | 4.62003E-07 | 6.37851E-05 | 6.508739775 | DBI |
| 228696_at | -1.638074175 | 5.357324126 | -4.41691413 | 0.000229793 | 0.004138833 | 0.451256275 | SLC45A3 |
| 204484_at | -1.637905932 | 7.633351891 | -7.606871189 | 1.59869E-07 | 3.20479E-05 | 7.539430526 | PIK3C2B |
| 218204_s_at | -1.636450357 | 10.105739 | -7.052516129 | 5.19252E-07 | 6.95249E-05 | 6.395079119 | FYCO1 |
| 208123_at | -1.634821198 | 3.224006472 | -3.564531312 | 0.001786832 | 0.017684904 | -1.528124681 | KCNB2 |
| 234984_at | -1.632664229 | 7.882026723 | -6.779938491 | 9.40666E-07 | 0.00010023 | 5.816415608 | NEDD1 |
| 214823_at | -1.630294394 | 4.856885404 | -4.804252045 | 9.04365E-05 | 0.002200869 | 1.358608608 | ZNF204P |
| 228984_at | -1.629557294 | 8.019527865 | -4.520310874 | 0.000179068 | 0.003520045 | 0.693625222 | CARNS1 |
| 203498_at | -1.628213721 | 8.128377217 | -4.511799351 | 0.000182781 | 0.003571266 | 0.673673365 | RCAN2 |
| 211719_x_at | -1.626694464 | 8.290159379 | -5.701583247 | 1.08127E-05 | 0.000503768 | 3.432312775 | FN1 |
| 205330_at | -1.62238176 | 8.904993386 | -4.123509249 | 0.000466619 | 0.006918759 | -0.235478418 | MN1 |
| 212779_at | -1.620502849 | 8.72552354 | -10.41304091 | 7.61068E-10 | 1.00016E-06 | 12.65578905 | KIAA1109 |
| 239594_at | -1.610847837 | 2.545582896 | -3.114021124 | 0.005163145 | 0.037047161 | -2.536810339 | LOC145837 |
| 225274_at | -1.609574362 | 9.701678249 | -6.346204867 | 2.46971E-06 | 0.000184097 | 4.874842518 | PCYOX1 |
| 205638_at | -1.609518698 | 2.540142913 | -2.842290981 | 0.009631456 | 0.056739717 | -3.121582247 | BAI3 |
| 212848_s_at | -1.608972321 | 9.05864709 | -8.884880047 | 1.23628E-08 | 6.09244E-06 | 10.00764648 | C9orf3 |
| 204173_at | -1.607351293 | 10.99506521 | -3.539092857 | 0.001898442 | 0.018454938 | -1.58608475 | MYL6B |
| 224839_s_at | -1.603038549 | 7.537206503 | -6.198607797 | 3.44845E-06 | 0.000228797 | 4.548879976 | GPT2 |
| 232456_at | -1.590252111 | 9.578842577 | -5.619606058 | 1.30893E-05 | 0.000562951 | 3.245586484 | C10orf71 |
| 227210_at | -1.586530957 | 4.464875776 | -3.791111564 | 0.001039064 | 0.011970156 | -1.007949236 | SFMBT2 |
| 215930_s_at | -1.577956078 | 6.317501458 | -6.262514431 | 2.98339E-06 | 0.00021087 | 4.69034875 | CTAGE5 |
| 229484_at | -1.566870107 | 6.856982678 | -5.311215295 | 2.70171E-05 | 0.000936401 | 2.537550116 | PPM1J |
| 219197_s_at | -1.564711479 | 4.672611935 | -3.855284712 | 0.00089059 | 0.010788638 | -0.859531661 | SCUBE2 |
| 206677_at | -1.564587736 | 4.772348461 | -3.451516036 | 0.002337557 | 0.021363266 | -1.784828379 | KRT31 |
| 1563542_a_at | -1.560888989 | 3.492547093 | -3.266145696 | 0.003619883 | 0.028782166 | -2.200887314 | SCML4 |
| 1552400_a_at | -1.557878989 | 5.716255211 | -5.524860041 | 1.63377E-05 | 0.000664787 | 3.02896325 | C15orf27 |
| 213071_at | -1.554211747 | 6.628848103 | -3.709838324 | 0.001262675 | 0.013819315 | -1.195278753 | DPT |
| 229211_at | -1.553262684 | 8.44639688 | -8.442975058 | 2.92349E-08 | 1.01037E-05 | 9.181013826 | DUSP28 |
| 205934_at | -1.552945631 | 6.775765835 | -6.482500635 | 1.81887E-06 | 0.000155137 | 5.173392935 | PLCL1 |
| 223204_at | -1.5517111 | 6.73160297 | -6.177694757 | 3.61625E-06 | 0.000236945 | 4.502476221 | FAM198B |
| 228728_at | -1.549780757 | 8.795860534 | -4.410430059 | 0.000233417 | 0.004187103 | 0.436058921 | CPED1 |
| 208148_at | -1.549164818 | 6.738994072 | -4.292176286 | 0.000310536 | 0.005182598 | 0.159004803 | MYH4 |
| 206160_at | -1.548186486 | 11.1870774 | -6.043872334 | 4.90714E-06 | 0.000289709 | 4.204292131 | APOBEC2 |
| 203821_at | -1.54753692 | 7.24346468 | -6.097870392 | 4.33736E-06 | 0.000268683 | 4.324867952 | HBEGF |
| 205296_at | -1.543228077 | 5.058388625 | -5.448847311 | 1.95301E-05 | 0.000747536 | 2.854572486 | RBL1 |
| 228977_at | -1.538454747 | 7.914210978 | -3.899986385 | 0.00079979 | 0.010042147 | -0.755913133 | LOC729680 |
| 204726_at | -1.537654506 | 6.710855908 | -6.64308379 | 1.27229E-06 | 0.000122672 | 5.522026554 | CDH13 |
| 203566_s_at | -1.530240348 | 11.12161256 | -5.50533746 | 1.71032E-05 | 0.000686486 | 2.984223548 | AGL |
| 228499_at | -1.528872064 | 4.344212686 | -3.850981144 | 0.000899853 | 0.010863762 | -0.869497576 | PFKFB4 |
| 205954_at | -1.527015809 | 5.978501464 | -4.028243575 | 0.00058719 | 0.008078583 | -0.457699818 | RXRG |
| 221563_at | -1.525774019 | 7.521675269 | -4.707574771 | 0.000114076 | 0.002569923 | 1.132390539 | DUSP10 |
| 207145_at | -1.52533262 | 5.315753593 | -3.448761016 | 0.002352876 | 0.021428111 | -1.791059503 | MSTN |
| 223987_at | -1.523947453 | 4.834648111 | -2.948808548 | 0.007556979 | 0.048191362 | -2.894927146 | CHRDL2 |
| 216733_s_at | -1.517099115 | 7.459367492 | -3.065259887 | 0.005780695 | 0.04006849 | -2.643280554 | GATM |
| 201669_s_at | -1.516453397 | 7.197263423 | -4.896387751 | 7.25152E-05 | 0.001886353 | 1.573894742 | MARCKS |
| 229789_at | -1.514094198 | 2.92091204 | -3.263755883 | 0.003640237 | 0.028889037 | -2.20620562 | TIGD3 |
| 1556507_at | -1.513210023 | 7.393529584 | -5.219438793 | 3.3575E-05 | 0.001084979 | 2.32531882 | LINC01210 |
| 209447_at | -1.508598991 | 7.484205433 | -4.506851108 | 0.000184974 | 0.003602125 | 0.662074091 | SYNE1 |
| 228766_at | -1.505053641 | 9.97664799 | -5.200546943 | 3.5114E-05 | 0.001114413 | 2.28155523 | CD36 |
| 219438_at | -1.504832406 | 4.94077799 | -2.950880024 | 0.007521229 | 0.048041086 | -2.890484787 | NKAIN1 |
| 1558322_a_at | -1.502741956 | 4.526775363 | -4.191436425 | 0.00039605 | 0.006179698 | -0.076740579 | PAQR9 |
| 220449_at | -1.498954485 | 4.315388567 | -3.949671434 | 0.000709613 | 0.00921951 | -0.640539608 | LINC01260 |
| 218900_at | -1.495550666 | 6.748994055 | -7.514299383 | 1.94069E-07 | 3.51325E-05 | 7.351414679 | CNNM4 |
| 205177_at | -1.494758744 | 11.90166746 | -3.517012115 | 0.002000851 | 0.019161726 | -1.636312589 | TNNI1 |
| 210155_at | -1.493551182 | 7.007794561 | -3.56561516 | 0.001782223 | 0.017658988 | -1.525653002 | MYOC |
| 1556671_s_at | -1.490679071 | 3.0774609 | -3.357765792 | 0.002917878 | 0.024851694 | -1.996089606 | GTF3C2-AS1 |
| 235488_at | -1.484100749 | 5.721781858 | -3.49803661 | 0.002093175 | 0.019784242 | -1.67941423 | RASL10B |
| 1555310_a_at | -1.483368663 | 4.061443568 | -3.416118093 | 0.002542027 | 0.022605569 | -1.864786377 | PAK6 |
| 244455_at | -1.48119023 | 2.604426543 | -3.215065577 | 0.004080004 | 0.031301923 | -2.314285906 | KCNT2 |
| 230577_at | -1.47583998 | 6.050001493 | -3.800815247 | 0.001015134 | 0.011780835 | -0.985533856 | LINC00844 |
| 211748_x_at | -1.470677459 | 7.874007253 | -4.854964197 | 8.00804E-05 | 0.002036854 | 1.477145604 | PTGDS |
| 227300_at | -1.467721274 | 2.935735341 | -3.004665093 | 0.006647826 | 0.044235905 | -2.77470131 | TMEM119 |
| 227458_at | -1.464881087 | 5.451828551 | -6.86291536 | 7.84208E-07 | 8.91835E-05 | 5.993661983 | CD274 |
| 232080_at | -1.464199012 | 4.626810013 | -3.37815551 | 0.002780755 | 0.024118003 | -1.950282595 | HECW2 |
| 201739_at | -1.460762734 | 8.615186754 | -3.788212765 | 0.001046321 | 0.012037889 | -1.014643484 | SGK1 |
| 218432_at | -1.460689997 | 9.025641193 | -8.416093278 | 3.08317E-08 | 1.03205E-05 | 9.129818959 | FBXO3 |
| 227750_at | -1.453989636 | 7.501201249 | -6.495888448 | 1.76525E-06 | 0.000152026 | 5.202588579 | KALRN |
| 202704_at | -1.453187002 | 10.1011544 | -6.913344534 | 7.02436E-07 | 8.36371E-05 | 6.100918744 | TOB1 |
| 228275_at | -1.451946323 | 7.171530224 | -5.840739313 | 7.83016E-06 | 0.000407752 | 3.747701889 | LINC00888 |
| 209560_s_at | -1.449899514 | 7.537613722 | -5.91133335 | 6.65296E-06 | 0.000361597 | 3.906904686 | DLK1 |
| 224505_s_at | -1.446775347 | 8.608405776 | -4.574974925 | 0.000156964 | 0.003201242 | 0.821753255 | PLCD4 |
| 226773_at | -1.446356849 | 7.4188168 | -7.366951774 | 2.64847E-07 | 4.29101E-05 | 7.049588243 | PPM1K |
| 213592_at | -1.444790635 | 5.944111522 | -3.874729837 | 0.0008499 | 0.010428104 | -0.814480224 | APLNR |
| 206121_at | -1.444589011 | 11.6955301 | -4.374954782 | 0.000254283 | 0.004488631 | 0.3529203 | AMPD1 |
| 223694_at | -1.441528731 | 8.536575967 | -6.853281756 | 8.0091E-07 | 9.02966E-05 | 5.97313239 | TRIM7 |
| 211154_at | -1.436905109 | 2.928128043 | -3.226233783 | 0.003974786 | 0.030726222 | -2.289542371 | THPO |
| 205824_at | -1.433482232 | 8.419672755 | -7.014824113 | 5.6339E-07 | 7.34934E-05 | 6.31567949 | HSPB2 |
| 225835_at | -1.432885717 | 7.609288061 | -5.437205831 | 2.00722E-05 | 0.000760089 | 2.827819052 | SLC12A2 |
| 220508_at | -1.43023425 | 2.386247729 | -3.004492757 | 0.006650462 | 0.044235905 | -2.775073628 | CCT8L2 |
| 205779_at | -1.428638126 | 5.091402812 | -3.836979845 | 0.000930656 | 0.011092219 | -0.901908505 | RAMP2 |
| 243541_at | -1.428408002 | 3.216290946 | -3.021282822 | 0.006398296 | 0.043070448 | -2.738760883 | IL31RA |
| 213106_at | -1.427974882 | 8.098546668 | -4.739019956 | 0.000105772 | 0.002454538 | 1.206000574 | ATP8A1 |
| 1564212_at | -1.425953091 | 2.640979357 | -3.150446899 | 0.004743945 | 0.034910794 | -2.45687804 | LOC101929762 |
| 227198_at | -1.425206549 | 4.363043308 | -3.161460919 | 0.004623815 | 0.034293939 | -2.432644334 | AFF3 |
| 203789_s_at | -1.423573586 | 8.07998881 | -4.554234137 | 0.000165008 | 0.003311991 | 0.773141174 | SEMA3C |
| 227285_at | -1.419569986 | 7.858963718 | -6.603519812 | 1.38898E-06 | 0.00013038 | 5.436449292 | CIART |
| 202947_s_at | -1.413916452 | 8.870276798 | -5.964526093 | 5.88653E-06 | 0.000331008 | 4.026497964 | GYPC |
| 227379_at | -1.413557733 | 3.766627678 | -4.692150145 | 0.000118386 | 0.002635239 | 1.096273706 | MBOAT1 |
| 244151_at | -1.41247228 | 2.095488387 | -3.104690099 | 0.005276164 | 0.037577046 | -2.557232375 | SMLR1 |
| 213371_at | -1.41205341 | 11.86310581 | -4.667631871 | 0.000125578 | 0.002736951 | 1.038852665 | LDB3 |
| 206574_s_at | -1.411724912 | 9.987697822 | -3.02801989 | 0.006299723 | 0.042560313 | -2.724168142 | PTP4A3 |
| 201403_s_at | -1.410168495 | 10.78888025 | -5.25180742 | 3.10953E-05 | 0.001035014 | 2.400242245 | MGST3 |
| 207390_s_at | -1.409858276 | 7.685728213 | -4.725749256 | 0.000109199 | 0.002498132 | 1.174938447 | SMTN |
| 229567_at | -1.407304957 | 10.26796467 | -8.80240322 | 1.44892E-08 | 6.67669E-06 | 9.855494173 | FITM1 |
| 227088_at | -1.405062001 | 6.219700066 | -4.358695835 | 0.000264462 | 0.004622485 | 0.314822136 | PDE5A |
| 205259_at | -1.404077125 | 7.224786528 | -6.21691166 | 3.30813E-06 | 0.000221888 | 4.589450321 | NR3C2 |
| 225313_at | -1.403563262 | 8.214534942 | -7.229409897 | 3.54966E-07 | 5.3235E-05 | 6.76502629 | FAM217B |
| 231054_at | -1.402269644 | 4.360027995 | -4.500625953 | 0.000187772 | 0.003629154 | 0.647481505 | SPACA4 |
| 1556232_at | -1.398695553 | 4.821583644 | -3.289757997 | 0.003424638 | 0.0277871 | -2.148274131 | KIF6 |
| 210944_s_at | -1.397790208 | 11.20993965 | -3.633994398 | 0.001513896 | 0.015688096 | -1.369369269 | CAPN3 |
| 204939_s_at | -1.397396536 | 11.79673208 | -4.818671013 | 8.73615E-05 | 0.002145162 | 1.392321981 | PLN |
| 229638_at | -1.397081505 | 6.553475885 | -2.851001764 | 0.009443204 | 0.056040591 | -3.103181558 | IRX3 |
| 209348_s_at | -1.3968854 | 8.740803901 | -6.237994428 | 3.15372E-06 | 0.000217282 | 4.636128993 | MAF |
| 206329_at | -1.395455137 | 3.968735312 | -3.441717229 | 0.002392489 | 0.021727741 | -1.806984604 | EXTL1 |
| 227818_at | -1.394397737 | 8.055349191 | -7.587761715 | 1.66381E-07 | 3.27692E-05 | 7.500720563 | CEP85 |
| 243208_x_at | -1.392498471 | 3.800035976 | -3.243328363 | 0.00381882 | 0.029825971 | -2.251614075 | ACTL9 |
| 207537_at | -1.390331725 | 6.77553818 | -4.478292997 | 0.000198163 | 0.003734053 | 0.59512983 | PFKFB1 |
| 230422_at | -1.388034051 | 3.155838751 | -3.372798227 | 0.002816161 | 0.02427785 | -1.962325912 | FPR3 |
| 217515_s_at | -1.387803437 | 9.870059703 | -6.368129896 | 2.35076E-06 | 0.00018054 | 4.923029453 | CACNA1S |
| 212309_at | -1.386181906 | 8.095904891 | -7.308829569 | 2.99647E-07 | 4.60263E-05 | 6.929669786 | CLASP2 |
| 201801_s_at | -1.384875617 | 7.412680854 | -4.204959373 | 0.000383328 | 0.006058454 | -0.045115414 | SLC29A1 |
| 218550_s_at | -1.384603635 | 8.394001908 | -4.992729442 | 5.75931E-05 | 0.001597748 | 1.798606338 | LRRC20 |
| 1555291_at | -1.383088013 | 2.972933792 | -2.849220246 | 0.009481417 | 0.056164339 | -3.106946851 | TRPV3 |
| 227236_at | -1.382789908 | 4.236390828 | -3.860483001 | 0.000879527 | 0.010694784 | -0.847491467 | TSPAN2 |
| 235772_at | -1.378755777 | 4.471285576 | -3.20251776 | 0.004201447 | 0.031928835 | -2.342051843 | PPP3CB-AS1 |
| 212190_at | -1.378567914 | 5.205662315 | -4.308457457 | 0.000298565 | 0.005042609 | 0.197133327 | SERPINE2 |
| 219938_s_at | -1.378215012 | 6.492229488 | -4.178254807 | 0.000408856 | 0.006315708 | -0.107560428 | PSTPIP2 |
| 206353_at | -1.37036646 | 11.21577002 | -4.472912414 | 0.000200752 | 0.003767647 | 0.582517005 | COX6A2 |
| 231792_at | -1.370338897 | 9.070239448 | -3.475843213 | 0.002206466 | 0.020537729 | -1.729749633 | MYLK2 |
| 243863_at | -1.369624187 | 3.853116034 | -3.899317513 | 0.000801078 | 0.010042147 | -0.757464899 | ZCWPW2 |
| 214761_at | -1.3666967 | 9.04786518 | -3.192759821 | 0.004298305 | 0.032484016 | -2.363619022 | ZNF423 |
| 202744_at | -1.365501266 | 6.48359609 | -4.490496756 | 0.000192415 | 0.003674533 | 0.623737215 | SLC20A2 |
| 227012_at | -1.365294328 | 6.223197908 | -5.756166898 | 9.52462E-06 | 0.000466786 | 3.556265483 | SLC25A40 |
| 219983_at | -1.359846597 | 8.042205103 | -8.787095319 | 1.49238E-08 | 6.78879E-06 | 9.827147271 | HRASLS |
| 1554864_a_at | -1.359649149 | 4.612670593 | -4.134148313 | 0.00045479 | 0.006783041 | -0.210630327 | SDC3 |
| 235379_at | -1.357816218 | 6.121488453 | -3.133955776 | 0.004929482 | 0.035893266 | -2.493107036 | LOC100506114 |
| 208204_s_at | -1.354868555 | 8.821923927 | -6.402210042 | 2.17739E-06 | 0.00017477 | 4.997809079 | CAV3 |
| 240479_at | -1.350949328 | 4.195472784 | -2.86219909 | 0.009206368 | 0.05504893 | -3.07949216 | HS3ST5 |
| 235225_at | -1.346745021 | 5.433237735 | -4.83819722 | 8.33647E-05 | 0.002083062 | 1.437964509 | SCN2B |
| 226534_at | -1.345114714 | 6.658966159 | -5.732355375 | 1.00661E-05 | 0.000480707 | 3.502230234 | KITLG |
| 202241_at | -1.342108814 | 9.213815951 | -6.118508366 | 4.13788E-06 | 0.000258942 | 4.370860567 | TRIB1 |
| 223681_s_at | -1.341677987 | 8.211078673 | -5.262172156 | 3.03413E-05 | 0.001014674 | 2.424217189 | INADL |
| 227283_at | -1.341386819 | 5.557600064 | -7.996074569 | 7.1656E-08 | 1.84239E-05 | 8.316263992 | EFR3B |
| 1568696_at | -1.337987483 | 6.603546881 | -5.129499037 | 4.15709E-05 | 0.001257016 | 2.116750932 | CDNF |
| 217080_s_at | -1.335304261 | 7.128114344 | -4.450464082 | 0.000211923 | 0.003913248 | 0.529895856 | HOMER2 |
| 202340_x_at | -1.331393711 | 6.773386124 | -4.36286334 | 0.000261815 | 0.004592048 | 0.324587089 | NR4A1 |
| 213245_at | -1.329535472 | 5.82015241 | -4.668375786 | 0.000125353 | 0.002734591 | 1.040595084 | ADCY1 |
| 206216_at | -1.328997863 | 9.04627273 | -5.674597405 | 1.15138E-05 | 0.000523967 | 3.370918881 | SRPK3 |
| 230466_s_at | -1.32549365 | 7.913816741 | -3.332536199 | 0.003096709 | 0.025890063 | -2.052656763 | RASSF3 |
| 1553873_at | -1.321105526 | 6.866337313 | -3.232294807 | 0.003918792 | 0.030405986 | -2.276102104 | KLHL34 |
| 218494_s_at | -1.321005028 | 7.983414923 | -7.491140088 | 2.0375E-07 | 3.58175E-05 | 7.304183178 | SLC2A4RG |
| 238878_at | -1.319367965 | 8.374458795 | -5.40941705 | 2.14291E-05 | 0.000791205 | 2.763910099 | ARX |
| 235904_at | -1.318634892 | 6.27371593 | -3.946358989 | 0.000715297 | 0.00926455 | -0.64823769 | UGT3A1 |
| 209763_at | -1.314079087 | 6.187645101 | -5.408183487 | 2.14915E-05 | 0.000792683 | 2.76107161 | CHRDL1 |
| 1553087_at | -1.307387064 | 3.68136129 | -2.873604428 | 0.00897097 | 0.054079403 | -3.055321278 | C18orf12 |
| 222853_at | -1.307083307 | 6.940592833 | -4.874555479 | 7.64079E-05 | 0.001965992 | 1.522912167 | FLRT3 |
| 225381_at | -1.307061759 | 5.347853256 | -6.654053928 | 1.24175E-06 | 0.000120382 | 5.5457179 | MIR100HG |
| 227870_at | -1.305694811 | 7.500649502 | -6.593303228 | 1.42085E-06 | 0.000131288 | 5.414316733 | IGDCC4 |
| 227337_at | -1.30347399 | 7.244723267 | -4.715179313 | 0.000112009 | 0.002541115 | 1.150194425 | ANKRD37 |
| 205331_s_at | -1.29973665 | 6.351497078 | -4.244382404 | 0.000348522 | 0.005651858 | 0.047118998 | REEP2 |
| 209765_at | -1.299440085 | 5.673357369 | -5.751249461 | 9.63396E-06 | 0.000470196 | 3.54511122 | ADAM19 |
| 226188_at | -1.296781765 | 9.200042074 | -3.625805265 | 0.001543805 | 0.015897075 | -1.388120886 | LGALSL |
| 213658_at | -1.296552753 | 8.229500525 | -5.743188918 | 9.81596E-06 | 0.000475157 | 3.526821979 | AK055981 |
| 205151_s_at | -1.296050829 | 5.024954278 | -3.622472431 | 0.001556144 | 0.015990469 | -1.395749811 | TRIL |
| 219165_at | -1.295381367 | 7.838249435 | -5.698065984 | 1.09015E-05 | 0.000506293 | 3.424315031 | PDLIM2 |
| 206586_at | -1.287149715 | 4.608679124 | -3.639488574 | 0.00149415 | 0.015579023 | -1.356783463 | CNR2 |
| 230440_at | -1.283346884 | 5.489954171 | -4.573297927 | 0.000157599 | 0.003210067 | 0.817822875 | ZNF469 |
| 204159_at | -1.280028963 | 6.213623631 | -4.612895295 | 0.000143262 | 0.002983117 | 0.910618383 | CDKN2C |
| 208100_x_at | -1.279529385 | 6.44705167 | -4.67819752 | 0.000122426 | 0.002685838 | 1.063598724 | SEMA6C |
| 226301_at | -1.278238333 | 5.17528093 | -4.727953819 | 0.000108622 | 0.002491356 | 1.180098907 | SLC18B1 |
| 225464_at | -1.275373517 | 6.346432264 | -6.085696383 | 4.45962E-06 | 0.000272291 | 4.297713875 | FRMD6 |
| 206973_at | -1.270444913 | 3.457112791 | -3.102400317 | 0.005304261 | 0.037722053 | -2.562240419 | PPFIA2 |
| 235764_at | -1.269627758 | 3.305582216 | -3.573220808 | 0.001750206 | 0.017434248 | -1.508303601 | PRDM5 |
| 211597_s_at | -1.269376593 | 6.805709186 | -4.856505159 | 7.97852E-05 | 0.002030801 | 1.480745967 | HOPX |
| 229402_at | -1.264997384 | 5.118864874 | -3.441014217 | 0.002396478 | 0.021751528 | -1.808573543 | SAMD13 |
| 205848_at | -1.262818087 | 6.337996904 | -6.356079571 | 2.41539E-06 | 0.000183357 | 4.896552763 | GAS2 |
| 224723_x_at | -1.259295575 | 7.87883249 | -4.495101577 | 0.00019029 | 0.003653786 | 0.634531585 | LINC00998 |
| 225389_at | -1.256583682 | 9.108977649 | -7.632653944 | 1.51497E-07 | 3.08932E-05 | 7.591574416 | BTBD6 |
| 213125_at | -1.25371554 | 5.552191459 | -5.790943776 | 8.78664E-06 | 0.000443944 | 3.63507777 | OLFML2B |
| 228646_at | -1.253341858 | 4.768930564 | -3.534789515 | 0.001917988 | 0.018588928 | -1.595879762 | PPP1R1C |
| 212504_at | -1.251267964 | 8.983351442 | -7.167453049 | 4.05359E-07 | 5.84672E-05 | 6.635958635 | DIP2C |
| 228504_at | -1.250714689 | 6.630356402 | -6.384142875 | 2.26761E-06 | 0.000177727 | 4.958184112 | SCN7A |
| 236352_at | -1.250392228 | 8.677758305 | -4.934433433 | 6.62042E-05 | 0.001776132 | 1.662687808 | VGLL2 |
| 212573_at | -1.250373358 | 8.422235644 | -6.977876168 | 6.10408E-07 | 7.74196E-05 | 6.23765351 | ENDOD1 |
| 207772_s_at | -1.246966347 | 3.353809037 | -3.00585178 | 0.0066297 | 0.044167298 | -2.772137346 | PRMT8 |
| 219304_s_at | -1.246499326 | 5.498539574 | -4.639445259 | 0.000134391 | 0.002862232 | 0.972825066 | PDGFD |
| 236688_at | -1.244937807 | 3.537693792 | -2.939012717 | 0.00772826 | 0.048940591 | -2.915917344 | FRMPD3 |
| 218918_at | -1.244231632 | 5.21297185 | -5.967239107 | 5.84995E-06 | 0.000329473 | 4.032589058 | MAN1C1 |
| 239057_at | -1.242068395 | 11.722399 | -5.045551011 | 5.07719E-05 | 0.001455159 | 1.921606022 | LMOD2 |
| 210088_x_at | -1.241359233 | 6.934296508 | -5.03603872 | 5.19369E-05 | 0.001482144 | 1.899467309 | MYL4 |
| 204040_at | -1.239549895 | 5.981211501 | -6.852871139 | 8.0163E-07 | 9.02966E-05 | 5.972257064 | RNF144A |
| 207929_at | -1.237297178 | 3.896665045 | -2.912529614 | 0.008210151 | 0.050875405 | -2.972519529 | GRPR |
| 206191_at | -1.236458304 | 4.64342454 | -6.391249971 | 2.23167E-06 | 0.00017675 | 4.973776413 | ENTPD3 |
| 206373_at | -1.231516879 | 6.446670629 | -3.884806107 | 0.000829545 | 0.010255726 | -0.791121329 | ZIC1 |
| 209199_s_at | -1.230074309 | 11.32793281 | -4.835823949 | 8.38405E-05 | 0.002091322 | 1.432417757 | MEF2C |
| 219891_at | -1.229753793 | 6.455705469 | -3.587841988 | 0.001690236 | 0.016975151 | -1.474926764 | PGPEP1 |
| 244716_x_at | -1.228623641 | 5.847906591 | -2.957505428 | 0.00740797 | 0.047600434 | -2.876267806 | TMIGD2 |
| 228150_at | -1.224823985 | 5.430898558 | -4.426254173 | 0.000224671 | 0.004078202 | 0.473148106 | SEC16B |
| 1558603_at | -1.219625806 | 3.535617966 | -3.109682104 | 0.005215406 | 0.037271506 | -2.546309559 | PLGLB2 |
| 203886_s_at | -1.219184584 | 5.599816702 | -3.641056154 | 0.001488562 | 0.015539032 | -1.353191769 | FBLN2 |
| 205618_at | -1.218223744 | 7.49359784 | -7.178148535 | 3.9616E-07 | 5.80048E-05 | 6.658278399 | PRRG1 |
| 237329_at | -1.218059784 | 5.976591633 | -5.453363405 | 1.93238E-05 | 0.00074166 | 2.864947802 | RBMS3-AS3 |
| 205610_at | -1.217615918 | 10.4995798 | -3.802133933 | 0.001011924 | 0.011768303 | -0.982486942 | MYOM1 |
| 212354_at | -1.217529272 | 5.063208057 | -4.551740637 | 0.000166002 | 0.003323983 | 0.76729668 | SULF1 |
| 207761_s_at | -1.217226538 | 9.870145872 | -4.11504116 | 0.000476254 | 0.007000184 | -0.255252014 | METTL7A |
| 203616_at | -1.215122891 | 8.73511755 | -6.501855068 | 1.74188E-06 | 0.000150378 | 5.215592803 | POLB |
| 204418_x_at | -1.214033689 | 9.036871886 | -4.365479163 | 0.000260167 | 0.004567658 | 0.330716411 | GSTM2 |
| 204442_x_at | -1.213627378 | 6.164944051 | -4.013924211 | 0.000607818 | 0.008287731 | -0.491053883 | LTBP4 |
| 228656_at | -1.212816306 | 6.644786867 | -5.065013258 | 4.84699E-05 | 0.001406625 | 1.966885749 | PROX1 |
| 1558801_at | -1.211735593 | 8.90490599 | -6.238639617 | 3.14912E-06 | 0.000217282 | 4.637556619 | NNT-AS1 |
| 212742_at | -1.211532577 | 9.541872565 | -3.817689349 | 0.000974812 | 0.011460663 | -0.946531493 | RNF115 |
| 227522_at | -1.210655638 | 10.8154468 | -5.681316648 | 1.1335E-05 | 0.000518952 | 3.386212299 | CMBL |
| 216481_at | -1.209140573 | 6.293162585 | -3.617212284 | 0.001575815 | 0.016117924 | -1.40778725 | GRIP2 |
| 45714_at | -1.208199958 | 8.854611966 | -8.367484713 | 3.39519E-08 | 1.07561E-05 | 9.036980395 | HCFC1R1 |
| 236656_s_at | -1.208134572 | 6.993653268 | -5.764238025 | 9.3479E-06 | 0.000461154 | 3.574567811 | LOC100288911 |
| 212104_s_at | -1.207545847 | 8.662342309 | -5.070217313 | 4.78725E-05 | 0.001395328 | 1.978989423 | RBFOX2 |
| 205761_s_at | -1.20721314 | 5.303514025 | -3.311664577 | 0.00325269 | 0.026833751 | -2.099356031 | DUS4L |
| 1553079_at | -1.205598598 | 2.948516557 | -3.15535284 | 0.004690067 | 0.034647714 | -2.446087354 | TRIM40 |
| 213341_at | -1.203116251 | 7.495854563 | -3.593515528 | 0.001667513 | 0.016831557 | -1.461966908 | FEM1C |
| 230419_at | -1.201296398 | 6.8314085 | -4.213471822 | 0.00037553 | 0.005969775 | -0.025204343 | SOX9-AS1 |
| 1561218_s_at | -1.200578132 | 3.907688824 | -3.000747113 | 0.006708007 | 0.04448012 | -2.783163692 | LOC728099 |
| 213702_x_at | -1.198802601 | 9.877001446 | -4.786766426 | 9.43124E-05 | 0.002271822 | 1.317715208 | ASAH1 |
| 231851_at | -1.198519179 | 5.831112871 | -5.348849857 | 2.47189E-05 | 0.000882369 | 2.624392224 | RAVER2 |
| 212895_s_at | -1.198198177 | 6.756518407 | -8.04498237 | 6.48755E-08 | 1.71785E-05 | 8.412318806 | ABR |
| 205475_at | -1.196102789 | 4.160912935 | -4.631624169 | 0.000136946 | 0.002894047 | 0.954501424 | SCRG1 |
| 219696_at | -1.195837582 | 7.926336981 | -5.421886619 | 2.08091E-05 | 0.000776391 | 2.792595929 | DENND1B |
| 202969_at | -1.195780999 | 8.035065416 | -5.382644962 | 2.28246E-05 | 0.000830628 | 2.70227746 | DYRK2 |
| 206612_at | -1.194641856 | 9.098388027 | -3.550737448 | 0.001846527 | 0.018084037 | -1.559565558 | CACNG1 |
| 227970_at | -1.192644309 | 7.951400627 | -4.482664469 | 0.000196085 | 0.003708676 | 0.605377187 | GPR157 |
| 228621_at | -1.192481541 | 10.46271487 | -4.450148633 | 0.000212084 | 0.003913248 | 0.529156427 | HFE2 |
| 1564139_at | -1.190876958 | 5.650536477 | -5.271311492 | 2.96918E-05 | 0.000999306 | 2.445350979 | A2M-AS1 |
| 221659_s_at | -1.189052888 | 6.33990679 | -2.945333749 | 0.007617316 | 0.048462899 | -2.902376119 | MYL10 |
| 219144_at | -1.188891119 | 9.25730873 | -5.292318254 | 2.82518E-05 | 0.00096295 | 2.493903067 | DUSP26 |
| 212886_at | -1.177867688 | 9.017184905 | -4.102821403 | 0.000490508 | 0.007138717 | -0.283779458 | CCDC69 |
| 202766_s_at | -1.174319021 | 7.850687704 | -3.332649498 | 0.003095882 | 0.025890063 | -2.052403021 | FBN1 |
| 204917_s_at | -1.170065433 | 4.946633953 | -3.783409151 | 0.001058456 | 0.012131145 | -1.025734572 | MLLT3 |
| 211386_at | -1.168815107 | 4.850412946 | -2.829172291 | 0.009921738 | 0.058073758 | -3.149247362 | MGC12488 |
| 240490_at | -1.167377085 | 7.311896972 | -3.314571418 | 0.003230516 | 0.026719155 | -2.092857457 | LOC101929592 |
| 212298_at | -1.166039725 | 7.007965982 | -3.753585165 | 0.001136976 | 0.012815184 | -1.094538724 | NRP1 |
| 205771_s_at | -1.163867166 | 6.965486656 | -4.730327453 | 0.000108004 | 0.002486063 | 1.185654978 | AKAP7 |
| 215239_x_at | -1.161506487 | 5.072648297 | -4.148095082 | 0.000439734 | 0.006636605 | -0.17804858 | ZNF273 |
| 219493_at | -1.161468385 | 3.320888482 | -3.028389447 | 0.006294358 | 0.042548374 | -2.723367304 | SHCBP1 |
| 213150_at | -1.157262967 | 8.667338979 | -3.956296361 | 0.000698378 | 0.009110239 | -0.625140778 | HOXA10 |
| 208782_at | -1.156334889 | 8.291700186 | -3.453140533 | 0.002328571 | 0.021316393 | -1.781153577 | FSTL1 |
| 217967_s_at | -1.15631437 | 9.863476816 | -3.202720449 | 0.004199457 | 0.031920554 | -2.341603621 | FAM129A |
| 204439_at | -1.156048394 | 5.507047119 | -3.561731577 | 0.001798793 | 0.017753761 | -1.534508565 | IFI44L |
| 205933_at | -1.154312427 | 7.919065512 | -4.114785988 | 0.000476547 | 0.007001596 | -0.255847801 | SETBP1 |
| 52651_at | -1.154139391 | 3.289384299 | -3.135464404 | 0.004912221 | 0.035804321 | -2.489795565 | COL8A2 |
| 237331_s_at | -1.151164203 | 4.374593857 | -3.387821717 | 0.002717968 | 0.023740573 | -1.928538835 | MAPT-AS1 |
| 203908_at | -1.150565067 | 5.331861137 | -4.633237016 | 0.000136415 | 0.002887992 | 0.958280171 | SLC4A4 |
| 242159_at | -1.150310265 | 4.708718782 | -3.616098519 | 0.001580012 | 0.016152466 | -1.410335519 | RP11-25I15.3 |
| 218818_at | -1.147358626 | 10.18252158 | -6.468037157 | 1.87867E-06 | 0.000157586 | 5.141825212 | FHL3 |
| 227417_at | -1.145701073 | 7.056477473 | -9.852167911 | 2.04887E-09 | 2.01939E-06 | 11.72074668 | MARC2 |
| 201036_s_at | -1.145242444 | 9.481716056 | -4.193652204 | 0.000393937 | 0.006168428 | -0.071559187 | HADH |
| 201193_at | -1.144625069 | 7.785606782 | -6.946651171 | 6.53282E-07 | 8.07171E-05 | 6.171564081 | IDH1 |
| 205990_s_at | -1.14442057 | 5.603691776 | -3.39547074 | 0.002669267 | 0.023429338 | -1.911320115 | WNT5A |
| 201236_s_at | -1.143080376 | 9.380267493 | -5.181689166 | 3.67216E-05 | 0.001155102 | 2.237845643 | BTG2 |
| 202236_s_at | -1.140782744 | 8.856991626 | -6.234623478 | 3.17791E-06 | 0.000218102 | 4.628669168 | SLC16A1 |
| 215333_x_at | -1.14029224 | 8.692257492 | -4.006144543 | 0.000619325 | 0.008406618 | -0.509169248 | GSTM1 |
| 1556043_a_at | -1.139059674 | 4.921532888 | -2.963354337 | 0.007309347 | 0.047180327 | -2.863706274 | TTN-AS1 |
| 205051_s_at | -1.138963722 | 4.141580779 | -2.930692262 | 0.007876662 | 0.049556595 | -2.933723531 | KIT |
| 218552_at | -1.138218143 | 8.261267504 | -7.573035999 | 1.71585E-07 | 3.33215E-05 | 7.470854569 | ECHDC2 |
| 1563318_s_at | -1.136959505 | 5.9455235 | -4.563297742 | 0.000161443 | 0.00325522 | 0.794384858 | MAGIX |
| 211715_s_at | -1.134706551 | 6.451472929 | -4.681626821 | 0.000121421 | 0.002675927 | 1.071630049 | BDH1 |
| 209760_at | -1.132012562 | 7.077526399 | -4.528652885 | 0.000175502 | 0.003470267 | 0.713179451 | KIAA0922 |
| 203729_at | -1.130706515 | 6.340253773 | -3.422225582 | 0.00250554 | 0.022374366 | -1.851006667 | EMP3 |
| 206101_at | -1.130200525 | 6.203571007 | -3.984861113 | 0.000651927 | 0.008689582 | -0.558707233 | ECM2 |
| 244453_at | -1.130117174 | 4.602688814 | -2.962251126 | 0.007327852 | 0.04726538 | -2.86607638 | ANKRD53 |
| 202202_s_at | -1.130111169 | 5.832696451 | -4.287840906 | 0.000313804 | 0.005222504 | 0.148852996 | LAMA4 |
| 206073_at | -1.129650258 | 5.698643893 | -3.114075202 | 0.005162497 | 0.037047161 | -2.536691918 | COLQ |
| 1569986_x_at | -1.12870109 | 12.08066709 | -3.982797245 | 0.000655178 | 0.008716539 | -0.563509239 | TNNT3 |
| 202983_at | -1.12855947 | 7.536253489 | -8.59653843 | 2.16151E-08 | 8.33639E-06 | 9.471460581 | HLTF |
| 242358_at | -1.128127559 | 6.424484956 | -5.424690555 | 2.06722E-05 | 0.000772825 | 2.799044464 | RASSF8-AS1 |
| 211828_s_at | -1.12679918 | 7.387055591 | -3.989568199 | 0.000644573 | 0.008635495 | -0.54775409 | TNIK |
| 205226_at | -1.126623842 | 6.1505146 | -3.739547643 | 0.001175901 | 0.013120539 | -1.126888719 | PDGFRL |
| 205872_x_at | -1.124701916 | 11.56656742 | -3.553845635 | 0.001832907 | 0.017995354 | -1.552483484 | PDE4DIP |
| 202967_at | -1.123025519 | 8.242140324 | -6.288856237 | 2.81086E-06 | 0.000202302 | 4.748513564 | GSTA4 |
| 212798_s_at | -1.122413817 | 8.217032578 | -5.036295661 | 5.19051E-05 | 0.001482144 | 1.900065375 | ANKMY2 |
| 205444_at | -1.121915258 | 12.34706338 | -2.843352352 | 0.009608328 | 0.056631677 | -3.119341526 | ATP2A1 |
| 207427_at | -1.121298757 | 4.15407318 | -3.307582596 | 0.00328408 | 0.026992289 | -2.108478814 | ACR |
| 213832_at | -1.120751245 | 5.4880716 | -2.944781777 | 0.007626942 | 0.048498059 | -2.903559057 | KCND3 |
| 227803_at | -1.120268206 | 6.097633367 | -3.043128812 | 0.006083947 | 0.041524134 | -2.691396117 | ENPP5 |
| 203939_at | -1.119481415 | 4.432115723 | -2.828376372 | 0.009939616 | 0.058149621 | -3.150923998 | NT5E |
| 213208_at | -1.118274422 | 6.811964251 | -3.291765035 | 0.003408523 | 0.027707034 | -2.14379654 | GLTSCR1L |
| 219064_at | -1.114977179 | 6.010727491 | -5.438458313 | 2.00132E-05 | 0.000759707 | 2.830697962 | ITIH5 |
| 212224_at | -1.114446713 | 7.274786477 | -3.995850217 | 0.000634886 | 0.008542052 | -0.533133686 | ALDH1A1 |
| 216698_x_at | -1.111036327 | 5.84141815 | -3.264138287 | 0.003636972 | 0.028876047 | -2.205354699 | OR7E12P |
| 237284_at | -1.110690599 | 5.085507651 | -3.804619408 | 0.001005902 | 0.011732875 | -0.976743591 | DNAJB8 |
| 226065_at | -1.109814148 | 6.371722081 | -5.617092808 | 1.31663E-05 | 0.000564895 | 3.239851361 | PRICKLE1 |
| 1564117_at | -1.10928365 | 4.994717471 | -5.652077478 | 1.21342E-05 | 0.00053751 | 3.319629202 | HMCN2 |
| 229849_at | -1.109206269 | 9.125736738 | -4.260361135 | 0.000335332 | 0.005480448 | 0.08451783 | WIPF3 |
| 209750_at | -1.108564699 | 9.33090024 | -4.664275466 | 0.000126596 | 0.002755757 | 1.030991055 | NR1D2 |
| 228364_at | -1.107992464 | 7.28374448 | -5.670590984 | 1.16217E-05 | 0.000523967 | 3.361797859 | ZNF784 |
| 227113_at | -1.105876616 | 8.314083149 | -4.526723596 | 0.000176321 | 0.003479537 | 0.708657096 | ADHFE1 |
| 230174_at | -1.104447632 | 8.0052619 | -4.751207961 | 0.000102719 | 0.002408915 | 1.234524056 | LYPLAL1 |
| 200884_at | -1.101814007 | 7.131723515 | -3.113731113 | 0.005166622 | 0.037057225 | -2.537445396 | CKB |
| 1552476_s_at | -1.101545091 | 6.54311075 | -3.934155125 | 0.000736633 | 0.009459724 | -0.67659179 | PLCD3 |
| 225777_at | -1.100081819 | 5.102174485 | -2.993581322 | 0.006819434 | 0.045076359 | -2.798629689 | SAPCD2 |
| 202502_at | -1.100013962 | 10.5732062 | -4.782415711 | 9.53026E-05 | 0.00229073 | 1.307538632 | ACADM |
| 218970_s_at | -1.098328721 | 10.35673054 | -4.81674526 | 8.7766E-05 | 0.002151692 | 1.387819764 | CUTC |
| 1552474_a_at | -1.098070979 | 10.57642563 | -5.735641897 | 9.98954E-06 | 0.000479807 | 3.5096918 | GAMT |
| 212845_at | -1.09764912 | 10.28053721 | -4.930349563 | 6.68541E-05 | 0.001783548 | 1.653159875 | SAMD4A |
| 213169_at | -1.094526806 | 4.915448706 | -5.565043228 | 1.48701E-05 | 0.000618546 | 3.120939956 | SEMA5A |
| 219114_at | -1.093675963 | 6.840000062 | -6.548615296 | 1.56937E-06 | 0.000140263 | 5.317344516 | C3orf18 |
| 210886_x_at | -1.092739489 | 7.127435814 | -5.67297829 | 1.15573E-05 | 0.000523967 | 3.367232997 | TP53TG1 |
| 213293_s_at | -1.092663727 | 6.741554317 | -4.729999042 | 0.000108089 | 0.002486063 | 1.184886262 | TRIM22 |
| 205088_at | -1.090099489 | 5.430327229 | -4.533629389 | 0.00017341 | 0.003447013 | 0.724844548 | MAMLD1 |
| 202893_at | -1.089964965 | 7.530577552 | -5.989017549 | 5.56458E-06 | 0.000317431 | 4.081454268 | UNC13B |
| 223082_at | -1.089328673 | 7.105657586 | -4.731944917 | 0.000107585 | 0.002482014 | 1.189440961 | SH3KBP1 |
| 208874_x_at | -1.088383034 | 8.163036241 | -7.102086805 | 4.66557E-07 | 6.41643E-05 | 6.499196533 | PPP2R4 |
| 209550_at | -1.087687753 | 6.204368954 | -4.644685846 | 0.000132707 | 0.002837908 | 0.98510237 | NDN |
| 206542_s_at | -1.086938311 | 8.84498112 | -5.493225158 | 1.75963E-05 | 0.0006976 | 2.956448498 | SMARCA2 |
| 209094_at | -1.085817991 | 9.322566887 | -3.694048983 | 0.001311334 | 0.014172629 | -1.2315819 | DDAH1 |
| 227678_at | -1.084779871 | 7.09822553 | -6.25563674 | 3.03019E-06 | 0.000212485 | 4.675148016 | XRCC6BP1 |
| 1552882_a_at | -1.08246971 | 3.755240661 | -2.918282067 | 0.008103092 | 0.050461715 | -2.960243001 | AMER1 |
| 226388_at | -1.081718152 | 11.40829379 | -6.35477796 | 2.42248E-06 | 0.000183357 | 4.893691788 | TCEA3 |
| 220559_at | -1.081540423 | 6.603364572 | -5.464599957 | 1.882E-05 | 0.00072901 | 2.890755001 | EN1 |
| 204719_at | -1.076593272 | 8.298729335 | -5.105765394 | 4.39862E-05 | 0.001311528 | 2.061623902 | ABCA8 |
| 227112_at | -1.075347633 | 8.223538588 | -8.31487537 | 3.7699E-08 | 1.13604E-05 | 8.936113917 | TMCC1 |
| 219035_s_at | -1.074448684 | 8.987678767 | -5.656977615 | 1.19964E-05 | 0.000536089 | 3.33079368 | RNF34 |
| 240043_at | -1.073187485 | 4.918693299 | -3.105678646 | 0.005264078 | 0.037520176 | -2.555069881 | PRR32 |
| 228098_s_at | -1.072106768 | 9.274853143 | -4.826311568 | 8.5775E-05 | 0.002120885 | 1.410183495 | MYLIP |
| 227702_at | -1.071042431 | 6.222243617 | -4.061908836 | 0.000541397 | 0.007598827 | -0.379231344 | CYP4X1 |
| 1553253_at | -1.069144906 | 6.824733069 | -3.885366133 | 0.000828428 | 0.010249059 | -0.789822802 | ASB16 |
| 220831_at | -1.067032915 | 3.323771022 | -3.199015762 | 0.004235962 | 0.032129199 | -2.349794584 | GCNT4 |
| 229313_at | -1.063803508 | 9.351361102 | -4.384913222 | 0.000248244 | 0.004395303 | 0.376256965 | ANO5 |
| 210189_at | -1.062804661 | 5.81168551 | -3.238412559 | 0.003863051 | 0.03007873 | -2.262527648 | HSPA1L |
| 207134_x_at | -1.062347918 | 5.378519809 | -3.081720659 | 0.005564649 | 0.039105742 | -2.607407937 | TPSB2 |
| 206637_at | -1.060996013 | 6.268153553 | -4.160514554 | 0.000426747 | 0.006504223 | -0.149027171 | P2RY14 |
| 220703_at | -1.060058462 | 5.842748209 | -3.926319747 | 0.000750664 | 0.009598215 | -0.694789924 | IDI2-AS1 |
| 203516_at | -1.059631512 | 8.866016427 | -5.502912912 | 1.72007E-05 | 0.000688844 | 2.978664812 | SNTA1 |
| 223634_at | -1.058070229 | 5.925914678 | -4.678029303 | 0.000122476 | 0.002685838 | 1.063204758 | RASD2 |
| 1564537_a_at | -1.057232161 | 6.904590282 | -3.896479168 | 0.000806569 | 0.010087658 | -0.764049358 | ASB10 |
| 218346_s_at | -1.0549565 | 9.924065961 | -5.790494096 | 8.7958E-06 | 0.000443944 | 3.634059506 | SESN1 |
| 201319_at | -1.054049988 | 10.81537051 | -4.815544457 | 8.80192E-05 | 0.002154596 | 1.385012337 | MYL12A |
| 1562031_at | -1.051208558 | 6.040842452 | -3.479077057 | 0.002189592 | 0.020428895 | -1.722420309 | JAK2 |
| 201690_s_at | -1.050459261 | 6.732578659 | -4.222372619 | 0.000367545 | 0.005877078 | -0.004382046 | TPD52 |
| 209539_at | -1.050153669 | 9.061421321 | -5.504481689 | 1.71375E-05 | 0.000687089 | 2.982261592 | ARHGEF6 |
| 213411_at | -1.047719814 | 4.003787599 | -3.061647658 | 0.005829176 | 0.040262958 | -2.65114297 | ADAM22 |
| 206331_at | -1.043643733 | 4.649790829 | -2.846250149 | 0.009545453 | 0.056392235 | -3.113221949 | CALCRL |
| 227946_at | -1.041652012 | 6.126438136 | -3.605951937 | 0.001618753 | 0.016476358 | -1.433542603 | OSBPL7 |
| 208796_s_at | -1.041547592 | 10.86424484 | -7.693378561 | 1.33518E-07 | 2.8539E-05 | 7.714003819 | CCNG1 |
| 218087_s_at | -1.040818463 | 10.30783094 | -3.955859991 | 0.000699113 | 0.009114051 | -0.626155172 | SORBS1 |
| 203502_at | -1.040806609 | 6.684310226 | -6.439276665 | 2.00365E-06 | 0.000165226 | 5.078972195 | BPGM |
| 206209_s_at | -1.040727528 | 5.99866918 | -3.385276116 | 0.002734367 | 0.023833007 | -1.93426679 | CA4 |
| 226925_at | -1.039804924 | 5.076288999 | -3.35331348 | 0.002948687 | 0.025023179 | -2.006081284 | PXYLP1 |
| 209337_at | -1.039068016 | 8.360058883 | -5.900383654 | 6.82298E-06 | 0.000365917 | 3.882247101 | PSIP1 |
| 37408_at | -1.037342652 | 7.285120542 | -6.814654334 | 8.71634E-07 | 9.4579E-05 | 5.890687179 | MRC2 |
| 218306_s_at | -1.036075581 | 9.393895191 | -7.595988895 | 1.63544E-07 | 3.25381E-05 | 7.517392823 | HERC1 |
| 207981_s_at | -1.035081026 | 7.523986677 | -4.953022271 | 6.33255E-05 | 0.001721775 | 1.706046615 | ESRRG |
| 204092_s_at | -1.031846656 | 6.062035193 | -3.05007324 | 0.005987176 | 0.041074438 | -2.676312361 | AURKA |
| 225673_at | -1.030026104 | 7.008535576 | -4.71301076 | 0.000112594 | 0.002545044 | 1.145117523 | MYADM |
| 225171_at | -1.029477815 | 6.933525993 | -5.440635364 | 1.99109E-05 | 0.000759656 | 2.83570173 | ARHGAP18 |
| 209780_at | -1.029379685 | 10.11358559 | -4.800114352 | 9.13389E-05 | 0.0022107 | 1.348932791 | PHTF2 |
| 234305_s_at | -1.026382144 | 6.658646257 | -3.317279891 | 0.003209988 | 0.026567946 | -2.086800792 | GSDMC |
| 227250_at | -1.026278067 | 6.676131791 | -5.121679934 | 4.23514E-05 | 0.001275649 | 2.098593168 | KREMEN1 |
| 1552733_at | -1.024678467 | 7.029641623 | -5.910914989 | 6.65937E-06 | 0.000361597 | 3.905962827 | KLHDC1 |
| 206768_at | -1.023430107 | 10.69634435 | -3.288024088 | 0.003438619 | 0.027862316 | -2.152141698 | RPL3L |
| 204754_at | -1.02266365 | 6.374323866 | -4.517345235 | 0.000180353 | 0.003537465 | 0.686673498 | HLF |
| 203608_at | -1.021590367 | 8.508507103 | -4.879576647 | 7.54943E-05 | 0.001949555 | 1.534639381 | ALDH5A1 |
| 229553_at | -1.020494399 | 7.8128125 | -3.462397458 | 0.002278004 | 0.020983417 | -1.760204555 | PGM2L1 |
| 205433_at | -1.019695303 | 4.961832509 | -3.203569709 | 0.004191132 | 0.031877761 | -2.33972548 | BCHE |
| 223986_x_at | -1.016697507 | 5.585430261 | -3.382232084 | 0.002754105 | 0.023957132 | -1.941114668 | DMRT2 |
| 236359_at | -1.016153956 | 6.412547471 | -3.522385579 | 0.001975441 | 0.018984992 | -1.624096519 | SCN4B |
| 221729_at | -1.015153961 | 5.853926741 | -3.038036494 | 0.006155864 | 0.041884426 | -2.702448615 | COL5A2 |
| 219090_at | -1.014867953 | 8.09954701 | -3.266120618 | 0.003620096 | 0.028782166 | -2.200943128 | SLC24A3 |
| 202746_at | -1.014638177 | 7.869533002 | -4.905361946 | 7.09738E-05 | 0.001861266 | 1.594845164 | ITM2A |
| 229533_x_at | -1.011980348 | 5.306618318 | -4.503443013 | 0.000186501 | 0.00361781 | 0.654085075 | ZNF680 |
| 226259_at | -1.01136554 | 7.787921285 | -4.563234609 | 0.000161467 | 0.00325522 | 0.794236886 | EXOC6 |
| 235142_at | -1.010901459 | 8.010301011 | -4.460262825 | 0.000206972 | 0.003850958 | 0.552864887 | ZBTB8A |
| 225061_at | -1.009428918 | 9.658770935 | -3.194398499 | 0.004281889 | 0.032401364 | -2.359998742 | DNAJA4 |
| 224452_s_at | -1.008660689 | 10.16274108 | -4.751547214 | 0.000102636 | 0.002408545 | 1.235317945 | FAM220A |
| 205992_s_at | -1.006397154 | 6.969269271 | -5.222188146 | 3.33568E-05 | 0.001081438 | 2.331685673 | IL15 |
| 204017_at | -1.003036391 | 4.905989559 | -4.374028388 | 0.000254853 | 0.004494374 | 0.350749453 | KDELR3 |
| 213851_at | -1.00293404 | 6.776830749 | -4.051457442 | 0.000555219 | 0.007731664 | -0.403599486 | TMEM110 |
| 213309_at | -1.002252629 | 8.973571158 | -5.410597131 | 2.13697E-05 | 0.000790655 | 2.766625403 | PLCL2 |
| 209527_at | 1.000452716 | 6.24118262 | 5.045585687 | 5.07677E-05 | 0.001455159 | 1.921686718 | EXOSC2 |
| 38340_at | 1.000642692 | 5.859010686 | 5.263119 | 3.02733E-05 | 0.001013356 | 2.42640696 | HIP1R |
| 200790_at | 1.000655888 | 10.42188495 | 6.119605524 | 4.12754E-06 | 0.000258752 | 4.373304206 | ODC1 |
| 203454_s_at | 1.004364649 | 6.985811595 | 4.786293717 | 9.44195E-05 | 0.002272858 | 1.316609545 | ATOX1 |
| 1560982_at | 1.00466665 | 4.564686524 | 3.250734065 | 0.003753115 | 0.029481518 | -2.235162529 | RP11-452L6.1 |
| 31845_at | 1.004824451 | 3.653834701 | 3.354084707 | 0.002943328 | 0.02502027 | -2.00435081 | ELF4 |
| 201746_at | 1.005757434 | 6.138941322 | 4.689065688 | 0.000119267 | 0.002646558 | 1.089050749 | TP53 |
| 224461_s_at | 1.006374681 | 5.778238991 | 3.627232704 | 0.00153855 | 0.015869427 | -1.384852976 | AIFM2 |
| 204995_at | 1.006915257 | 3.996536131 | 4.013926531 | 0.000607814 | 0.008287731 | -0.491048479 | CDK5R1 |
| 202288_at | 1.007094084 | 6.261338945 | 4.145898741 | 0.000442072 | 0.006657724 | -0.183180175 | MTOR |
| 201704_at | 1.007613863 | 6.094872807 | 4.059225837 | 0.000544912 | 0.007627048 | -0.385487574 | ENTPD6 |
| 201947_s_at | 1.007667599 | 10.3886709 | 7.811868383 | 1.04496E-07 | 2.42336E-05 | 7.951349516 | CCT2 |
| 201368_at | 1.010475011 | 9.639434858 | 5.084259873 | 4.62974E-05 | 0.001358745 | 2.01164174 | ZFP36L2 |
| 210719_s_at | 1.012303835 | 6.153212417 | 5.355320966 | 2.43442E-05 | 0.000874941 | 2.639312955 | HMG20B |
| 213399_x_at | 1.012803097 | 9.64822656 | 6.832429115 | 8.38325E-07 | 9.29546E-05 | 5.928650684 | RPN2 |
| 236313_at | 1.014732413 | 6.32953071 | 3.247344852 | 0.003783047 | 0.029626037 | -2.242693075 | CDKN2B |
| 209122_at | 1.015057816 | 9.651426368 | 2.964005512 | 0.007298446 | 0.047134593 | -2.862307142 | PLIN2 |
| 227668_at | 1.01677829 | 5.162826021 | 4.071454294 | 0.000529072 | 0.00748664 | -0.356969716 | ENTHD2 |
| 223035_s_at | 1.018122364 | 8.634176982 | 6.699111215 | 1.124E-06 | 0.000111402 | 5.6428545 | FARSB |
| 213353_at | 1.018835343 | 9.482793199 | 7.169415741 | 4.03654E-07 | 5.8459E-05 | 6.640055678 | ABCA5 |
| 227908_at | 1.019796867 | 6.207606076 | 6.176498534 | 3.62609E-06 | 0.000236945 | 4.499820319 | TBC1D24 |
| 204027_s_at | 1.019863226 | 6.580407044 | 3.989546308 | 0.000644607 | 0.008635495 | -0.547805031 | METTL1 |
| 200895_s_at | 1.021898527 | 8.877800104 | 4.263953048 | 0.000332436 | 0.005450779 | 0.092925922 | FKBP4 |
| 226003_at | 1.024192523 | 7.391390319 | 4.17057748 | 0.000416505 | 0.006390837 | -0.125507383 | KIF21A |
| 226966_at | 1.02437383 | 6.219049106 | 2.96685487 | 0.007250926 | 0.046948361 | -2.856183471 | PRPF40B |
| 218130_at | 1.025105622 | 5.25746526 | 3.306792338 | 0.00329019 | 0.027023735 | -2.110244556 | C17orf62 |
| 223389_s_at | 1.027930899 | 5.183687836 | 3.308022904 | 0.00328068 | 0.026976842 | -2.10749494 | ZNF581 |
| 229306_at | 1.028579086 | 4.012817246 | 2.906085505 | 0.00833169 | 0.051386239 | -2.986260062 | C2CD4B |
| 207813_s_at | 1.029204046 | 5.435954959 | 2.926504011 | 0.007952392 | 0.049817582 | -2.942678682 | FDXR |
| 227534_at | 1.029358218 | 6.819319415 | 3.790537635 | 0.001040497 | 0.011982771 | -1.009274692 | AAED1 |
| 207556_s_at | 1.02935947 | 7.797760361 | 4.998443901 | 5.68125E-05 | 0.001581036 | 1.811920325 | DGKZ |
| 202714_s_at | 1.034665869 | 4.557420199 | 2.848993098 | 0.0094863 | 0.056164339 | -3.107426861 | KIAA0391 |
| 218797_s_at | 1.035460316 | 5.963183378 | 6.971320476 | 6.19163E-07 | 7.76295E-05 | 6.223789336 | SIRT7 |
| 203835_at | 1.036533994 | 6.22873111 | 2.916222047 | 0.008141276 | 0.050572264 | -2.964640535 | LRRC32 |
| 204476_s_at | 1.03715995 | 5.083613126 | 3.318005365 | 0.003204511 | 0.026528803 | -2.085178237 | PC |
| 214434_at | 1.037453551 | 5.69889031 | 5.022830793 | 5.35996E-05 | 0.001516604 | 1.868718943 | HSPA12A |
| 205169_at | 1.037463945 | 5.989320412 | 4.630780477 | 0.000137224 | 0.002898204 | 0.952524718 | RBBP5 |
| 204900_x_at | 1.037774589 | 6.288911933 | 2.97192813 | 0.007167053 | 0.046566814 | -2.845274443 | SAP30 |
| 201074_at | 1.038506757 | 7.546777688 | 4.926290032 | 6.75066E-05 | 0.001799602 | 1.643687943 | SMARCC1 |
| 214431_at | 1.038765814 | 7.537261323 | 5.471758452 | 1.85061E-05 | 0.000722426 | 2.907190247 | GMPS |
| 224714_at | 1.038992343 | 8.432737103 | 5.801608028 | 8.57223E-06 | 0.000435136 | 3.659219772 | NIFK |
| 206688_s_at | 1.039739986 | 7.133325384 | 4.287590449 | 0.000313993 | 0.005223213 | 0.148266537 | CPSF4 |
| 1558512_at | 1.041246528 | 5.914376046 | 3.818634692 | 0.000972601 | 0.011446042 | -0.944345588 | RP11-819C21.1 |
| 210592_s_at | 1.041660331 | 8.361007113 | 4.715606272 | 0.000111894 | 0.002540132 | 1.151193986 | SAT1 |
| 229310_at | 1.04196836 | 6.674863445 | 3.047176688 | 0.006027358 | 0.041286239 | -2.682605457 | KLHL29 |
| 204175_at | 1.044012667 | 8.6144031 | 5.396728467 | 2.20794E-05 | 0.000810155 | 2.734706912 | ZNF593 |
| 201281_at | 1.046367337 | 9.767249929 | 6.692901857 | 1.13952E-06 | 0.000112416 | 5.629484369 | ADRM1 |
| 223145_s_at | 1.047262862 | 8.888535544 | 5.085709332 | 4.61378E-05 | 0.001358557 | 2.0150114 | AKIRIN2 |
| 201024_x_at | 1.047393202 | 9.260184288 | 5.734143566 | 1.00244E-05 | 0.000480007 | 3.506290197 | EIF5B |
| 213507_s_at | 1.050208214 | 9.905054677 | 6.815588557 | 8.6985E-07 | 9.4579E-05 | 5.892683581 | KPNB1 |
| 219044_at | 1.050902969 | 6.952703745 | 3.203640566 | 0.004190438 | 0.031877761 | -2.339568771 | THNSL2 |
| 226307_at | 1.052609637 | 6.033156139 | 3.76763082 | 0.001099305 | 0.012485764 | -1.062147581 | CRTC2 |
| 221984_s_at | 1.05649484 | 7.076376134 | 5.786969239 | 8.86794E-06 | 0.000445053 | 3.626077022 | FAM134A |
| 208335_s_at | 1.056843945 | 6.407050405 | 3.150255348 | 0.004746061 | 0.034910794 | -2.457299242 | ACKR1 |
| 208891_at | 1.057039392 | 5.841828642 | 2.878059376 | 0.008880594 | 0.053734861 | -3.045868821 | DUSP6 |
| 212691_at | 1.057445349 | 6.286135542 | 3.792660112 | 0.001035208 | 0.011937362 | -1.004372779 | NUP188 |
| 231901_at | 1.058072277 | 5.619690671 | 5.051140194 | 5.00997E-05 | 0.001441717 | 1.93461175 | C19orf52 |
| 225676_s_at | 1.058192454 | 8.058518981 | 6.270603198 | 2.92929E-06 | 0.000209129 | 4.708218611 | DCAF13 |
| 214663_at | 1.058955024 | 6.803910109 | 4.899009422 | 7.20615E-05 | 0.001880062 | 1.580015451 | DSTYK |
| 215089_s_at | 1.059221232 | 6.851662578 | 5.696826117 | 1.0933E-05 | 0.000506856 | 3.421495454 | RBM10 |
| 235798_at | 1.059356758 | 7.362988316 | 4.339738313 | 0.000276847 | 0.004777763 | 0.270406241 | TMEM170B |
| 204249_s_at | 1.060441203 | 9.6351266 | 3.724954104 | 0.001217763 | 0.013460642 | -1.160495778 | LMO2 |
| 223192_at | 1.061174081 | 8.198095985 | 5.414585056 | 2.11699E-05 | 0.000785722 | 2.775800531 | SLC25A28 |
| 208700_s_at | 1.062937298 | 6.882434606 | 4.847059822 | 8.16121E-05 | 0.00205811 | 1.458676033 | TKT |
| 219398_at | 1.063018261 | 6.322796678 | 3.386460661 | 0.002726724 | 0.023783094 | -1.931601553 | CIDEC |
| 222875_at | 1.064900712 | 8.06083832 | 6.293162208 | 2.78365E-06 | 0.000201305 | 4.758013241 | DHX33 |
| 201953_at | 1.065084621 | 7.57912989 | 8.403484462 | 3.16115E-08 | 1.04826E-05 | 9.105770047 | CIB1 |
| 202900_s_at | 1.065989472 | 9.259750155 | 7.569673586 | 1.72796E-07 | 3.33215E-05 | 7.464030666 | NUP88 |
| 203554_x_at | 1.067391973 | 5.584379859 | 3.390466017 | 0.002701035 | 0.023628724 | -1.922587496 | PTTG1 |
| 1557192_at | 1.067534364 | 4.748205556 | 3.796997191 | 0.001024484 | 0.011852212 | -0.994354702 | COX10-AS1 |
| 213320_at | 1.067842869 | 6.314706717 | 4.709778281 | 0.000113473 | 0.002561223 | 1.137549588 | PRMT3 |
| 208752_x_at | 1.069856826 | 11.23169998 | 6.214707685 | 3.32471E-06 | 0.00022258 | 4.58456741 | NAP1L1 |
| 226298_at | 1.070513214 | 6.449002122 | 6.960941983 | 6.33288E-07 | 7.88433E-05 | 6.201828289 | RUNDC1 |
| 212957_s_at | 1.074259827 | 5.344668626 | 4.051985335 | 0.000554512 | 0.007727891 | -0.402368827 | LINC01278 |
| 1553992_s_at | 1.074783948 | 4.85247983 | 3.712610235 | 0.001254318 | 0.013745176 | -1.188902365 | NBR2 |
| 201584_s_at | 1.075355705 | 7.291164307 | 6.018411314 | 5.20181E-06 | 0.000302575 | 4.147319913 | DDX39A |
| 218647_s_at | 1.076099617 | 5.784090642 | 3.063778395 | 0.005800531 | 0.040154468 | -2.646505606 | YRDC |
| 204247_s_at | 1.077178902 | 4.971900535 | 3.113261419 | 0.005172259 | 0.037080799 | -2.538473874 | CDK5 |
| 222116_s_at | 1.077236787 | 5.5959582 | 4.11804799 | 0.00047281 | 0.006972677 | -0.24823127 | TBC1D16 |
| 226725_at | 1.07875083 | 7.979468265 | 6.409618672 | 2.14147E-06 | 0.000173479 | 5.014045588 | SLFN5 |
| 208610_s_at | 1.079134131 | 8.304815272 | 4.209863091 | 0.000378816 | 0.006011245 | -0.033645689 | SRRM2 |
| 223168_at | 1.079170617 | 8.793895943 | 3.217606743 | 0.004055828 | 0.031203144 | -2.30865837 | RHOU |
| 203505_at | 1.080992371 | 8.890615691 | 3.296078249 | 0.003374141 | 0.027523671 | -2.134171122 | ABCA1 |
| 205111_s_at | 1.081274501 | 6.521047072 | 3.632417124 | 0.001519612 | 0.015737227 | -1.372981656 | PLCE1 |
| 203330_s_at | 1.081803744 | 5.295201166 | 3.163028503 | 0.004606959 | 0.034183209 | -2.429192841 | STX5 |
| 229689_s_at | 1.083991832 | 6.763856513 | 5.520012514 | 1.65245E-05 | 0.000671048 | 3.017857474 | RP13-39P12.3 |
| 1558953_s_at | 1.084499206 | 5.608152079 | 4.210974106 | 0.000377801 | 0.005997824 | -0.031046915 | CEP164 |
| 1566480_x_at | 1.084764015 | 5.499932252 | 3.979769289 | 0.000659976 | 0.008750848 | -0.570553829 | C17orf104 |
| 221485_at | 1.085103237 | 9.536272688 | 5.251815908 | 3.10947E-05 | 0.001035014 | 2.400261884 | B4GALT5 |
| 203938_s_at | 1.086060318 | 5.127050768 | 2.918935208 | 0.008091021 | 0.050421357 | -2.958848469 | TAF1C |
| 212163_at | 1.08685019 | 7.416935463 | 5.336032983 | 2.54784E-05 | 0.000902194 | 2.594829817 | KIDINS220 |
| 225328_at | 1.088274879 | 11.8196548 | 6.772775916 | 9.55594E-07 | 0.000100612 | 5.801071332 | FBXO32 |
| 222258_s_at | 1.089694989 | 6.942507145 | 3.279444578 | 0.003508623 | 0.02824891 | -2.171269303 | SH3BP4 |
| 218847_at | 1.090597118 | 4.33037846 | 3.393863015 | 0.002679432 | 0.023490595 | -1.914940181 | IGF2BP2 |
| 209441_at | 1.092028708 | 5.950716854 | 3.605756109 | 0.00161951 | 0.016476802 | -1.433990355 | RHOBTB2 |
| 205702_at | 1.093283374 | 5.034338243 | 4.599539846 | 0.000147945 | 0.003059081 | 0.879322433 | PHTF1 |
| 204881_s_at | 1.096151277 | 7.712122871 | 5.083310832 | 4.64021E-05 | 0.001360695 | 2.009435368 | UGCG |
| 212717_at | 1.097566248 | 7.385645773 | 4.177171919 | 0.000409927 | 0.006323919 | -0.110092001 | PLEKHM1 |
| 217995_at | 1.098576475 | 10.07436626 | 6.097146817 | 4.34453E-06 | 0.000268683 | 4.323254514 | SQRDL |
| 229268_at | 1.101451056 | 5.469696593 | 3.63832135 | 0.001498323 | 0.015604198 | -1.35945763 | OTULIN |
| 213571_s_at | 1.103743428 | 8.165701211 | 4.543813834 | 0.000169204 | 0.003378565 | 0.748716799 | EIF4E2 |
| 222199_s_at | 1.103786716 | 6.214200932 | 6.815644996 | 8.69743E-07 | 9.4579E-05 | 5.892804186 | BIN3 |
| 204257_at | 1.104154733 | 7.58440845 | 4.036859755 | 0.000575115 | 0.00794635 | -0.437623667 | FADS3 |
| 203727_at | 1.10476148 | 6.784311905 | 7.522956502 | 1.90573E-07 | 3.50666E-05 | 7.369050216 | SKIV2L |
| 224721_at | 1.10496293 | 7.157727381 | 5.838445338 | 7.8718E-06 | 0.000407752 | 3.742519397 | WDR75 |
| 220009_at | 1.105650066 | 5.893106252 | 4.986832052 | 5.84101E-05 | 0.001615062 | 1.784864329 | LONRF3 |
| 243648_at | 1.108811941 | 4.835734004 | 4.724484004 | 0.000109532 | 0.002502513 | 1.171976676 | ZBED6 |
| 209295_at | 1.10950511 | 6.758313867 | 5.237417358 | 3.21739E-05 | 0.001060961 | 2.366943079 | TNFRSF10B |
| 227099_s_at | 1.109647628 | 7.555313234 | 3.152969507 | 0.004716167 | 0.034796124 | -2.451330264 | C11orf96 |
| 221712_s_at | 1.110145596 | 7.573359876 | 6.568148585 | 1.50258E-06 | 0.000136007 | 5.359764094 | WDR74 |
| 200605_s_at | 1.110812088 | 11.07767128 | 4.99753173 | 5.69364E-05 | 0.001582002 | 1.809795195 | PRKAR1A |
| 229049_at | 1.111208607 | 2.965458475 | 2.865637136 | 0.009134795 | 0.054768637 | -3.072210426 | LOC100506655 |
| 203617_x_at | 1.114955968 | 6.947230283 | 3.802894905 | 0.001010076 | 0.011754497 | -0.980728584 | ELK1 |
| 200657_at | 1.116176143 | 8.925093885 | 4.090556574 | 0.000505241 | 0.007284427 | -0.312404072 | SLC25A5 |
| 230925_at | 1.116938617 | 4.881736219 | 3.260511916 | 0.003668044 | 0.029028532 | -2.213422777 | APBB1IP |
| 227345_at | 1.118744297 | 4.517192355 | 3.279917822 | 0.003504725 | 0.028223937 | -2.170214637 | TNFRSF10D |
| 210652_s_at | 1.119028675 | 4.988226187 | 2.941470838 | 0.007684933 | 0.048753228 | -2.910652858 | TTC39A |
| 225609_at | 1.122356845 | 7.00701145 | 5.78936541 | 8.81884E-06 | 0.000444474 | 3.631503598 | GSR |
| 217893_s_at | 1.122424465 | 7.00863695 | 6.375767002 | 2.31072E-06 | 0.000179063 | 4.939799931 | AKIRIN1 |
| 235117_at | 1.123870594 | 6.505571396 | 4.08683967 | 0.000509793 | 0.007322797 | -0.321077236 | CHAC2 |
| 218481_at | 1.126936861 | 7.516145598 | 4.75397019 | 0.00010204 | 0.002404107 | 1.240987867 | EXOSC5 |
| 207469_s_at | 1.128410147 | 5.720415261 | 4.075744342 | 0.000523624 | 0.007437282 | -0.34696285 | PIR |
| 206335_at | 1.12856668 | 5.020724959 | 3.137659174 | 0.004887214 | 0.035664429 | -2.484976989 | GALNS |
| 51158_at | 1.129840798 | 6.7945124 | 4.313046949 | 0.000295275 | 0.005008566 | 0.207882509 | FAM174B |
| 219525_at | 1.1299378 | 9.102573356 | 4.090231283 | 0.000505638 | 0.007287186 | -0.313163151 | SLC47A1 |
| 218547_at | 1.130098769 | 5.981861827 | 6.349597402 | 2.45091E-06 | 0.000183627 | 4.882302647 | DHDDS |
| 202054_s_at | 1.131462204 | 9.492933836 | 3.446601532 | 0.002364951 | 0.021519451 | -1.795942749 | ALDH3A2 |
| 218999_at | 1.131874008 | 8.421489538 | 3.843378075 | 0.000916452 | 0.010981954 | -0.887099912 | TMEM140 |
| 231179_at | 1.133413795 | 9.664106985 | 4.078417749 | 0.000520258 | 0.007413572 | -0.340726383 | IP6K3 |
| 212978_at | 1.134542103 | 6.031453845 | 4.161584708 | 0.000425646 | 0.00649302 | -0.146526144 | LRRC8B |
| 205427_at | 1.136846849 | 5.531723352 | 3.340392696 | 0.003039904 | 0.025577218 | -2.035055343 | ZNF354A |
| 209556_at | 1.136892975 | 5.08304974 | 3.881993491 | 0.000835178 | 0.010304831 | -0.797642484 | NCDN |
| 200766_at | 1.138641391 | 7.755253062 | 5.169272702 | 3.78206E-05 | 0.001180257 | 2.209052733 | CTSD |
| 213435_at | 1.139660922 | 5.729136296 | 5.06174621 | 4.88488E-05 | 0.001414901 | 1.959286376 | SATB2 |
| 208696_at | 1.140637642 | 9.394934495 | 6.476013103 | 1.84545E-06 | 0.000156492 | 5.159236741 | CCT5 |
| 228031_at | 1.141213256 | 4.950157565 | 3.393455255 | 0.002682016 | 0.023498123 | -1.915858244 | TTPAL |
| 203368_at | 1.142667825 | 6.285191776 | 6.638173249 | 1.28621E-06 | 0.000123011 | 5.511416406 | CRELD1 |
| 201277_s_at | 1.143348236 | 10.16048174 | 6.332500983 | 2.54717E-06 | 0.000188289 | 4.844693038 | HNRNPAB |
| 1553689_s_at | 1.144100308 | 4.515753495 | 3.372778755 | 0.002816291 | 0.02427785 | -1.962369676 | METTL6 |
| 201948_at | 1.145835084 | 8.374626912 | 9.821976665 | 2.16333E-09 | 2.01998E-06 | 11.66922606 | GNL2 |
| 201714_at | 1.14719762 | 8.411647223 | 8.48964657 | 2.66626E-08 | 9.55598E-06 | 9.269648349 | TUBG1 |
| 217817_at | 1.149096452 | 7.339169831 | 5.26534275 | 3.01143E-05 | 0.001009607 | 2.431549575 | ARPC4 |
| 220353_at | 1.151251423 | 4.209812555 | 2.919101306 | 0.008087954 | 0.050417568 | -2.958493808 | FAM86C1 |
| 236269_at | 1.153741885 | 5.663882095 | 4.935083446 | 6.61013E-05 | 0.001775479 | 1.664204258 | ZNF628 |
| 242662_at | 1.15416831 | 5.919977213 | 3.30848058 | 0.003277149 | 0.026960307 | -2.106472214 | PCSK6 |
| 215170_s_at | 1.156933539 | 3.509273399 | 3.004615992 | 0.006648577 | 0.044235905 | -2.774807389 | CEP152 |
| 1552856_at | 1.157797668 | 3.630740771 | 2.902363983 | 0.008402663 | 0.051689195 | -2.99418948 | LINC00311 |
| 229572_at | 1.158051374 | 6.366091675 | 6.228437132 | 3.22278E-06 | 0.000219224 | 4.61497529 | ATP6V0A2 |
| 201590_x_at | 1.158342892 | 10.37677639 | 5.079075004 | 4.68727E-05 | 0.001373359 | 1.999587063 | ANXA2 |
| 215544_s_at | 1.1587125 | 6.109073215 | 3.811617365 | 0.000989135 | 0.011589386 | -0.960569503 | UBOX5 |
| 218305_at | 1.159096506 | 7.291049196 | 6.749097725 | 1.0067E-06 | 0.00010475 | 5.750295989 | IPO4 |
| 214662_at | 1.164901537 | 7.867926483 | 7.091809829 | 4.77011E-07 | 6.53486E-05 | 6.477639487 | WDR43 |
| 224654_at | 1.167008756 | 9.452639412 | 5.352071071 | 2.45316E-05 | 0.000877451 | 2.631819946 | DDX21 |
| 225611_at | 1.169929107 | 7.630725423 | 4.423403523 | 0.000226222 | 0.004089052 | 0.466466481 | MAST4 |
| 227116_at | 1.170156019 | 6.201075383 | 5.532356115 | 1.60531E-05 | 0.000656976 | 3.046132649 | MON1B |
| 204133_at | 1.172918626 | 5.618329657 | 3.817325288 | 0.000975665 | 0.011463394 | -0.947373284 | RRP9 |
| 212473_s_at | 1.173323997 | 8.381361281 | 3.105361827 | 0.005267948 | 0.037533604 | -2.555762964 | MICAL2 |
| 202200_s_at | 1.17575316 | 7.820260668 | 6.827114465 | 8.48145E-07 | 9.317E-05 | 5.917304177 | SRPK1 |
| 200634_at | 1.176731797 | 9.431560535 | 7.620853446 | 1.55271E-07 | 3.14819E-05 | 7.567720695 | PFN1 |
| 230302_at | 1.17846792 | 5.173692661 | 4.521955175 | 0.000178359 | 0.003510648 | 0.697479596 | RP11-48B3.4 |
| 225814_at | 1.178896042 | 8.841520187 | 4.782425244 | 9.53004E-05 | 0.00229073 | 1.307560931 | XRN1 |
| 201589_at | 1.180354548 | 6.881887445 | 4.740390029 | 0.000105424 | 0.002448855 | 1.209207158 | SMC1A |
| 201872_s_at | 1.183770859 | 8.980908685 | 6.174353106 | 3.64382E-06 | 0.000237666 | 4.495056519 | ABCE1 |
| 213302_at | 1.186971546 | 6.05342776 | 4.801865002 | 9.0956E-05 | 0.002210124 | 1.353026683 | PFAS |
| 222162_s_at | 1.187764192 | 7.59039195 | 3.842011065 | 0.000919469 | 0.010999527 | -0.890264169 | ADAMTS1 |
| 228132_at | 1.194012817 | 9.692448982 | 4.746832323 | 0.000103805 | 0.002426352 | 1.224284289 | ABLIM2 |
| 222754_at | 1.194153003 | 7.031366042 | 5.164314738 | 3.82688E-05 | 0.001189013 | 2.19755264 | TRNT1 |
| 236468_at | 1.196426737 | 4.625769011 | 2.955755514 | 0.007437724 | 0.047696203 | -2.880024084 | TMEM132B |
| 219952_s_at | 1.201328273 | 6.062181956 | 4.562026195 | 0.000161938 | 0.003261007 | 0.791404592 | MCOLN1 |
| 226938_at | 1.201481173 | 6.33117702 | 4.608210416 | 0.000144888 | 0.003011794 | 0.899640547 | DCAF4 |
| 223402_at | 1.20391526 | 7.12543364 | 4.512004132 | 0.00018269 | 0.003571266 | 0.674153395 | DUSP23 |
| 201360_at | 1.204121879 | 10.04031686 | 5.359507045 | 2.41049E-05 | 0.000869196 | 2.64896318 | CST3 |
| 226796_at | 1.20620173 | 3.915261792 | 2.989370717 | 0.006885736 | 0.045328329 | -2.807710641 | ABHD15 |
| 225726_s_at | 1.210599114 | 6.38958066 | 3.899292084 | 0.000801127 | 0.010042147 | -0.757523893 | PLEKHH1 |
| 219417_s_at | 1.21376841 | 4.954859618 | 3.474045306 | 0.002215903 | 0.02056084 | -1.733823718 | C17orf59 |
| 1556082_a_at | 1.214548318 | 5.656169733 | 4.68004028 | 0.000121885 | 0.002681163 | 1.067914447 | RP11-16P6.1 |
| 218052_s_at | 1.21875088 | 6.383296074 | 7.922498026 | 8.32647E-08 | 2.0508E-05 | 8.171102083 | ATP13A1 |
| 226168_at | 1.220483096 | 6.059204354 | 4.637188173 | 0.000135124 | 0.002869214 | 0.967537161 | ZFAND2B |
| 225503_at | 1.22074472 | 5.676033813 | 3.324663061 | 0.003154672 | 0.026232497 | -2.070282973 | DHRSX |
| 228702_at | 1.22187105 | 8.50997778 | 6.563865291 | 1.51697E-06 | 0.000136612 | 5.350466581 | LINC-PINT |
| 244828_x_at | 1.227468633 | 6.779172765 | 4.205261284 | 0.000383048 | 0.006056738 | -0.044409274 | NAF1 |
| 209286_at | 1.228270437 | 9.040048535 | 3.605665452 | 0.00161986 | 0.016476802 | -1.434197635 | CDC42EP3 |
| 218007_s_at | 1.228598023 | 11.1494297 | 8.073121486 | 6.12781E-08 | 1.65975E-05 | 8.467425759 | RPS27L |
| 219353_at | 1.231572898 | 7.550967991 | 4.56894165 | 0.000159262 | 0.003231367 | 0.807612933 | NHLRC2 |
| 218189_s_at | 1.231586131 | 7.494183077 | 6.315011492 | 2.64966E-06 | 0.000193846 | 4.806180444 | NANS |
| 211316_x_at | 1.235099465 | 10.01080465 | 6.304984081 | 2.71031E-06 | 0.000197469 | 4.78408226 | CFLAR |
| 205902_at | 1.235335407 | 7.814409553 | 4.233608206 | 0.000357708 | 0.005760568 | 0.021906221 | KCNN3 |
| 200957_s_at | 1.236020591 | 8.357201878 | 7.10772431 | 4.60923E-07 | 6.37851E-05 | 6.511015448 | SSRP1 |
| 1554063_at | 1.238137239 | 3.286747492 | 2.954734613 | 0.007455136 | 0.047756476 | -2.882215081 | ZHX1-C8orf76 |
| 203782_s_at | 1.238314364 | 8.503712325 | 7.727684788 | 1.24348E-07 | 2.74044E-05 | 7.782932704 | POLRMT |
| 221640_s_at | 1.239310068 | 4.398793802 | 3.631378859 | 0.001523386 | 0.015754233 | -1.375359379 | PIDD1 |
| 210788_s_at | 1.241916935 | 11.50122343 | 7.837553062 | 9.91143E-08 | 2.32899E-05 | 8.002528247 | DHRS7 |
| 203574_at | 1.254299073 | 8.373966767 | 3.269091699 | 0.003594944 | 0.028690014 | -2.194329548 | NFIL3 |
| 204170_s_at | 1.257513675 | 5.765991875 | 3.347572108 | 0.002988885 | 0.025244376 | -2.018960043 | CKS2 |
| 1557458_s_at | 1.260175426 | 5.358922198 | 3.340996414 | 0.003035582 | 0.025553621 | -2.033702281 | SHB |
| 223852_s_at | 1.26057663 | 8.283192727 | 5.348162227 | 2.4759E-05 | 0.000882914 | 2.622806526 | STK40 |
| 214438_at | 1.261691009 | 5.135463529 | 3.929706157 | 0.000744567 | 0.009547793 | -0.6869254 | HLX |
| 232196_at | 1.261739544 | 4.591909047 | 2.977503762 | 0.007075949 | 0.046125364 | -2.833276513 | LCA5L |
| 228050_at | 1.263292966 | 5.197647561 | 4.018223929 | 0.000601549 | 0.00822511 | -0.481039998 | UTP15 |
| 208794_s_at | 1.272904964 | 8.451961267 | 7.912178811 | 8.50417E-08 | 2.06675E-05 | 8.150679729 | SMARCA4 |
| 202180_s_at | 1.278034837 | 6.672245394 | 4.283315681 | 0.000317251 | 0.005260143 | 0.138257173 | MVP |
| 203018_s_at | 1.279747329 | 5.996412908 | 5.708753137 | 1.06338E-05 | 0.00049905 | 3.448612161 | SSX2IP |
| 225104_at | 1.280100395 | 5.671734422 | 7.779265811 | 1.11765E-07 | 2.55848E-05 | 7.886247746 | ZNF598 |
| 54632_at | 1.281345172 | 6.749469623 | 6.474333622 | 1.85239E-06 | 0.000156492 | 5.155571115 | THADA |
| 1569003_at | 1.281802015 | 4.67505218 | 3.517702231 | 0.00199757 | 0.019150723 | -1.634743934 | VMP1 |
| 204488_at | 1.283717367 | 6.033367989 | 5.356631477 | 2.4269E-05 | 0.000873339 | 2.642334245 | DOLK |
| 209820_s_at | 1.285040209 | 7.011699605 | 6.868996027 | 7.7385E-07 | 8.85734E-05 | 6.006613519 | TBL3 |
| 214467_at | 1.285434494 | 2.590256223 | 3.074668107 | 0.005656242 | 0.039477132 | -2.622786201 | GPR65 |
| 208623_s_at | 1.286134401 | 7.681388375 | 4.848453907 | 8.13398E-05 | 0.002056213 | 1.461933668 | EZR |
| 218670_at | 1.290967773 | 5.976493173 | 4.629737701 | 0.000137569 | 0.002903761 | 0.950081557 | PUS1 |
| 209208_at | 1.298140097 | 6.44718345 | 5.439658534 | 1.99567E-05 | 0.000759707 | 2.83345662 | MPDU1 |
| 202212_at | 1.299759552 | 7.193842889 | 7.411592564 | 2.40963E-07 | 4.03295E-05 | 7.141361426 | PES1 |
| 218556_at | 1.301645973 | 7.253636632 | 9.833488474 | 2.11893E-09 | 2.01998E-06 | 11.68888519 | ORMDL2 |
| 1559617_at | 1.302495662 | 4.106278187 | 3.461463355 | 0.002283057 | 0.021002704 | -1.762319164 | LOC101927206 |
| 222641_s_at | 1.305436846 | 8.432432136 | 5.296664776 | 2.79628E-05 | 0.000959096 | 2.503944801 | FAM222B |
| 241824_at | 1.308534112 | 4.999809637 | 3.061903137 | 0.005825734 | 0.04024702 | -2.650587005 | RP11-373D23.2 |
| 204559_s_at | 1.309298766 | 8.72425026 | 5.013156161 | 5.48516E-05 | 0.001542191 | 1.846190001 | LSM7 |
| 219481_at | 1.30998632 | 7.252852016 | 7.459512108 | 2.17781E-07 | 3.733E-05 | 7.239555047 | TTC13 |
| 212680_x_at | 1.311853795 | 8.303185871 | 6.140458452 | 3.93599E-06 | 0.00025093 | 4.419721335 | PPP1R14B |
| 218444_at | 1.313618723 | 4.896051073 | 2.910310096 | 0.008251819 | 0.051035567 | -2.977253569 | ALG12 |
| 201587_s_at | 1.314944019 | 8.824143795 | 11.13753713 | 2.23275E-10 | 6.25109E-07 | 13.80363059 | IRAK1 |
| 222768_s_at | 1.315183483 | 7.326430522 | 6.193583039 | 3.48802E-06 | 0.000230899 | 4.537735461 | TRMT6 |
| 1552427_at | 1.319182269 | 4.850701765 | 2.973394081 | 0.007142991 | 0.046453008 | -2.842120812 | ZNF485 |
| 1566785_x_at | 1.322955118 | 6.530087296 | 5.562981956 | 1.4942E-05 | 0.000619359 | 3.116225508 | NSF |
| 218196_at | 1.325895576 | 7.401894024 | 9.324601736 | 5.38256E-09 | 3.47244E-06 | 10.80255292 | OSTM1 |
| 210627_s_at | 1.326063809 | 5.825597068 | 7.385417484 | 2.54685E-07 | 4.18367E-05 | 7.087585157 | MOGS |
| 217770_at | 1.326450378 | 7.62419882 | 5.09113731 | 4.55452E-05 | 0.001343337 | 2.027629068 | PIGT |
| 242260_at | 1.328996781 | 6.405879505 | 6.25594456 | 3.02808E-06 | 0.000212485 | 4.675828471 | MATR3 |
| 204866_at | 1.330764086 | 4.440297312 | 4.212083892 | 0.00037679 | 0.005984452 | -0.028450971 | JADE3 |
| 202017_at | 1.333386929 | 9.75287573 | 4.679156437 | 0.000122144 | 0.00268492 | 1.065844507 | EPHX1 |
| 207643_s_at | 1.333575626 | 8.308975395 | 8.479656984 | 2.71928E-08 | 9.64854E-06 | 9.250703527 | TNFRSF1A |
| 213436_at | 1.336736394 | 3.983280327 | 3.436946181 | 0.00241969 | 0.021857295 | -1.817766343 | CNR1 |
| 203904_x_at | 1.336808685 | 6.597126588 | 3.426757119 | 0.0024788 | 0.022215908 | -1.840778241 | CD82 |
| 225059_at | 1.340326658 | 7.17945569 | 5.763785453 | 9.35772E-06 | 0.000461154 | 3.573541726 | AGTRAP |
| 1552306_at | 1.342325814 | 2.961344367 | 2.9251388 | 0.007977228 | 0.049915998 | -2.945596571 | ALG10 |
| 235857_at | 1.34438916 | 4.440798042 | 4.322081892 | 0.000288904 | 0.0049296 | 0.229044904 | KCTD11 |
| 1560089_at | 1.345235618 | 5.495120115 | 3.773789901 | 0.001083177 | 0.012342097 | -1.047936989 | LOC100289019 |
| 217027_x_at | 1.347410506 | 5.758524615 | 3.986236191 | 0.00064977 | 0.008673866 | -0.555507663 | AC004941.5 |
| 203737_s_at | 1.348176319 | 6.979874639 | 7.086860561 | 4.8213E-07 | 6.5796E-05 | 6.467252541 | PPRC1 |
| 219215_s_at | 1.355134231 | 5.426900464 | 4.684992819 | 0.000120441 | 0.002662174 | 1.079512862 | SLC39A4 |
| 1562251_a_at | 1.355252335 | 2.439967545 | 3.268398696 | 0.003600795 | 0.028710881 | -2.195872333 | LOC574538 |
| 201703_s_at | 1.357205918 | 5.165464373 | 3.108367211 | 0.005231344 | 0.03733277 | -2.549187247 | PPP1R10 |
| 212971_at | 1.357219061 | 9.648192779 | 8.39267749 | 3.2296E-08 | 1.05131E-05 | 9.085139449 | CARS |
| 49452_at | 1.362018037 | 9.950722142 | 4.169403487 | 0.000417687 | 0.006402763 | -0.128251552 | ACACB |
| 216913_s_at | 1.365008862 | 7.131646193 | 4.492938617 | 0.000191286 | 0.003660838 | 0.629461294 | RRP12 |
| 223063_at | 1.367075353 | 9.290036048 | 3.569517064 | 0.001765726 | 0.017529794 | -1.516753373 | C1orf198 |
| 209326_at | 1.36897778 | 5.499371844 | 4.631954608 | 0.000136837 | 0.002894047 | 0.955275615 | SLC35A2 |
| 1560306_at | 1.374351024 | 4.46669813 | 4.521879521 | 0.000178392 | 0.003510648 | 0.697302257 | RP11-68I3.11 |
| 204458_at | 1.382195935 | 6.735555278 | 4.060296804 | 0.000543506 | 0.007616383 | -0.38299034 | PLA2G15 |
| 203334_at | 1.387158444 | 6.365118616 | 5.276244865 | 2.9347E-05 | 0.000991706 | 2.456756296 | DHX8 |
| 207992_s_at | 1.395488142 | 8.666270179 | 3.899302487 | 0.000801107 | 0.010042147 | -0.757499759 | AMPD3 |
| 224634_at | 1.397080264 | 8.709976388 | 7.845250673 | 9.75577E-08 | 2.30769E-05 | 8.017847593 | GPATCH4 |
| 226178_at | 1.397304544 | 7.384166462 | 6.027500875 | 5.09459E-06 | 0.000297803 | 4.167667602 | SOCS4 |
| 224762_at | 1.406064414 | 7.012190041 | 4.939868431 | 6.53491E-05 | 0.001763283 | 1.675366776 | SERINC2 |
| 212367_at | 1.407060447 | 8.482793112 | 7.037005244 | 5.36968E-07 | 7.10922E-05 | 6.362429081 | FEM1B |
| 223375_at | 1.408694672 | 5.841300516 | 3.889988856 | 0.000819265 | 0.010178279 | -0.779103073 | TBC1D22B |
| 243815_at | 1.414393977 | 3.733735368 | 3.85415668 | 0.000893009 | 0.010806678 | -0.862144054 | PGBD4 |
| 224792_at | 1.422700413 | 6.983370229 | 4.991012008 | 5.78299E-05 | 0.001603062 | 1.794604589 | TNKS1BP1 |
| 226516_at | 1.425190735 | 4.689464792 | 3.426775863 | 0.00247869 | 0.022215908 | -1.840735926 | MFSD12 |
| 1555783_x_at | 1.426390108 | 5.131037497 | 3.744444129 | 0.001162176 | 0.013028858 | -1.115607159 | PQLC2 |
| 231779_at | 1.427195816 | 6.611732838 | 6.268001707 | 2.94658E-06 | 0.00020961 | 4.702472236 | IRAK2 |
| 216941_s_at | 1.431991047 | 5.005996572 | 4.347182473 | 0.000271916 | 0.004719717 | 0.287846516 | TAF1B |
| 242585_at | 1.43374529 | 4.831623464 | 3.130133368 | 0.004973479 | 0.036109878 | -2.501494753 | CKMT2-AS1 |
| 202912_at | 1.435821528 | 7.208473011 | 6.690844398 | 1.14471E-06 | 0.000112512 | 5.62505305 | ADM |
| 224840_at | 1.436779659 | 11.8923196 | 4.600032652 | 0.000147769 | 0.003058427 | 0.880477276 | FKBP5 |
| 203973_s_at | 1.436924356 | 9.939513172 | 4.314632865 | 0.000294146 | 0.004996621 | 0.211597042 | CEBPD |
| 242922_at | 1.441383129 | 5.029248884 | 3.218604073 | 0.004046378 | 0.031153783 | -2.306449331 | NOMO3 |
| 229622_at | 1.444066574 | 5.796817807 | 3.330783416 | 0.003109523 | 0.025954383 | -2.056581943 | FAM132B |
| 212099_at | 1.444512956 | 9.86210918 | 4.106008895 | 0.000486749 | 0.007095658 | -0.276338907 | RHOB |
| 202193_at | 1.445014068 | 5.732266759 | 5.028020906 | 5.29399E-05 | 0.001505942 | 1.880802817 | LIMK2 |
| 230860_at | 1.447499449 | 3.810378903 | 3.913210283 | 0.000774734 | 0.009819479 | -0.725226067 | CEP19 |
| 224327_s_at | 1.451733681 | 5.10652526 | 3.580039552 | 0.001721982 | 0.017201398 | -1.492741885 | DGAT2 |
| 203701_s_at | 1.452271853 | 6.632971958 | 6.438532205 | 2.007E-06 | 0.000165226 | 5.077343831 | TRMT1 |
| 221823_at | 1.454063575 | 7.500149338 | 4.203162946 | 0.000384994 | 0.006076669 | -0.049317001 | C5orf30 |
| 202779_s_at | 1.45867048 | 6.191556482 | 5.609534527 | 1.34008E-05 | 0.000571501 | 3.222600018 | UBE2S |
| 209448_at | 1.460545038 | 8.281253778 | 5.338630641 | 2.53226E-05 | 0.000898522 | 2.600822437 | HTATIP2 |
| 201850_at | 1.460657854 | 4.585871756 | 3.02251942 | 0.006380092 | 0.042980525 | -2.736083309 | CAPG |
| 1559116_s_at | 1.462435419 | 2.164141438 | 2.953741559 | 0.00747211 | 0.047847932 | -2.884346019 | RP5-1065J22.8 |
| 227477_at | 1.465008388 | 5.519907326 | 4.087441311 | 0.000509053 | 0.007315608 | -0.319673396 | ZMYND19 |
| 237335_at | 1.4660719 | 2.756844217 | 2.843997173 | 0.009594303 | 0.056567806 | -3.117980028 | ZP1 |
| 201797_s_at | 1.466527077 | 7.743143301 | 9.010195605 | 9.73E-09 | 5.23091E-06 | 10.23697079 | VARS |
| 55081_at | 1.469265017 | 5.78240987 | 5.3263843 | 2.60658E-05 | 0.000913694 | 2.572566254 | MICALL1 |
| 218368_s_at | 1.470069305 | 6.850061883 | 3.510704497 | 0.002031088 | 0.019383288 | -1.65064644 | TNFRSF12A |
| 202852_s_at | 1.471292993 | 6.668233238 | 8.697686413 | 1.77463E-08 | 7.32179E-06 | 9.660910028 | AAGAB |
| 229811_at | 1.476476684 | 3.601349988 | 4.345790203 | 0.000272831 | 0.004729166 | 0.284584608 | RP11-157P1.4 |
| 213119_at | 1.486214677 | 5.552222928 | 6.083538234 | 4.48166E-06 | 0.000272291 | 4.2928983 | SLC36A1 |
| 244834_at | 1.491448059 | 4.630594768 | 3.831446426 | 0.000943115 | 0.011191846 | -0.914712268 | RSG1 |
| 228108_at | 1.494233969 | 8.629916563 | 5.132585452 | 4.12669E-05 | 0.001253671 | 2.123917218 | PPM1L |
| 202679_at | 1.495533129 | 7.128604652 | 7.332492735 | 2.84943E-07 | 4.47361E-05 | 6.978550548 | NPC1 |
| 203313_s_at | 1.503085852 | 6.159970978 | 5.714535412 | 1.04918E-05 | 0.000495699 | 3.46175325 | TGIF1 |
| 218860_at | 1.506097632 | 5.466734715 | 3.812292051 | 0.000987534 | 0.011575709 | -0.959009862 | NOC4L |
| 228587_at | 1.507433142 | 4.841162281 | 3.794717485 | 0.001030107 | 0.011898805 | -0.999620769 | FAM83G |
| 228724_at | 1.508381554 | 5.736429931 | 4.417199544 | 0.000229634 | 0.004138084 | 0.451925236 | TTLL7 |
| 202724_s_at | 1.509413411 | 9.052929408 | 3.524090673 | 0.001967444 | 0.018933782 | -1.620219181 | FOXO1 |
| 201348_at | 1.509687594 | 11.01248485 | 6.937931845 | 6.65798E-07 | 8.09036E-05 | 6.153084818 | GPX3 |
| 201473_at | 1.512527278 | 5.920566243 | 3.249951288 | 0.003760007 | 0.029505612 | -2.236902019 | JUNB |
| 212996_s_at | 1.51412404 | 5.724105504 | 5.283335184 | 2.88587E-05 | 0.000978067 | 2.473144964 | URB1 |
| 225779_at | 1.514973355 | 6.174636072 | 4.417841267 | 0.000229279 | 0.004135797 | 0.453429328 | SLC27A4 |
| 225617_at | 1.518155661 | 4.873279386 | 4.417639111 | 0.000229391 | 0.004135797 | 0.452955507 | ODF2 |
| 223805_at | 1.520191585 | 9.321001654 | 4.427568864 | 0.00022396 | 0.004071601 | 0.476229626 | OSBPL6 |
| 213192_at | 1.523221109 | 4.466475659 | 3.464306564 | 0.00226771 | 0.020919173 | -1.755882278 | THAP3 |
| 240967_at | 1.523315679 | 4.118899263 | 2.835869763 | 0.009772514 | 0.057442637 | -3.135130583 | KRTAP19-3 |
| 213523_at | 1.524000063 | 5.109417272 | 4.260176716 | 0.000335481 | 0.005480448 | 0.084086146 | CCNE1 |
| 210605_s_at | 1.525025143 | 8.004522069 | 4.168598703 | 0.000418499 | 0.006411568 | -0.130132672 | MFGE8 |
| 209837_at | 1.525966806 | 5.399741172 | 4.71996781 | 0.000110727 | 0.002521707 | 1.161404568 | AP4M1 |
| 201577_at | 1.527414069 | 8.439615101 | 7.594224676 | 1.64148E-07 | 3.25381E-05 | 7.513818487 | NME1 |
| 208433_s_at | 1.52875907 | 4.044694132 | 2.94576403 | 0.007609819 | 0.048423889 | -2.901453915 | LRP8 |
| 224918_x_at | 1.530437625 | 7.294996178 | 5.031432282 | 5.25108E-05 | 0.001495335 | 1.888744525 | MGST1 |
| 202693_s_at | 1.53128849 | 6.960917575 | 5.412610655 | 2.12686E-05 | 0.000788559 | 2.771258142 | STK17A |
| 202308_at | 1.531836927 | 6.54231234 | 3.551756877 | 0.001842049 | 0.018067425 | -1.55724293 | SREBF1 |
| 213927_at | 1.533484058 | 4.869732082 | 4.443101762 | 0.000215721 | 0.003959758 | 0.512638288 | MAP3K9 |
| 227964_at | 1.536009103 | 6.115664393 | 6.548723634 | 1.56899E-06 | 0.000140263 | 5.317579926 | FRMD8 |
| 206819_at | 1.536628267 | 2.82690935 | 3.085296744 | 0.005518755 | 0.038873385 | -2.599605197 | POM121L9P |
| 204862_s_at | 1.536983972 | 4.746479355 | 4.006727922 | 0.000618455 | 0.008398016 | -0.507810964 | NME3 |
| 205115_s_at | 1.537177844 | 6.032875743 | 5.160873979 | 3.85829E-05 | 0.001196677 | 2.189570761 | RBM19 |
| 202370_s_at | 1.537430993 | 9.15142949 | 8.857067254 | 1.30412E-08 | 6.25302E-06 | 9.95644639 | CBFB |
| 227060_at | 1.540691668 | 5.991482284 | 3.340115255 | 0.003041893 | 0.025582472 | -2.035677121 | RELT |
| 226982_at | 1.540937218 | 7.389508802 | 4.897854719 | 7.2261E-05 | 0.001882499 | 1.577319651 | ELL2 |
| 228479_at | 1.541945751 | 6.518987565 | 4.911814676 | 6.9886E-05 | 0.00184364 | 1.60990694 | SOAT1 |
| 1566257_at | 1.54237921 | 6.047685777 | 7.713499309 | 1.28058E-07 | 2.78758E-05 | 7.75445169 | GPR180 |
| 224753_at | 1.54389677 | 3.839749627 | 4.744745041 | 0.000104327 | 0.00243374 | 1.219399479 | CDCA5 |
| 218564_at | 1.543976837 | 3.929782711 | 3.943645426 | 0.000719988 | 0.009316777 | -0.654543332 | RFWD3 |
| 218938_at | 1.544519326 | 5.413018379 | 4.046530072 | 0.000561857 | 0.007796561 | -0.415085659 | FBXL15 |
| 213449_at | 1.551812705 | 4.021143858 | 3.998113241 | 0.000631432 | 0.008502652 | -0.527866169 | POP1 |
| 243309_at | 1.554896331 | 6.409190357 | 8.632742123 | 2.01389E-08 | 7.91394E-06 | 9.539439213 | C15orf65 |
| 1562921_at | 1.556678328 | 5.029075215 | 3.596353764 | 0.00165626 | 0.016757172 | -1.455481877 | EP300-AS1 |
| 219715_s_at | 1.558698819 | 4.924497116 | 3.88009642 | 0.000838998 | 0.010331221 | -0.802040511 | TDP1 |
| 202991_at | 1.564326281 | 4.814127725 | 4.53122605 | 0.000174417 | 0.003455567 | 0.719211054 | STARD3 |
| 212457_at | 1.573113923 | 7.578825278 | 6.272305149 | 2.91803E-06 | 0.000208745 | 4.711977555 | TFE3 |
| 219278_at | 1.57349624 | 5.491274899 | 3.533781751 | 0.001922594 | 0.01861831 | -1.598173155 | MAP3K6 |
| 202464_s_at | 1.57707995 | 9.751030674 | 3.267505046 | 0.003608355 | 0.02873129 | -2.197861652 | PFKFB3 |
| 209747_at | 1.580021448 | 7.866045883 | 5.224274821 | 3.31921E-05 | 0.001080008 | 2.33651756 | TGFB3 |
| 225108_at | 1.587424127 | 6.749348274 | 5.931611018 | 6.34944E-06 | 0.00034983 | 3.952532548 | AGPS |
| 219361_s_at | 1.587779282 | 5.298655702 | 4.411590891 | 0.000232764 | 0.004177502 | 0.438779647 | AEN |
| 218681_s_at | 1.596121723 | 5.377323467 | 3.934273892 | 0.000736423 | 0.009459724 | -0.676315907 | SDF2L1 |
| 241899_at | 1.596646293 | 3.840409527 | 3.185793146 | 0.004368778 | 0.03282505 | -2.379003222 | LOC553103 |
| 211724_x_at | 1.599156741 | 9.435641368 | 5.806210929 | 8.48134E-06 | 0.000434106 | 3.669636194 | MIOS |
| 218151_x_at | 1.599854584 | 5.691266743 | 5.207902024 | 3.45065E-05 | 0.00110402 | 2.298596516 | SLC52A2 |
| 225933_at | 1.601013928 | 5.214519271 | 4.593857208 | 0.000149984 | 0.003092229 | 0.866005522 | CCDC137 |
| 219522_at | 1.60355967 | 3.513533848 | 3.181667341 | 0.004411041 | 0.033082342 | -2.388108644 | FJX1 |
| 222681_at | 1.609196401 | 5.299193536 | 4.765509062 | 9.9251E-05 | 0.002360955 | 1.267987133 | POGLUT1 |
| 228937_at | 1.610430106 | 4.962981002 | 4.07559878 | 0.000523808 | 0.007437282 | -0.347302402 | LACC1 |
| 200919_at | 1.612294088 | 9.686644001 | 5.745398213 | 9.76573E-06 | 0.000473371 | 3.531835506 | PHC2 |
| 219298_at | 1.616820816 | 9.478195374 | 4.628921181 | 0.00013784 | 0.002905606 | 0.948168486 | ECHDC3 |
| 205192_at | 1.617341364 | 6.729081922 | 5.628943846 | 1.2807E-05 | 0.00055757 | 3.266889497 | MAP3K14 |
| 219189_at | 1.628206111 | 4.38845064 | 2.992475539 | 0.006836787 | 0.04515776 | -2.801015007 | FBXL6 |
| 200924_s_at | 1.633248375 | 6.891650132 | 7.393356709 | 2.5044E-07 | 4.15239E-05 | 7.103906603 | SLC3A2 |
| 232735_at | 1.638216254 | 4.247998751 | 2.957657802 | 0.007405385 | 0.04759931 | -2.875940684 | ANKRD34A |
| 212186_at | 1.641276996 | 7.204502115 | 4.814430081 | 8.82548E-05 | 0.002158136 | 1.382406925 | ACACA |
| 238853_at | 1.648065055 | 6.494108424 | 5.454034406 | 1.92933E-05 | 0.00074166 | 2.866489215 | RAB3IP |
| 208890_s_at | 1.649097572 | 6.217206993 | 4.62701767 | 0.000138473 | 0.002915899 | 0.943708603 | PLXNB2 |
| 210809_s_at | 1.663163653 | 6.258870759 | 3.175533189 | 0.00447461 | 0.033431903 | -2.40163889 | POSTN |
| 201482_at | 1.663361039 | 6.656092885 | 6.570622055 | 1.49434E-06 | 0.000135606 | 5.365132013 | QSOX1 |
| 225713_at | 1.663444536 | 4.029212783 | 3.045757663 | 0.006047138 | 0.041373803 | -2.685687621 | STK11IP |
| 212850_s_at | 1.663520188 | 6.188904983 | 4.153990249 | 0.000433521 | 0.006576431 | -0.16427383 | LRP4 |
| 205421_at | 1.666076706 | 7.635423393 | 2.832672867 | 0.009843474 | 0.05772601 | -3.141870772 | SLC22A3 |
| 205224_at | 1.6669585 | 4.002380357 | 3.304631021 | 0.00330696 | 0.02713007 | -2.115073111 | SURF2 |
| 209190_s_at | 1.673092545 | 8.612054699 | 7.986333711 | 7.30916E-08 | 1.85245E-05 | 8.297091274 | DIAPH1 |
| 220612_at | 1.677764583 | 5.834177511 | 5.439294655 | 1.99738E-05 | 0.000759707 | 2.832620273 | AF090939 |
| 231559_at | 1.69118376 | 4.396540422 | 4.111344242 | 0.000480522 | 0.007036684 | -0.263883423 | NNMT |
| 230047_at | 1.701388938 | 3.725161177 | 3.782273087 | 0.001061346 | 0.012154749 | -1.028357271 | ARHGAP42 |
| 214563_at | 1.703456463 | 3.054381726 | 3.374678287 | 0.002803686 | 0.024222156 | -1.958100114 | PCDHGC3 |
| 235938_at | 1.708371807 | 3.23194703 | 3.942979606 | 0.000721143 | 0.009328328 | -0.656090443 | ARMC9 |
| 238214_at | 1.711114747 | 3.055429879 | 3.369322258 | 0.002839369 | 0.024446906 | -1.970137029 | LRRC69 |
| 227506_at | 1.712765952 | 4.810001702 | 3.478561833 | 0.002192271 | 0.020432408 | -1.723588152 | SLC16A9 |
| 204039_at | 1.714310531 | 7.235625149 | 4.470040478 | 0.000202147 | 0.003787006 | 0.575784816 | CEBPA |
| 241290_at | 1.73242441 | 1.601177984 | 3.213845066 | 0.004091666 | 0.031363249 | -2.316988258 | LOC101927342 |
| 213479_at | 1.735737273 | 5.377369099 | 3.511045583 | 0.002029442 | 0.019372786 | -1.649871497 | NPTX2 |
| 225252_at | 1.739218207 | 8.005409018 | 5.453948005 | 1.92972E-05 | 0.00074166 | 2.866290738 | SRXN1 |
| 228868_x_at | 1.74706502 | 2.71441085 | 3.265890999 | 0.003622047 | 0.028789754 | -2.201454177 | CDT1 |
| 224523_s_at | 1.759690066 | 8.533976888 | 6.435368542 | 2.02128E-06 | 0.000165251 | 5.070423123 | CMSS1 |
| 225874_at | 1.759972036 | 5.664697883 | 5.979977836 | 5.68126E-06 | 0.000321802 | 4.061178066 | UBALD1 |
| 242888_at | 1.76612373 | 6.713739565 | 4.153242338 | 0.000434305 | 0.006585468 | -0.1660215 | PRRT3-AS1 |
| 205866_at | 1.778354277 | 5.309557439 | 3.365624263 | 0.002864264 | 0.024577952 | -1.978444513 | FCN3 |
| 203580_s_at | 1.780153224 | 9.101086829 | 7.659925993 | 1.43132E-07 | 3.0051E-05 | 7.646625098 | SLC7A6 |
| 1568777_at | 1.799303241 | 2.438760623 | 3.712599278 | 0.001254351 | 0.013745176 | -1.188927573 | EML5 |
| 235520_at | 1.807847909 | 4.068385716 | 3.58553184 | 0.001699575 | 0.017044745 | -1.480202404 | ZNF280C |
| 1564200_at | 1.809326698 | 2.852118292 | 3.637589447 | 0.001500946 | 0.015613184 | -1.361134362 | LINC00607 |
| 1558128_at | 1.811198672 | 3.474022475 | 3.093381689 | 0.005416339 | 0.038329186 | -2.581952147 | LOC730202 |
| 205567_at | 1.81129265 | 5.977898359 | 3.800736465 | 0.001015326 | 0.011780835 | -0.985715882 | CHST1 |
| 224520_s_at | 1.817646048 | 8.605091496 | 5.868057549 | 7.35125E-06 | 0.000388729 | 3.809374581 | BEST3 |
| 222925_at | 1.822842867 | 2.867166306 | 3.696518894 | 0.001303602 | 0.014107635 | -1.225905072 | DCDC2 |
| 235825_at | 1.82413683 | 4.031886282 | 3.166948433 | 0.004565067 | 0.03396717 | -2.420559368 | LOC101927330 |
| 200820_at | 1.825417803 | 10.70693387 | 9.340058791 | 5.22983E-09 | 3.44115E-06 | 10.8300009 | PSMD8 |
| 204254_s_at | 1.834994083 | 5.406147928 | 4.460027966 | 0.000207089 | 0.003851121 | 0.552314355 | VDR |
| 228037_at | 1.838855065 | 4.898714762 | 5.091340063 | 4.55232E-05 | 0.001343337 | 2.028100346 | RARA-AS1 |
| 244422_at | 1.844317051 | 2.468943017 | 3.242062135 | 0.003830166 | 0.029888268 | -2.254425742 | LOC101928370 |
| 1552727_s_at | 1.844854719 | 5.692106055 | 3.640861219 | 0.001489256 | 0.015541701 | -1.353638429 | ADAMTS17 |
| 221778_at | 1.850391303 | 8.964374227 | 9.71689929 | 2.61615E-09 | 2.26406E-06 | 11.488952 | KDM7A |
| 232000_at | 1.860993464 | 4.639768324 | 4.540689578 | 0.000170483 | 0.003398366 | 0.741393638 | TTC39B |
| 218590_at | 1.872561681 | 6.473671028 | 6.359979216 | 2.39428E-06 | 0.00018278 | 4.905122995 | C10orf2 |
| 210519_s_at | 1.873453297 | 7.0943203 | 3.948358547 | 0.00071186 | 0.009241944 | -0.643590846 | NQO1 |
| 237489_at | 1.88461357 | 1.883920863 | 3.473837619 | 0.002216995 | 0.020565601 | -1.734294305 | NLRP2 |
| 203612_at | 1.888824919 | 7.160310353 | 7.76632088 | 1.14794E-07 | 2.57915E-05 | 7.860355942 | BYSL |
| 213050_at | 1.889675205 | 5.216974047 | 5.275715663 | 2.93838E-05 | 0.000992004 | 2.455532937 | COBL |
| 213943_at | 1.890789914 | 4.533590528 | 3.651929171 | 0.001450371 | 0.015261588 | -1.32827002 | TWIST1 |
| 206766_at | 1.899215259 | 5.661825789 | 5.132209606 | 4.13038E-05 | 0.001253671 | 2.123044582 | ITGA10 |
| 231789_at | 1.909393056 | 2.318812188 | 3.438802983 | 0.002409068 | 0.021815265 | -1.813570776 | PCDHB15 |
| 207024_at | 1.911282912 | 5.952850848 | 5.714936693 | 1.0482E-05 | 0.000495699 | 3.462665095 | CHRND |
| 208928_at | 1.921929864 | 6.626094341 | 6.323098816 | 2.60175E-06 | 0.000190835 | 4.823993874 | POR |
| 200862_at | 1.923625435 | 8.801616182 | 4.931756895 | 6.66294E-05 | 0.001778891 | 1.656443362 | DHCR24 |
| 227940_at | 1.934926336 | 1.391390803 | 3.184110372 | 0.004385968 | 0.03290821 | -2.382717499 | LOC339803 |
| 214482_at | 1.93698705 | 3.077445569 | 3.215213583 | 0.004078592 | 0.031301923 | -2.313958179 | ZBTB25 |
| 225436_at | 1.941033959 | 3.502112014 | 4.473273128 | 0.000200577 | 0.003767647 | 0.583362566 | ABHD17C |
| 242582_at | 1.952707689 | 3.5839566 | 3.535460852 | 0.001914926 | 0.018579549 | -1.594351895 | RP11-330O11.3 |
| 226333_at | 1.957717074 | 8.559450682 | 6.429849963 | 2.04644E-06 | 0.000166924 | 5.058347792 | IL6R |
| 201328_at | 1.959439347 | 8.910022538 | 6.729934498 | 1.05011E-06 | 0.000108 | 5.709146512 | ETS2 |
| 222872_x_at | 1.967164285 | 5.060724881 | 5.0215122 | 5.37685E-05 | 0.001518961 | 1.865648691 | NABP1 |
| 208081_s_at | 1.986176796 | 3.10911778 | 4.061357288 | 0.000542117 | 0.007599925 | -0.380517482 | ZNF442 |
| 215411_s_at | 1.99375859 | 5.928740685 | 4.848975983 | 8.12381E-05 | 0.002056213 | 1.463153614 | TRAF3IP2 |
| 202613_at | 1.997944304 | 6.788037991 | 12.588788 | 2.25733E-11 | 9.33559E-08 | 15.91429301 | CTPS1 |
| 214123_s_at | 2.002966642 | 2.846237188 | 3.957608956 | 0.000696173 | 0.009098202 | -0.622089406 | NOP14-AS1 |
| 230788_at | 2.016049404 | 4.807974578 | 3.313059492 | 0.003242031 | 0.02677842 | -2.096237758 | GCNT2 |
| 209841_s_at | 2.028981244 | 6.073498777 | 3.021609716 | 0.006393479 | 0.043046189 | -2.738053108 | LRRN3 |
| 234426_x_at | 2.03600786 | 1.55895459 | 3.548308897 | 0.001857238 | 0.018139208 | -1.56509803 | PIH2 |
| 218178_s_at | 2.037298348 | 9.923386422 | 5.514416656 | 1.67427E-05 | 0.000676349 | 3.005034579 | CHMP1B |
| 228188_at | 2.038158019 | 8.554974713 | 6.794882759 | 9.10286E-07 | 9.72855E-05 | 5.848407859 | FOSL2 |
| 228368_at | 2.04541236 | 4.170731111 | 3.487111103 | 0.002148215 | 0.020158157 | -1.704203963 | ARHGAP20 |
| 205074_at | 2.05946461 | 7.934410301 | 6.443418205 | 1.98514E-06 | 0.000164957 | 5.088029711 | SLC22A5 |
| 235551_at | 2.060459265 | 4.20814683 | 4.668438047 | 0.000125335 | 0.002734591 | 1.040740914 | AX747730 |
| 1569052_at | 2.078227457 | 3.71402069 | 5.048192078 | 5.04531E-05 | 0.00145027 | 1.927751867 | RP11-288H12.4 |
| 225282_at | 2.094421699 | 5.919502116 | 5.845036766 | 7.75276E-06 | 0.000406327 | 3.757409055 | SMAP2 |
| 226800_at | 2.113667392 | 7.623929808 | 4.486328817 | 0.000194359 | 0.003695332 | 0.613966952 | EFCAB7 |
| 219050_s_at | 2.123526713 | 5.001340093 | 6.933650603 | 6.72033E-07 | 8.11057E-05 | 6.144007508 | ZNHIT2 |
| 1560513_at | 2.136816012 | 3.95359486 | 4.135164821 | 0.000453675 | 0.006774963 | -0.208255928 | LOC400568 |
| 230748_at | 2.145031467 | 6.152240415 | 5.051626822 | 5.00416E-05 | 0.001441214 | 1.935744021 | SLC16A6 |
| 203709_at | 2.161085778 | 5.353266161 | 5.367739832 | 2.36412E-05 | 0.000855936 | 2.667938165 | PHKG2 |
| 224657_at | 2.161788296 | 8.571676577 | 5.900006145 | 6.82892E-06 | 0.000365917 | 3.881396751 | ERRFI1 |
| 230227_at | 2.162248526 | 2.18034311 | 3.72457479 | 0.00121887 | 0.013468687 | -1.161368955 | RP11-333I13.1 |
| 225615_at | 2.173100794 | 5.431148359 | 6.795559821 | 9.08934E-07 | 9.72855E-05 | 5.84985657 | IFFO2 |
| 204151_x_at | 2.184013152 | 10.48683827 | 4.997798573 | 5.69001E-05 | 0.001582002 | 1.810416877 | AKR1C1 |
| 1561136_at | 2.191788506 | 1.188756139 | 2.836139296 | 0.009766554 | 0.057421185 | -3.134562159 | GYPE |
| 208078_s_at | 2.230759812 | 6.101091671 | 4.331332256 | 0.000282523 | 0.004849769 | 0.250713704 | SIK1 |
| 202934_at | 2.231566334 | 8.399376862 | 3.980914952 | 0.000658156 | 0.008736515 | -0.567888502 | HK2 |
| 227863_at | 2.238302448 | 4.461167396 | 4.400055834 | 0.000239335 | 0.004271666 | 0.411744708 | IFITM10 |
| 216952_s_at | 2.254516146 | 4.473250644 | 6.638763873 | 1.28452E-06 | 0.000123011 | 5.512692732 | LMNB2 |
| 203744_at | 2.257352366 | 7.052913349 | 5.937391764 | 6.26553E-06 | 0.000346823 | 3.965531664 | HMGB3 |
| 204736_s_at | 2.266255083 | 4.831838106 | 5.467971921 | 1.86715E-05 | 0.000726428 | 2.898497286 | CSPG4 |
| 58308_at | 2.289383855 | 5.191564976 | 7.160012139 | 4.11887E-07 | 5.91185E-05 | 6.620421048 | TRIM62 |
| 232510_s_at | 2.315309386 | 6.714825146 | 7.10737267 | 4.61272E-07 | 6.37851E-05 | 6.510278375 | DPP3 |
| 228813_at | 2.318993986 | 9.775396961 | 9.722146553 | 2.59136E-09 | 2.26406E-06 | 11.49798993 | HDAC4 |
| 201037_at | 2.322392883 | 7.954107737 | 5.839353666 | 7.85528E-06 | 0.000407752 | 3.744571537 | PFKP |
| 204563_at | 2.324147004 | 3.031573174 | 4.054676701 | 0.000550924 | 0.007687195 | -0.396094274 | SELL |
| 203867_s_at | 2.345065648 | 4.646094555 | 5.463882054 | 1.88518E-05 | 0.000729444 | 2.889106511 | NLE1 |
| 204087_s_at | 2.346222186 | 5.63360247 | 6.758959016 | 9.85082E-07 | 0.000103105 | 5.771451775 | SLC5A6 |
| 1552986_at | 2.35651335 | 2.525377738 | 4.716596652 | 0.000111628 | 0.002537339 | 1.153512558 | LOC142937 |
| 216548_x_at | 2.364185417 | 4.288257367 | 4.984908971 | 5.86791E-05 | 0.001619013 | 1.7803828 | HMGB3P1 |
| 219181_at | 2.383050034 | 3.718025385 | 4.584861089 | 0.000153269 | 0.003141715 | 0.844922851 | LIPG |
| 208581_x_at | 2.391606711 | 12.02687878 | 10.05578206 | 1.42405E-09 | 1.57901E-06 | 12.06501212 | MT1X |
| 221601_s_at | 2.433591491 | 4.849713741 | 3.495063653 | 0.002108014 | 0.019882122 | -1.686161766 | FAIM3 |
| 208383_s_at | 2.44767185 | 3.757340932 | 4.463320281 | 0.000205451 | 0.003824665 | 0.56003188 | PCK1 |
| 212859_x_at | 2.503061634 | 10.72602454 | 7.490766187 | 2.0391E-07 | 3.58175E-05 | 7.303419999 | MT1E |
| 212218_s_at | 2.516440519 | 5.849645013 | 4.488634808 | 0.000193281 | 0.003681383 | 0.619372535 | FASN |
| 209824_s_at | 2.51906831 | 6.186255054 | 5.315905968 | 2.67192E-05 | 0.000931289 | 2.548379999 | ARNTL |
| 230102_at | 2.553477602 | 6.5099729 | 6.519293677 | 1.67538E-06 | 0.000147066 | 5.253573409 | ETV5 |
| 1563426_a_at | 2.564494398 | 2.623096245 | 4.516436813 | 0.000180748 | 0.003541306 | 0.684544067 | AP001462.6 |
| 239380_at | 2.584465287 | 5.536284811 | 6.590381583 | 1.43011E-06 | 0.0001318 | 5.407984909 | C5orf27 |
| 204745_x_at | 2.59514077 | 9.234561342 | 10.73996645 | 4.34486E-10 | 6.43456E-07 | 13.1819239 | MT1G |
| 200832_s_at | 2.597875978 | 5.818714958 | 3.258039524 | 0.003689376 | 0.02913564 | -2.218921797 | SCD |
| 227425_at | 2.642746452 | 4.302816072 | 3.509028758 | 0.002039196 | 0.019424096 | -1.654453416 | REPS2 |
| 222139_at | 2.648156222 | 6.595617495 | 13.4037101 | 6.80308E-12 | 4.82774E-08 | 16.99853696 | ERV3-2 |
| 204802_at | 2.664163951 | 10.7429651 | 9.066486036 | 8.74337E-09 | 4.92432E-06 | 10.33925506 | RRAD |
| 202431_s_at | 2.688613444 | 7.627288994 | 5.416300811 | 2.10846E-05 | 0.000784218 | 2.779747599 | MYC |
| 36711_at | 2.724412665 | 6.870461888 | 6.06066231 | 4.72222E-06 | 0.00028303 | 4.241820372 | MAFF |
| 228903_at | 2.728223225 | 2.881090317 | 3.753811157 | 0.00113636 | 0.012812309 | -1.094017732 | CES4A |
| 212185_x_at | 2.745920424 | 12.16206279 | 10.87698118 | 3.44735E-10 | 6.43456E-07 | 13.39840006 | MT2A |
| 203665_at | 2.77490224 | 7.299285971 | 4.751973418 | 0.000102531 | 0.002408545 | 1.236315302 | HMOX1 |
| 1557527_at | 2.777685479 | 5.267053731 | 5.525191842 | 1.6325E-05 | 0.000664787 | 3.029723336 | AX746823 |
| 205937_at | 2.784184366 | 3.903141696 | 6.699883561 | 1.12209E-06 | 0.000111402 | 5.644517168 | CGREF1 |
| 233030_at | 2.7863651 | 4.600635386 | 3.551385253 | 0.00184368 | 0.01807112 | -1.558089642 | PNPLA3 |
| 211456_x_at | 2.819888019 | 11.52134599 | 9.451048911 | 4.25694E-09 | 3.14676E-06 | 11.02611636 | MT1HL1 |
| 203725_at | 2.844922683 | 9.298460336 | 7.313324212 | 2.96795E-07 | 4.57865E-05 | 6.938960513 | GADD45A |
| 203649_s_at | 2.854956388 | 6.653504743 | 5.782896693 | 8.95205E-06 | 0.000448006 | 3.616852591 | PLA2G2A |
| 202284_s_at | 2.864782843 | 8.434782674 | 4.824819911 | 8.60824E-05 | 0.002124265 | 1.40669658 | CDKN1A |
| 228864_at | 2.867120117 | 3.155759582 | 6.508164737 | 1.71751E-06 | 0.000148636 | 5.229339624 | ZNF653 |
| 236717_at | 2.867300604 | 7.332987203 | 5.013966095 | 5.47457E-05 | 0.001540433 | 1.848076261 | FAM179A |
| 232812_at | 2.908756672 | 2.915330015 | 5.512783626 | 1.6807E-05 | 0.000677665 | 3.001291956 | LOC401052 |
| 219584_at | 2.991660883 | 6.525014895 | 5.490489257 | 1.77097E-05 | 0.000699749 | 2.950172897 | PLA1A |
| 205258_at | 3.10143684 | 6.451379493 | 6.619445555 | 1.34074E-06 | 0.000126821 | 5.47092202 | INHBB |
| 217165_x_at | 3.101996459 | 9.895084005 | 10.7202107 | 4.49303E-10 | 6.43456E-07 | 13.15051626 | MT1F |
| 206633_at | 3.112597625 | 9.336944768 | 6.907728397 | 7.11092E-07 | 8.41032E-05 | 6.088991368 | CHRNA1 |
| 209160_at | 3.142001849 | 9.58850633 | 5.53669602 | 1.58907E-05 | 0.000651079 | 3.056070623 | AKR1C3 |
| 220918_at | 3.276141443 | 5.195020408 | 6.58569478 | 1.44508E-06 | 0.000132151 | 5.39782524 | RUNX1-IT1 |
| 206461_x_at | 3.391063619 | 11.12281999 | 11.03358289 | 2.65291E-10 | 6.27537E-07 | 13.64295834 | MT1H |
| 206662_at | 3.589305931 | 11.13984409 | 13.9179479 | 3.2871E-12 | 2.91582E-08 | 17.64842911 | GLRX |
| 219270_at | 3.771167965 | 5.772756498 | 4.234462793 | 0.000356971 | 0.005752059 | 0.023905903 | CHAC1 |
| 202917_s_at | 3.925390037 | 7.46901712 | 5.777386643 | 9.06713E-06 | 0.00045249 | 3.604369381 | S100A8 |
| 202376_at | 4.010464553 | 5.839735599 | 4.910803991 | 7.00553E-05 | 0.001845361 | 1.607547954 | SERPINA3 |
| 224579_at | 4.0370941 | 8.238140553 | 7.479988345 | 2.08588E-07 | 3.6103E-05 | 7.281412399 | SLC38A1 |
| 203535_at | 4.119349129 | 5.88351537 | 5.935581758 | 6.29168E-06 | 0.000347728 | 3.961461922 | S100A9 |
| 219995_s_at | 4.415892786 | 4.269559207 | 3.912269906 | 0.00077649 | 0.009832763 | -0.727408778 | ZNF750 |
| 209395_at | 5.295106515 | 5.317882916 | 9.391519977 | 4.7529E-09 | 3.31829E-06 | 10.9211437 | CHI3L1 |
| 203021_at | 5.702364742 | 9.401906287 | 20.15551399 | 2.03245E-15 | 7.21154E-11 | 23.84138625 | SLPI |
| 1564758_at | 6.190346649 | 5.56520708 | 8.734508761 | 1.65223E-08 | 7.04484E-06 | 9.729512499 | LOC643659 |
