# Supplementary material for: Identification and validation of oxidative stress-related genes in sepsis-induced myopathy
Source: Medicine (Baltimore). 2024 May 3;103(18):e37933. doi: 10.1097/MD.0000000000037933 (PMC11062695; doi:10.1097/MD.0000000000037933)
Supplement: Supplementary file 3 [file medi-103-e37933-s005.docx]

| Supplementary Table 3. 319 miRNAs of 5 diagnostic genes | | | | | |
| --- | --- | --- | --- | --- | --- |
| ID | Accession | Target | TargetID | Experiment | Literature |
| hsa-let-7b-5p | MIMAT0000063 | GSR | 2936 | CLASH//Proteomics | 18668040\|23622248 |
| hsa-mir-15a-5p | MIMAT0000068 | TP53 | 7157 | Immunoblot//Luciferase reporter assay | 21205967 |
| hsa-mir-16-5p | MIMAT0000069 | TP53 | 7157 | Immunoblot//Luciferase reporter assay | 21205967 |
| hsa-mir-17-5p | MIMAT0000070 | TP53 | 7157 | qRT-PCR | 24955218 |
| hsa-mir-17-3p | MIMAT0000071 | GSR | 2936 | HITS-CLIP | 23706177 |
| hsa-mir-18a-5p | MIMAT0000072 | TP53 | 7157 | qRT-PCR | 24955218 |
| hsa-mir-19a-3p | MIMAT0000073 | TP53 | 7157 | HITS-CLIP | 22473208 |
| hsa-mir-19b-3p | MIMAT0000074 | TP53 | 7157 | HITS-CLIP//Immunoblot//Immunohistochemistry//Luciferase reporter assay//qRT-PCR | 22473208\|24742936 |
| hsa-mir-20a-5p | MIMAT0000075 | TP53 | 7157 | qRT-PCR | 24955218 |
| hsa-mir-24-3p | MIMAT0000080 | TP53 | 7157 | Immunohistochemistry//Luciferase reporter assay//qRT-PCR//Western blot | 27780140 |
| hsa-mir-25-3p | MIMAT0000081 | TP53 | 7157 | Luciferase reporter assay//qRT-PCR//Western blot | 20935678 |
| hsa-mir-26b-5p | MIMAT0000083 | CD36 | 948 | Microarray | 19088304 |
| hsa-mir-26b-5p | MIMAT0000083 | GSR | 2936 | Sequencing | 20371350 |
| hsa-mir-27a-3p | MIMAT0000084 | TP53 | 7157 | Luciferase reporter assay//qRT-PCR | 20542001\|26687066 |
| hsa-mir-28-5p | MIMAT0000085 | TP53 | 7157 | Luciferase reporter assay | 24681953 |
| hsa-mir-30a-5p | MIMAT0000087 | TP53 | 7157 | Luciferase reporter assay//qRT-PCR//Western blot | 24029422 |
| hsa-mir-92a-3p | MIMAT0000092 | GPX3 | 2878 | CLASH | 23622248 |
| hsa-mir-106a-5p | MIMAT0000103 | TP53 | 7157 | qRT-PCR | 24955218 |
| hsa-mir-30c-5p | MIMAT0000244 | TP53 | 7157 | Flow//GFP reporter assay//Immunoblot//Luciferase reporter assay//Next Generation Sequencing (NGS)//Northern blot//qRT-PCR//Western blot | 24029422\|28330939 |
| hsa-mir-30d-5p | MIMAT0000245 | TP53 | 7157 | Luciferase reporter assay//qRT-PCR//Western blot | 20935678\|24029422 |
| hsa-mir-10a-5p | MIMAT0000253 | GSR | 2936 | HITS-CLIP | 23824327 |
| hsa-mir-10b-5p | MIMAT0000254 | GSR | 2936 | HITS-CLIP | 23824327 |
| hsa-mir-10b-5p | MIMAT0000254 | TP53 | 7157 | Luciferase reporter assay | 23034333 |
| hsa-mir-34a-5p | MIMAT0000255 | TP53 | 7157 | Flow//Immunoblot//Immunocytochemistry//Immunohistochemistry//Luciferase reporter assay//Microarray//Next Generation Sequencing (NGS)//Northern blot//qRT-PCR//QRTPCR//Western blot | 23292869\|26406332\|26403328\|26177460 |
| hsa-mir-183-5p | MIMAT0000261 | GSR | 2936 | CLASH | 23622248 |
| hsa-mir-214-3p | MIMAT0000271 | GSR | 2936 | Luciferase reporter assay | 23905773 |
| hsa-mir-214-3p | MIMAT0000271 | TP53 | 7157 | Luciferase reporter assay//Reporter assay | 22927443\|21217645 |
| hsa-mir-221-3p | MIMAT0000278 | TP53 | 7157 | Western blot | 21226887 |
| hsa-mir-222-3p | MIMAT0000279 | TP53 | 7157 | CLASH//Western blot | 21226887\|23622248 |
| hsa-mir-223-3p | MIMAT0000280 | TP53 | 7157 | PAR-CLIP | 23592263 |
| hsa-mir-30b-5p | MIMAT0000420 | TP53 | 7157 | Luciferase reporter assay//qRT-PCR//Western blot | 24029422 |
| hsa-mir-125b-5p | MIMAT0000423 | TP53 | 7157 | In situ hybridization//Luciferase reporter assay//Next Generation Sequencing (NGS)//qRT-PCR//Reporter assay//Western blot | 20216554\|19293287\|21935352\|23497288\|22331826\|26585673 |
| hsa-mir-125a-5p | MIMAT0000443 | TP53 | 7157 | Luciferase reporter assay//Northern blot//qRT-PCR//Western blot | 19818772\|26017674 |
| hsa-mir-150-5p | MIMAT0000451 | TP53 | 7157 | Luciferase reporter assay//qRT-PCR//Western blot | 23747308\|23670238 |
| hsa-mir-185-5p | MIMAT0000455 | TP53 | 7157 | PAR-CLIP | 23592263 |
| hsa-mir-155-5p | MIMAT0000646 | CD36 | 948 | Proteomics | 20584899 |
| hsa-mir-106b-5p | MIMAT0000680 | TP53 | 7157 | qRT-PCR | 24955218 |
| hsa-mir-200a-3p | MIMAT0000682 | TP53 | 7157 | Luciferase reporter assay//Western blot | 23144891 |
| hsa-mir-30e-5p | MIMAT0000692 | TP53 | 7157 | Luciferase reporter assay//qRT-PCR//Western blot | 24029422 |
| hsa-mir-375 | MIMAT0000728 | TP53 | 7157 | Flow//Luciferase reporter assay//qRT-PCR//Western blot | 23835407 |
| hsa-mir-377-3p | MIMAT0000730 | GSR | 2936 | HITS-CLIP | 23824327\|19536157 |
| hsa-mir-377-3p | MIMAT0000730 | TP53 | 7157 | Luciferase reporter assay//qRT-PCR//Western blot | 25998046 |
| hsa-mir-330-3p | MIMAT0000751 | TP53 | 7157 | HITS-CLIP | 28735896 |
| hsa-mir-324-5p | MIMAT0000761 | TP53 | 7157 | CLASH | 23622248 |
| hsa-mir-335-5p | MIMAT0000765 | CD36 | 948 | Microarray | 18185580 |
| hsa-mir-448 | MIMAT0001532 | CD36 | 948 | HITS-CLIP | 27418678 |
| hsa-mir-431-5p | MIMAT0001625 | GSR | 2936 | HITS-CLIP | 23824327 |
| hsa-mir-485-5p | MIMAT0002175 | GSR | 2936 | HITS-CLIP | 23706177 |
| hsa-mir-485-5p | MIMAT0002175 | TP53 | 7157 | qRT-PCR | 20542001 |
| hsa-mir-491-5p | MIMAT0002807 | TP53 | 7157 | Flow cytometry//GFP reporter//PAR-CLIP//QRTPCR//Western blot | 23519249\|23592263 |
| hsa-mir-518c-3p | MIMAT0002848 | TP53 | 7157 | Luciferase reporter assay//qRT-PCR//Western blot | 25088422 |
| hsa-mir-504-5p | MIMAT0002875 | TP53 | 7157 | qRT-PCR//Reporter assay//Western blot | 20542001 |
| hsa-mir-455-5p | MIMAT0003150 | CD36 | 948 | HITS-CLIP | 23824327\|27418678 |
| hsa-mir-558 | MIMAT0003222 | GSR | 2936 | PAR-CLIP | 23592263 |
| hsa-mir-569 | MIMAT0003234 | GSR | 2936 | PAR-CLIP | 22100165 |
| hsa-mir-570-3p | MIMAT0003235 | GSR | 2936 | PAR-CLIP | 27292025 |
| hsa-mir-605-5p | MIMAT0003273 | TP53 | 7157 | qRT-PCR//Reporter assay | 21217645 |
| hsa-mir-608 | MIMAT0003276 | TP53 | 7157 | PAR-CLIP//qRT-PCR | 20542001\|23592263 |
| hsa-mir-612 | MIMAT0003280 | TP53 | 7157 | Luciferase reporter assay//qRT-PCR//Western blot | 20417621 |
| hsa-mir-548c-3p | MIMAT0003285 | GSR | 2936 | HITS-CLIP//PAR-CLIP | 23446348\|21572407 |
| hsa-mir-622 | MIMAT0003291 | TP53 | 7157 | PAR-CLIP | 23592263 |
| hsa-mir-638 | MIMAT0003308 | TP53 | 7157 | Luciferase reporter assay//qRT-PCR//Western blot | 25088422 |
| hsa-mir-640 | MIMAT0003310 | GSR | 2936 | HITS-CLIP | 23824327 |
| hsa-mir-650 | MIMAT0003320 | GSR | 2936 | PAR-CLIP | 23592263 |
| hsa-mir-661 | MIMAT0003324 | CD36 | 948 | PAR-CLIP | 23446348 |
| hsa-mir-663a | MIMAT0003326 | TP53 | 7157 | Luciferase reporter assay | 27105517 |
| hsa-mir-454-3p | MIMAT0003885 | TP53 | 7157 | CLASH | 23622248 |
| hsa-mir-23a-5p | MIMAT0004496 | GSR | 2936 | HITS-CLIP | 23824327\|19536157 |
| hsa-mir-26a-1-3p | MIMAT0004499 | TP53 | 7157 | qRT-PCR//Western blot | 27517917 |
| hsa-mir-28-3p | MIMAT0004502 | TP53 | 7157 | Luciferase reporter assay | 24681953 |
| hsa-mir-92a-1-5p | MIMAT0004507 | TP53 | 7157 | qRT-PCR | 24955218 |
| hsa-mir-92a-2-5p | MIMAT0004508 | TP53 | 7157 | qRT-PCR | 24955218 |
| hsa-mir-214-5p | MIMAT0004564 | TP53 | 7157 | qRT-PCR//Western blot | 25962755 |
| hsa-mir-23b-5p | MIMAT0004587 | GSR | 2936 | HITS-CLIP | 23824327\|19536157 |
| hsa-mir-125b-1-3p | MIMAT0004592 | TP53 | 7157 | Luciferase reporter assay | 27592685 |
| hsa-mir-149-3p | MIMAT0004609 | TP53 | 7157 | PAR-CLIP | 23592263 |
| hsa-mir-150-3p | MIMAT0004610 | TP53 | 7157 | qRT-PCR//Western blot | 27081855 |
| hsa-mir-155-3p | MIMAT0004658 | TP53 | 7157 | ChIP-seq//Luciferase reporter assay//Western blot | 27903673 |
| hsa-mir-194-3p | MIMAT0004671 | TP53 | 7157 | HITS-CLIP//PAR-CLIP | 23592263\|28735896 |
| hsa-mir-367-5p | MIMAT0004686 | GSR | 2936 | HITS-CLIP | 23824327 |
| hsa-mir-377-5p | MIMAT0004689 | GSR | 2936 | PAR-CLIP | 23592263 |
| hsa-mir-151a-5p | MIMAT0004697 | TP53 | 7157 | ChIP-seq//Luciferase reporter assay//qRT-PCR//Western blot | 27191259 |
| hsa-mir-505-5p | MIMAT0004776 | TP53 | 7157 | HITS-CLIP | 28735896 |
| hsa-mir-590-3p | MIMAT0004801 | GSR | 2936 | HITS-CLIP | 23313552 |
| hsa-mir-300 | MIMAT0004903 | TP53 | 7157 | Immunoprecipitaion//Luciferase reporter assay//qRT-PCR//Western blot | 26221215\|27779716\|26221232 |
| hsa-mir-744-5p | MIMAT0004945 | GSR | 2936 | CLASH | 23622248 |
| hsa-mir-744-3p | MIMAT0004946 | GSR | 2936 | HITS-CLIP | 23824327 |
| hsa-mir-665 | MIMAT0004952 | GSR | 2936 | PAR-CLIP | 23592263 |
| hsa-mir-1225-3p | MIMAT0005573 | TP53 | 7157 | HITS-CLIP | 28735896 |
| hsa-mir-1227-3p | MIMAT0005580 | GSR | 2936 | HITS-CLIP | 23706177 |
| hsa-mir-1228-3p | MIMAT0005583 | GSR | 2936 | HITS-CLIP | 23824327\|19536157 |
| hsa-mir-1228-3p | MIMAT0005583 | TP53 | 7157 | Immunohistochemistry//Luciferase reporter assay//qRT-PCR//Western blot | 25422913 |
| hsa-mir-1264 | MIMAT0005791 | TP53 | 7157 | HITS-CLIP | 19536157 |
| hsa-mir-1207-5p | MIMAT0005871 | TP53 | 7157 | PAR-CLIP | 23592263 |
| hsa-mir-1285-3p | MIMAT0005876 | TP53 | 7157 | Luciferase reporter assay//qRT-PCR//Western blot | 20417621 |
| hsa-mir-1254 | MIMAT0005905 | CD36 | 948 | PAR-CLIP | 23446348 |
| hsa-mir-1281 | MIMAT0005939 | GSR | 2936 | HITS-CLIP | 23824327\|19536157 |
| hsa-mir-1827 | MIMAT0006767 | GSR | 2936 | PAR-CLIP | 23592263 |
| hsa-mir-1909-3p | MIMAT0007883 | TP53 | 7157 | PAR-CLIP | 23592263 |
| hsa-mir-1972 | MIMAT0009447 | TP53 | 7157 | PAR-CLIP | 23592263 |
| hsa-mir-1976 | MIMAT0009451 | GSR | 2936 | HITS-CLIP | 23824327\|19536157 |
| hsa-mir-2053 | MIMAT0009978 | GSR | 2936 | PAR-CLIP | 22100165 |
| hsa-mir-2110 | MIMAT0010133 | TP53 | 7157 | PAR-CLIP | 23592263 |
| hsa-mir-2276-3p | MIMAT0011775 | GSR | 2936 | HITS-CLIP | 23824327\|19536157 |
| hsa-mir-3116 | MIMAT0014978 | CD36 | 948 | PAR-CLIP | 23446348 |
| hsa-mir-548s | MIMAT0014987 | GSR | 2936 | PAR-CLIP | 27292025 |
| hsa-mir-3149 | MIMAT0015022 | GSR | 2936 | HITS-CLIP | 23313552 |
| hsa-mir-3160-3p | MIMAT0015034 | GSR | 2936 | PAR-CLIP | 23592263 |
| hsa-mir-3165 | MIMAT0015039 | TP53 | 7157 | HITS-CLIP | 19536157 |
| hsa-mir-3065-5p | MIMAT0015066 | TP53 | 7157 | HITS-CLIP | 23824327 |
| hsa-mir-3187-3p | MIMAT0015069 | CD36 | 948 | PAR-CLIP | 23446348 |
| hsa-mir-3190-5p | MIMAT0015073 | CD36 | 948 | PAR-CLIP | 23446348 |
| hsa-mir-3190-5p | MIMAT0015073 | GSR | 2936 | PAR-CLIP | 27292025 |
| hsa-mir-548x-3p | MIMAT0015081 | GSR | 2936 | PAR-CLIP | 22100165 |
| hsa-mir-1273d | MIMAT0015090 | GSR | 2936 | PAR-CLIP | 27292025 |
| hsa-mir-4306 | MIMAT0016858 | TP53 | 7157 | PAR-CLIP | 23592263 |
| hsa-mir-4257 | MIMAT0016878 | GSR | 2936 | PAR-CLIP | 23592263 |
| hsa-mir-4252 | MIMAT0016886 | GSR | 2936 | HITS-CLIP | 23706177 |
| hsa-mir-4271 | MIMAT0016901 | TP53 | 7157 | PAR-CLIP | 23592263 |
| hsa-mir-4276 | MIMAT0016904 | TP53 | 7157 | HITS-CLIP | 28735896 |
| hsa-mir-4279 | MIMAT0016909 | GSR | 2936 | HITS-CLIP | 23824327\|19536157 |
| hsa-mir-4286 | MIMAT0016916 | TP53 | 7157 | HITS-CLIP | 23824327 |
| hsa-mir-3612 | MIMAT0017989 | GSR | 2936 | PAR-CLIP | 23592263 |
| hsa-mir-3620-3p | MIMAT0018001 | GSR | 2936 | HITS-CLIP | 23824327 |
| hsa-mir-1273e | MIMAT0018079 | GSR | 2936 | PAR-CLIP | 23592263 |
| hsa-mir-3926 | MIMAT0018201 | GSR | 2936 | PAR-CLIP | 27292025 |
| hsa-mir-4419a | MIMAT0018931 | TP53 | 7157 | PAR-CLIP | 23592263 |
| hsa-mir-4421 | MIMAT0018934 | GSR | 2936 | HITS-CLIP | 23824327 |
| hsa-mir-4434 | MIMAT0018950 | TP53 | 7157 | PAR-CLIP | 23592263 |
| hsa-mir-548ae-3p | MIMAT0018954 | GSR | 2936 | PAR-CLIP | 22100165 |
| hsa-mir-4438 | MIMAT0018956 | CD36 | 948 | PAR-CLIP | 23446348 |
| hsa-mir-4438 | MIMAT0018956 | GSR | 2936 | HITS-CLIP | 23313552 |
| hsa-mir-4459 | MIMAT0018981 | GSR | 2936 | PAR-CLIP | 23592263 |
| hsa-mir-548aj-3p | MIMAT0018990 | GSR | 2936 | PAR-CLIP | 22100165 |
| hsa-mir-4487 | MIMAT0019021 | GSR | 2936 | PAR-CLIP | 23592263 |
| hsa-mir-4495 | MIMAT0019030 | CD36 | 948 | HITS-CLIP | 23824327 |
| hsa-mir-4510 | MIMAT0019047 | TP53 | 7157 | PAR-CLIP | 23592263 |
| hsa-mir-4516 | MIMAT0019053 | TP53 | 7157 | PAR-CLIP | 23592263 |
| hsa-mir-4531 | MIMAT0019070 | TP53 | 7157 | PAR-CLIP | 23592263 |
| hsa-mir-548am-3p | MIMAT0019076 | GSR | 2936 | PAR-CLIP | 22100165 |
| hsa-mir-4537 | MIMAT0019080 | GSR | 2936 | HITS-CLIP | 23706177 |
| hsa-mir-4423-5p | MIMAT0019232 | GSR | 2936 | HITS-CLIP | 23824327 |
| hsa-mir-4529-5p | MIMAT0019236 | CD36 | 948 | PAR-CLIP | 23446348 |
| hsa-mir-4644 | MIMAT0019704 | TP53 | 7157 | PAR-CLIP | 23592263 |
| hsa-mir-4650-5p | MIMAT0019713 | TP53 | 7157 | PAR-CLIP | 23592263 |
| hsa-mir-4651 | MIMAT0019715 | TP53 | 7157 | PAR-CLIP | 23592263 |
| hsa-mir-4682 | MIMAT0019767 | TP53 | 7157 | PAR-CLIP | 23592263 |
| hsa-mir-1343-3p | MIMAT0019776 | GSR | 2936 | HITS-CLIP | 23824327\|19536157 |
| hsa-mir-4695-5p | MIMAT0019788 | GSR | 2936 | PAR-CLIP | 23592263 |
| hsa-mir-4697-3p | MIMAT0019792 | TP53 | 7157 | PAR-CLIP | 23592263 |
| hsa-mir-4710 | MIMAT0019815 | GSR | 2936 | PAR-CLIP | 23592263 |
| hsa-mir-4722-3p | MIMAT0019837 | GSR | 2936 | HITS-CLIP | 23824327\|19536157 |
| hsa-mir-4725-3p | MIMAT0019844 | TP53 | 7157 | PAR-CLIP | 23592263 |
| hsa-mir-4728-5p | MIMAT0019849 | TP53 | 7157 | PAR-CLIP | 23592263 |
| hsa-mir-4732-3p | MIMAT0019856 | GSR | 2936 | HITS-CLIP | 23824327 |
| hsa-mir-4735-5p | MIMAT0019860 | GSR | 2936 | HITS-CLIP | 23313552 |
| hsa-mir-4736 | MIMAT0019862 | TP53 | 7157 | PAR-CLIP | 23592263 |
| hsa-mir-4763-3p | MIMAT0019913 | TP53 | 7157 | PAR-CLIP | 23592263 |
| hsa-mir-4775 | MIMAT0019931 | GSR | 2936 | HITS-CLIP | 23313552 |
| hsa-mir-2467-3p | MIMAT0019953 | GSR | 2936 | HITS-CLIP//PAR-CLIP | 23592263\|23706177 |
| hsa-mir-4792 | MIMAT0019964 | GSR | 2936 | PAR-CLIP | 23592263 |
| hsa-mir-4795-5p | MIMAT0019968 | TP53 | 7157 | PAR-CLIP | 23592263 |
| hsa-mir-5095 | MIMAT0020600 | CD36 | 948 | PAR-CLIP | 23446348 |
| hsa-mir-5095 | MIMAT0020600 | GSR | 2936 | HITS-CLIP | 23313552 |
| hsa-mir-1273f | MIMAT0020601 | TP53 | 7157 | PAR-CLIP | 23592263 |
| hsa-mir-5096 | MIMAT0020603 | GSR | 2936 | HITS-CLIP | 23824327 |
| hsa-mir-548ah-3p | MIMAT0020957 | GSR | 2936 | PAR-CLIP | 22100165 |
| hsa-mir-5003-5p | MIMAT0021025 | TP53 | 7157 | HITS-CLIP | 23824327 |
| hsa-mir-5011-5p | MIMAT0021045 | GSR | 2936 | HITS-CLIP//PAR-CLIP | 23446348\|21572407 |
| hsa-mir-5193 | MIMAT0021124 | TP53 | 7157 | HITS-CLIP | 23824327 |
| hsa-mir-5196-3p | MIMAT0021129 | TP53 | 7157 | HITS-CLIP | 23824327 |
| hsa-mir-5197-5p | MIMAT0021130 | TP53 | 7157 | PAR-CLIP | 23592263 |
| hsa-mir-548aq-3p | MIMAT0022264 | GSR | 2936 | PAR-CLIP | 22100165 |
| hsa-mir-548as-3p | MIMAT0022268 | GSR | 2936 | PAR-CLIP | 27292025 |
| hsa-mir-5582-3p | MIMAT0022280 | GSR | 2936 | PAR-CLIP | 22100165 |
| hsa-mir-5587-3p | MIMAT0022290 | TP53 | 7157 | HITS-CLIP | 28735896 |
| hsa-mir-5692c | MIMAT0022476 | GSR | 2936 | HITS-CLIP//PAR-CLIP | 23446348\|21572407 |
| hsa-mir-5692a | MIMAT0022484 | GSR | 2936 | HITS-CLIP | 21572407 |
| hsa-mir-5693 | MIMAT0022486 | TP53 | 7157 | HITS-CLIP//PAR-CLIP | 23592263\|28735896 |
| hsa-mir-5702 | MIMAT0022495 | TP53 | 7157 | PAR-CLIP | 23592263 |
| hsa-mir-5703 | MIMAT0022496 | TP53 | 7157 | PAR-CLIP | 23592263 |
| hsa-mir-5692b | MIMAT0022497 | GSR | 2936 | HITS-CLIP//PAR-CLIP | 23446348\|21572407 |
| hsa-mir-660-3p | MIMAT0022711 | GSR | 2936 | HITS-CLIP | 23824327\|19536157 |
| hsa-mir-660-3p | MIMAT0022711 | TP53 | 7157 | HITS-CLIP | 23824327 |
| hsa-mir-1304-3p | MIMAT0022720 | GSR | 2936 | HITS-CLIP | 19536157 |
| hsa-mir-1247-3p | MIMAT0022721 | TP53 | 7157 | HITS-CLIP | 28735896 |
| hsa-mir-1277-5p | MIMAT0022724 | GSR | 2936 | HITS-CLIP//PAR-CLIP | 23446348\|21572407 |
| hsa-mir-1306-5p | MIMAT0022726 | GSR | 2936 | PAR-CLIP | 23446348 |
| hsa-mir-3529-3p | MIMAT0022741 | TP53 | 7157 | HITS-CLIP | 23824327 |
| hsa-mir-937-5p | MIMAT0022938 | TP53 | 7157 | PAR-CLIP | 23592263 |
| hsa-mir-939-3p | MIMAT0022939 | GSR | 2936 | HITS-CLIP | 23824327\|19536157 |
| hsa-mir-1233-5p | MIMAT0022943 | TP53 | 7157 | PAR-CLIP | 23592263 |
| hsa-mir-6086 | MIMAT0023711 | GSR | 2936 | PAR-CLIP | 23592263 |
| hsa-mir-6127 | MIMAT0024610 | TP53 | 7157 | PAR-CLIP | 23592263 |
| hsa-mir-6129 | MIMAT0024613 | TP53 | 7157 | PAR-CLIP | 23592263 |
| hsa-mir-6130 | MIMAT0024614 | TP53 | 7157 | PAR-CLIP | 23592263 |
| hsa-mir-6133 | MIMAT0024617 | TP53 | 7157 | PAR-CLIP | 23592263 |
| hsa-mir-6501-5p | MIMAT0025458 | GSR | 2936 | HITS-CLIP | 23824327 |
| hsa-mir-6511a-5p | MIMAT0025478 | GSR | 2936 | PAR-CLIP | 23592263 |
| hsa-mir-6722-3p | MIMAT0025854 | TP53 | 7157 | PAR-CLIP | 23592263 |
| hsa-mir-190a-3p | MIMAT0026482 | GSR | 2936 | HITS-CLIP//PAR-CLIP | 23446348\|21572407 |
| hsa-mir-433-5p | MIMAT0026554 | GSR | 2936 | PAR-CLIP | 27292025 |
| hsa-mir-1296-3p | MIMAT0026637 | TP53 | 7157 | PAR-CLIP | 23592263 |
| hsa-mir-548j-3p | MIMAT0026737 | GSR | 2936 | PAR-CLIP | 22100165 |
| hsa-mir-513b-3p | MIMAT0026749 | TP53 | 7157 | PAR-CLIP | 23592263 |
| hsa-mir-1910-3p | MIMAT0026917 | GSR | 2936 | PAR-CLIP | 23592263 |
| hsa-mir-6727-3p | MIMAT0027356 | GSR | 2936 | HITS-CLIP | 23824327\|19536157 |
| hsa-mir-6731-5p | MIMAT0027363 | TP53 | 7157 | PAR-CLIP | 23592263 |
| hsa-mir-6742-3p | MIMAT0027386 | GSR | 2936 | HITS-CLIP | 23824327\|19536157 |
| hsa-mir-6747-3p | MIMAT0027395 | GSR | 2936 | HITS-CLIP | 23824327\|19536157 |
| hsa-mir-6749-3p | MIMAT0027399 | TP53 | 7157 | HITS-CLIP | 23824327 |
| hsa-mir-6751-5p | MIMAT0027402 | TP53 | 7157 | PAR-CLIP | 23592263 |
| hsa-mir-6752-5p | MIMAT0027404 | TP53 | 7157 | PAR-CLIP | 23592263 |
| hsa-mir-6756-5p | MIMAT0027412 | TP53 | 7157 | PAR-CLIP | 23592263 |
| hsa-mir-6760-5p | MIMAT0027420 | TP53 | 7157 | PAR-CLIP | 23592263 |
| hsa-mir-6766-5p | MIMAT0027432 | TP53 | 7157 | PAR-CLIP | 23592263 |
| hsa-mir-6778-5p | MIMAT0027456 | TP53 | 7157 | PAR-CLIP | 23592263 |
| hsa-mir-6783-3p | MIMAT0027467 | GSR | 2936 | HITS-CLIP | 23824327\|19536157 |
| hsa-mir-6785-5p | MIMAT0027470 | TP53 | 7157 | PAR-CLIP | 23592263 |
| hsa-mir-6789-3p | MIMAT0027479 | GSR | 2936 | HITS-CLIP | 23313552 |
| hsa-mir-6790-3p | MIMAT0027481 | GSR | 2936 | HITS-CLIP | 23824327 |
| hsa-mir-6791-3p | MIMAT0027483 | GSR | 2936 | HITS-CLIP | 19536157 |
| hsa-mir-6797-5p | MIMAT0027494 | TP53 | 7157 | PAR-CLIP | 23592263 |
| hsa-mir-6802-3p | MIMAT0027505 | GSR | 2936 | HITS-CLIP | 23824327 |
| hsa-mir-6803-5p | MIMAT0027506 | TP53 | 7157 | PAR-CLIP | 23592263 |
| hsa-mir-6807-5p | MIMAT0027514 | CD36 | 948 | PAR-CLIP | 23446348 |
| hsa-mir-6821-3p | MIMAT0027543 | GSR | 2936 | HITS-CLIP | 23824327 |
| hsa-mir-6825-5p | MIMAT0027550 | TP53 | 7157 | PAR-CLIP | 23592263 |
| hsa-mir-6829-3p | MIMAT0027559 | GSR | 2936 | HITS-CLIP | 19536157 |
| hsa-mir-6835-5p | MIMAT0027570 | TP53 | 7157 | PAR-CLIP | 23592263 |
| hsa-mir-6780b-5p | MIMAT0027572 | TP53 | 7157 | PAR-CLIP | 23592263 |
| hsa-mir-6840-3p | MIMAT0027583 | GSR | 2936 | PAR-CLIP | 23592263 |
| hsa-mir-6842-5p | MIMAT0027586 | TP53 | 7157 | PAR-CLIP | 23592263 |
| hsa-mir-6852-5p | MIMAT0027604 | GSR | 2936 | HITS-CLIP | 23824327\|19536157 |
| hsa-mir-6865-3p | MIMAT0027631 | GSR | 2936 | HITS-CLIP | 23824327 |
| hsa-mir-6872-3p | MIMAT0027645 | CD36 | 948 | PAR-CLIP | 23446348 |
| hsa-mir-6872-3p | MIMAT0027645 | GSR | 2936 | HITS-CLIP | 23313552 |
| hsa-mir-6880-5p | MIMAT0027660 | TP53 | 7157 | HITS-CLIP | 19536157 |
| hsa-mir-6882-5p | MIMAT0027664 | TP53 | 7157 | HITS-CLIP | 19536157 |
| hsa-mir-6883-5p | MIMAT0027666 | TP53 | 7157 | PAR-CLIP | 23592263 |
| hsa-mir-6884-5p | MIMAT0027668 | GSR | 2936 | HITS-CLIP | 23706177 |
| hsa-mir-6890-3p | MIMAT0027681 | GSR | 2936 | HITS-CLIP | 19536157 |
| hsa-mir-7110-5p | MIMAT0028117 | TP53 | 7157 | PAR-CLIP | 23592263 |
| hsa-mir-7150 | MIMAT0028211 | TP53 | 7157 | PAR-CLIP | 23592263 |
| hsa-mir-7151-3p | MIMAT0028213 | CD36 | 948 | PAR-CLIP | 23446348 |
| hsa-mir-7151-3p | MIMAT0028213 | GSR | 2936 | HITS-CLIP | 23313552 |
| hsa-mir-6516-3p | MIMAT0030418 | CD36 | 948 | HITS-CLIP | 23824327 |
| hsa-mir-7855-5p | MIMAT0030430 | GSR | 2936 | PAR-CLIP | 23592263 |
| hsa-mir-8064 | MIMAT0030991 | CD36 | 948 | HITS-CLIP | 23824327\|23313552\|27418678 |
| hsa-mir-8071 | MIMAT0030998 | TP53 | 7157 | HITS-CLIP | 19536157 |
| hsa-mir-8085 | MIMAT0031012 | TP53 | 7157 | PAR-CLIP | 23592263 |
| hsa-mir-1249-5p | MIMAT0032029 | TP53 | 7157 | PAR-CLIP | 23592263 |
| hsa-mir-3653-5p | MIMAT0032110 | GSR | 2936 | HITS-CLIP | 23824327\|19536157 |
| hsa-mir-4485-5p | MIMAT0032116 | GSR | 2936 | HITS-CLIP | 23824327\|19536157 |
| hsa-mir-8485 | MIMAT0033692 | CD36 | 948 | HITS-CLIP | 23824327\|23313552\|27418678 |
| hsa-let-7d-5p | MIMAT0000065 | GSR | 2936 | HITS-CLIP, PAR-CLIP | tarbase |
| hsa-let-7e-5p | MIMAT0000066 | GSR | 2936 | HITS-CLIP, PAR-CLIP | tarbase |
| hsa-mir-129-5p | MIMAT0000242 | GSR | 2936 | HITS-CLIP | tarbase |
| hsa-mir-1302 | MIMAT0005890 | GSR | 2936 | PAR-CLIP | tarbase |
| hsa-mir-140-5p | MIMAT0000431 | GSR | 2936 | HITS-CLIP | tarbase |
| hsa-mir-15b-3p | MIMAT0004586 | GSR | 2936 | HITS-CLIP | tarbase |
| hsa-mir-188-3p | MIMAT0004613 | GSR | 2936 | HITS-CLIP | tarbase |
| hsa-mir-196b-3p | MIMAT0009201 | GSR | 2936 | HITS-CLIP | tarbase |
| hsa-mir-2114-5p | MIMAT0011156 | GSR | 2936 | HITS-CLIP | tarbase |
| hsa-mir-224-3p | MIMAT0009198 | GSR | 2936 | HITS-CLIP | tarbase |
| hsa-mir-22-5p | MIMAT0004495 | GSR | 2936 | HITS-CLIP | tarbase |
| hsa-mir-24-3p | MIMAT0000080 | GSR | 2936 | HITS-CLIP | tarbase |
| hsa-mir-30c-1-3p | MIMAT0004674 | GSR | 2936 | HITS-CLIP | tarbase |
| hsa-mir-30c-2-3p | MIMAT0004550 | GSR | 2936 | HITS-CLIP | tarbase |
| hsa-mir-328-3p | MIMAT0000752 | GSR | 2936 | HITS-CLIP | tarbase |
| hsa-mir-330-3p | MIMAT0000751 | GSR | 2936 | HITS-CLIP | tarbase |
| hsa-mir-362-5p | MIMAT0000705 | GSR | 2936 | PAR-CLIP | tarbase |
| hsa-mir-424-5p | MIMAT0001341 | GSR | 2936 | HITS-CLIP | tarbase |
| hsa-mir-497-5p | MIMAT0002820 | GSR | 2936 | HITS-CLIP | tarbase |
| hsa-mir-500b-5p | MIMAT0016925 | GSR | 2936 | PAR-CLIP | tarbase |
| hsa-mir-522-5p | MIMAT0005451 | GSR | 2936 | IMPACT-Seq | tarbase |
| hsa-mir-615-3p | MIMAT0003283 | GSR | 2936 | HITS-CLIP | tarbase |
| hsa-mir-627-5p | MIMAT0003296 | GSR | 2936 | HITS-CLIP | tarbase |
| hsa-mir-7-5p | MIMAT0000252 | GSR | 2936 | HITS-CLIP | tarbase |
| hsa-mir-769-3p | MIMAT0003887 | GSR | 2936 | PAR-CLIP | tarbase |
| hsa-mir-873-5p | MIMAT0004953 | GSR | 2936 | HITS-CLIP | tarbase |
| hsa-mir-124-3p | MIMAT0000422 | GSR | 2936 | RNA-Seq, Other, Microarrays | tarbase |
| hsa-mir-16-5p | MIMAT0000069 | GSR | 2936 | Microarrays | tarbase |
| hsa-mir-191-5p | MIMAT0000440 | GSR | 2936 | Microarrays | tarbase |
| hsa-mir-195-5p | MIMAT0000461 | GSR | 2936 | Microarrays | tarbase |
| hsa-mir-376a-5p | MIMAT0003386 | GSR | 2936 | Microarrays | tarbase |
| hsa-mir-103a-3p | MIMAT0000101 | CD36 | 948 | Microarrays | tarbase |
| hsa-mir-107 | MIMAT0000104 | CD36 | 948 | Microarrays | tarbase |
| hsa-mir-124-3p | MIMAT0000422 | CD36 | 948 | Microarrays | tarbase |
| hsa-mir-129-2-3p | MIMAT0004605 | CD36 | 948 | Microarrays | tarbase |
| hsa-mir-16-5p | MIMAT0000069 | CD36 | 948 | Microarrays | tarbase |
| hsa-mir-195-5p | MIMAT0000461 | CD36 | 948 | Microarrays | tarbase |
| hsa-mir-27a-3p | MIMAT0000084 | CD36 | 948 | Microarrays | tarbase |
| hsa-mir-29a-3p | MIMAT0000086 | CD36 | 948 | Microarrays | tarbase |
| hsa-mir-34b-5p | MIMAT0000685 | CD36 | 948 | Microarrays | tarbase |
| hsa-mir-671-5p | MIMAT0003880 | CD36 | 948 | Microarrays | tarbase |
| hsa-mir-374a-5p | MIMAT0000727 | CD36 | 948 | Microarrays | tarbase |
| hsa-let-7a-5p | MIMAT0000062 | TP53 | 7157 | HITS-CLIP | tarbase |
| hsa-let-7b-5p | MIMAT0000063 | TP53 | 7157 | HITS-CLIP, Microarrays | tarbase |
| hsa-let-7c-5p | MIMAT0000064 | TP53 | 7157 | HITS-CLIP | tarbase |
| hsa-let-7d-5p | MIMAT0000065 | TP53 | 7157 | HITS-CLIP | tarbase |
| hsa-let-7e-5p | MIMAT0000066 | TP53 | 7157 | HITS-CLIP | tarbase |
| hsa-let-7f-5p | MIMAT0000067 | TP53 | 7157 | HITS-CLIP | tarbase |
| hsa-let-7g-5p | MIMAT0000414 | TP53 | 7157 | HITS-CLIP | tarbase |
| hsa-let-7i-5p | MIMAT0000415 | TP53 | 7157 | HITS-CLIP | tarbase |
| hsa-mir-103a-3p | MIMAT0000101 | TP53 | 7157 | PAR-CLIP | tarbase |
| hsa-mir-107 | MIMAT0000104 | TP53 | 7157 | PAR-CLIP | tarbase |
| hsa-mir-122-5p | MIMAT0000421 | TP53 | 7157 | Biotin-Microarrays | tarbase |
| hsa-mir-1246 | MIMAT0005898 | TP53 | 7157 | qPCR, Western Blot | tarbase |
| hsa-mir-128-3p | MIMAT0000424 | TP53 | 7157 | HITS-CLIP, qPCR, Western Blot | tarbase |
| hsa-mir-181a-5p | MIMAT0000256 | TP53 | 7157 | HITS-CLIP | tarbase |
| hsa-mir-182-5p | MIMAT0000259 | TP53 | 7157 | qPCR | tarbase |
| hsa-mir-183-5p | MIMAT0000261 | TP53 | 7157 | PAR-CLIP | tarbase |
| hsa-mir-188-5p | MIMAT0000457 | TP53 | 7157 | HITS-CLIP | tarbase |
| hsa-mir-205-5p | MIMAT0000266 | TP53 | 7157 | Western Blot | tarbase |
| hsa-mir-21-3p | MIMAT0004494 | TP53 | 7157 | HITS-CLIP | tarbase |
| hsa-mir-218-5p | MIMAT0000275 | TP53 | 7157 | PAR-CLIP | tarbase |
| hsa-mir-22-3p | MIMAT0000077 | TP53 | 7157 | HITS-CLIP | tarbase |
| hsa-mir-27b-3p | MIMAT0000419 | TP53 | 7157 | HITS-CLIP | tarbase |
| hsa-mir-29a-3p | MIMAT0000086 | TP53 | 7157 | Western Blot | tarbase |
| hsa-mir-29b-3p | MIMAT0000100 | TP53 | 7157 | Western Blot | tarbase |
| hsa-mir-29c-3p | MIMAT0000681 | TP53 | 7157 | Western Blot | tarbase |
| hsa-mir-361-3p | MIMAT0004682 | TP53 | 7157 | HITS-CLIP | tarbase |
| hsa-mir-362-5p | MIMAT0000705 | TP53 | 7157 | HITS-CLIP | tarbase |
| hsa-mir-3657 | MIMAT0018077 | TP53 | 7157 | HITS-CLIP | tarbase |
| hsa-mir-500b-5p | MIMAT0016925 | TP53 | 7157 | HITS-CLIP | tarbase |
| hsa-mir-522-5p | MIMAT0005451 | TP53 | 7157 | IMPACT-Seq | tarbase |
| hsa-mir-766-3p | MIMAT0003888 | TP53 | 7157 | HITS-CLIP | tarbase |
| hsa-mir-93-5p | MIMAT0000093 | TP53 | 7157 | PAR-CLIP | tarbase |
| hsa-mir-940 | MIMAT0004983 | TP53 | 7157 | HITS-CLIP | tarbase |
| hsa-mir-9-5p | MIMAT0000441 | TP53 | 7157 | HITS-CLIP | tarbase |
| hsa-mir-98-5p | MIMAT0000096 | TP53 | 7157 | HITS-CLIP | tarbase |
| hsa-mir-1-3p | MIMAT0000416 | TP53 | 7157 | RNA-Seq | tarbase |
| hsa-mir-23b-3p | MIMAT0000418 | TP53 | 7157 | RNA-Seq | tarbase |
| hsa-mir-155-5p | MIMAT0000646 | TP53 | 7157 | Microarrays | tarbase |
| hsa-mir-191-5p | MIMAT0000440 | TP53 | 7157 | Microarrays | tarbase |
| hsa-mir-203a-3p | MIMAT0000264 | TP53 | 7157 | Microarrays | tarbase |
| hsa-mir-210-3p | MIMAT0000267 | TP53 | 7157 | Microarrays | tarbase |
| hsa-mir-212-3p | MIMAT0000269 | TP53 | 7157 | Microarrays | tarbase |
| hsa-mir-148b-3p | MIMAT0000759 | TP53 | 7157 | Microarrays | tarbase |
| hsa-mir-103a-3p | MIMAT0000101 | NQO1 | 1728 | HITS-CLIP | tarbase |
| hsa-mir-107 | MIMAT0000104 | NQO1 | 1728 | HITS-CLIP | tarbase |
| hsa-mir-186-5p | MIMAT0000456 | NQO1 | 1728 | PAR-CLIP | tarbase |
| hsa-mir-24-3p | MIMAT0000080 | NQO1 | 1728 | HITS-CLIP | tarbase |
| hsa-mir-338-5p | MIMAT0004701 | NQO1 | 1728 | HITS-CLIP | tarbase |
| hsa-mir-34a-5p | MIMAT0000255 | NQO1 | 1728 | Microarrays | tarbase |
| hsa-mir-375 | MIMAT0000728 | NQO1 | 1728 | HITS-CLIP, Microarrays | tarbase |
| hsa-mir-485-5p | MIMAT0002175 | NQO1 | 1728 | HITS-CLIP | tarbase |
| hsa-mir-1-3p | MIMAT0000416 | NQO1 | 1728 | RPF-Seq | tarbase |
| hsa-mir-124-3p | MIMAT0000422 | NQO1 | 1728 | Microarrays | tarbase |
| hsa-mir-126-3p | MIMAT0000445 | NQO1 | 1728 | Microarrays | tarbase |
| hsa-mir-128-3p | MIMAT0000424 | NQO1 | 1728 | Microarrays | tarbase |
| hsa-mir-129-2-3p | MIMAT0004605 | NQO1 | 1728 | Microarrays | tarbase |
| hsa-mir-147a | MIMAT0000251 | NQO1 | 1728 | Microarrays | tarbase |
| hsa-mir-200c-3p | MIMAT0000617 | NQO1 | 1728 | Microarrays | tarbase |
| hsa-mir-205-5p | MIMAT0000266 | NQO1 | 1728 | Microarrays | tarbase |
| hsa-mir-210-3p | MIMAT0000267 | NQO1 | 1728 | Microarrays | tarbase |
| hsa-mir-27a-3p | MIMAT0000084 | NQO1 | 1728 | Microarrays | tarbase |
| hsa-mir-7-5p | MIMAT0000252 | NQO1 | 1728 | Microarrays | tarbase |
| hsa-mir-941 | MIMAT0004984 | NQO1 | 1728 | Microarrays | tarbase |
| hsa-mir-374a-5p | MIMAT0000727 | NQO1 | 1728 | Microarrays | tarbase |
| hsa-mir-1226-5p | MIMAT0005576 | GPX3 | 2878 | HITS-CLIP | tarbase |
| hsa-mir-30a-5p | MIMAT0000087 | GPX3 | 2878 | HITS-CLIP | tarbase |
| hsa-mir-30b-5p | MIMAT0000420 | GPX3 | 2878 | HITS-CLIP | tarbase |
| hsa-mir-30c-1-3p | MIMAT0004674 | GPX3 | 2878 | HITS-CLIP | tarbase |
| hsa-mir-30c-2-3p | MIMAT0004550 | GPX3 | 2878 | HITS-CLIP | tarbase |
| hsa-mir-30c-5p | MIMAT0000244 | GPX3 | 2878 | HITS-CLIP | tarbase |
| hsa-mir-30d-5p | MIMAT0000245 | GPX3 | 2878 | HITS-CLIP | tarbase |
| hsa-mir-30e-5p | MIMAT0000692 | GPX3 | 2878 | HITS-CLIP | tarbase |
| hsa-mir-876-5p | MIMAT0004924 | GPX3 | 2878 | HITS-CLIP | tarbase |
| hsa-mir-1-3p | MIMAT0000416 | GPX3 | 2878 | RPF-Seq, RNA-Seq | tarbase |
| hsa-let-7b-5p | MIMAT0000063 | GPX3 | 2878 | Microarrays | tarbase |
| hsa-mir-124-3p | MIMAT0000422 | GPX3 | 2878 | Microarrays | tarbase |
| hsa-mir-128-3p | MIMAT0000424 | GPX3 | 2878 | Microarrays | tarbase |
| hsa-mir-129-2-3p | MIMAT0004605 | GPX3 | 2878 | Microarrays | tarbase |
| hsa-mir-155-5p | MIMAT0000646 | GPX3 | 2878 | Microarrays | tarbase |
| hsa-mir-16-5p | MIMAT0000069 | GPX3 | 2878 | Microarrays | tarbase |
| hsa-mir-182-5p | MIMAT0000259 | GPX3 | 2878 | Microarrays | tarbase |
| hsa-mir-191-5p | MIMAT0000440 | GPX3 | 2878 | Microarrays | tarbase |
| hsa-mir-20a-5p | MIMAT0000075 | GPX3 | 2878 | Microarrays | tarbase |
| hsa-mir-449a | MIMAT0001541 | GPX3 | 2878 | Microarrays | tarbase |
| hsa-mir-374a-5p | MIMAT0000727 | GPX3 | 2878 | Microarrays | tarbase |
| hsa-mir-1285 | MIMAT0005876 | TP53 | 7157 | Western blot | 20417621 |
